# A Review of Recent Distributed Optical Fiber Sensors Applications for Civil Engineering Structural Health Monitoring

**DOI:** 10.3390/s21051818

**Published:** 2021-03-05

**Authors:** Mattia Francesco Bado, Joan R. Casas

**Affiliations:** 1 Department of Civil and Environmental Engineering, Technical University of Catalonia (UPC), Jordi Girona 1–3, 08034 Barcelona, Spain; joan.ramon.casas@upc.edu; 2 Department of Reinforced Concrete Structures and Geotechnical Engineering, Vilnius Gediminas Technical University, Saulėtekio al. 11, 10221 Vilnius, Lithuania

**Keywords:** distributed optical fiber sensors, distributed optical fiber sensors, distributed sensing, distributed monitoring, DOFS, DFOS, structural health monitoring, SHM, review

## Abstract

The present work is a comprehensive collection of recently published research articles on Structural Health Monitoring (SHM) campaigns performed by means of Distributed Optical Fiber Sensors (DOFS). The latter are cutting-edge strain, temperature and vibration monitoring tools with a large potential pool, namely their minimal intrusiveness, accuracy, ease of deployment and more. Its most state-of-the-art feature, though, is the ability to perform measurements with very small spatial resolutions (as small as 0.63 mm). This review article intends to introduce, inform and advise the readers on various DOFS deployment methodologies for the assessment of the residual ability of a structure to continue serving its intended purpose. By collecting in a single place these recent efforts, advancements and findings, the authors intend to contribute to the goal of collective growth towards an efficient SHM. The current work is structured in a manner that allows for the single consultation of any specific DOFS application field, i.e., laboratory experimentation, the built environment (bridges, buildings, roads, etc.), geotechnical constructions, tunnels, pipelines and wind turbines. Beforehand, a brief section was constructed around the recent progress on the study of the strain transfer mechanisms occurring in the multi-layered sensing system inherent to any DOFS deployment (different kinds of fiber claddings, coatings and bonding adhesives). Finally, a section is also dedicated to ideas and concepts for those novel DOFS applications which may very well represent the future of SHM.

## 1. Preface

The present work reports numerous Distributed Optical Fiber Sensors (DOFS) monitoring campaigns spread over multiple Structural and Civil Engineering fields. Whilst a sequential reading of the article’s diverse sections may provide a global point of view on the evolution of DOFS for the Structural Health Monitoring (SHM) panorama, the authors are intent on also providing the readers with the possibility of skipping directly to the section of interest.

Section 2*. Introduction* provides a very general overview on the topic of SHM and the importance of the implementation of DOFS. Before presenting the most recently published DOFS-SHM integrations, Section 3*. DOFS Coatings and Bonding Adhesives: The Strain Transfer Process and the Optimal Bonding Technique* tackles an issue of great relevance which intrinsically affects any DOFS monitoring. The latter, as foreshadowed by the section’s title, is the strain transfer process occurring in the multi-layered sensing system that characterizes a bonded DOFS. In more detail, due to the stiffness diversity of the adhesives, cladding and coating layers that separate a DOFS’ sensing core from a structural host surface, a certain distortion of the sampled data occurs which, in return, decreases the reliability of the DOFS measured strain. The authors deemed mandatory reporting some recently published findings on this crucial and intrinsic limitation before reporting any in-situ DOFS-monitoring campaign.

Finally, the reported DOFS-SHM applications are separated into the following sections:

- Section 4. Laboratory Experimental Applications
- Section 5. The Built Environment: Buildings, Bridges and Roads
- Section 6. Geotechnical Engineering
- Section 7. Tunnel
- Section 8. Pipes
- Section 9. Wind Turbines

Finally, *Section 10. An Eye towards the Future* introduces the reader to those DOFS-SHM novelties that may very well become key DOFS applications in the future.

Acknowledging the broadness of the above-listed application fields and the readers’ engineering backgrounds differences, for the proper understanding of all the DOFS applications, the authors started each section and subsection with a small introduction aimed at providing a certain degree of contextualization. At the end of each section, instead, a brief summary will be presented encapsulating the key findings and DOFS deployments tips.

Furthermore, the authors would like to remark that the hereafter reported figures will all be taken from the referred articles and are, therefore, uniquely the product of their respective writers’ efforts. In line with the concept of research and its goal of collective growth towards the solution of society’s biggest problems, the figures were selected with the aim of clarifying the reported DOFS deployment techniques and their outputs in addition to sharing any particular ingenious technique that other monitoring campaigns might draw inspiration from.

Finally, the present work situates itself as a spiritual successor to the work developed by Barrias et al. [1] which studied the DOFS-SHM integrations until 2016, the year of publication. Consequently, the present review article deals with the publications comprised in the time interval 2017–2020 additionally expanding on previously overlooked topics.

## 2. Introduction

As clearly stated by Brownjohn [2], civil infrastructure provides the means for a society to function. The wide range of applications that Civil Engineering offers includes buildings, bridges, highways, tunnels, power plants, industrial facilities, geotechnical, hydraulic structures and more. Throughout their service lifetimes, each of these is subjected to multiple events that deteriorate and compromise their structural integrity thus exposing their future performances to several risks. In order to avoid the social, economic and environmental costs of their deterioration and/or failure, civil structures have different safety and durability requirements that need to be met. In order to ensure correct structural performance and the wellbeing of their users, a series of inspections, monitoring and maintenance protocols have to be carried out during the structures’ service lifetimes. The effectiveness of these monitoring methods relies on their ability to rapidly detect alterations of the structure’s performance, allowing for their identification, characterization and control. This idea is the basis of Structural Health Monitoring (SHM). Indeed, according to Housner et al. [3], SHM can be understood as the continuous and regular measurement and analysis of key structural and environmental parameters under operating conditions for the purpose of warning of any early-stage abnormal structural behaviors.

Yet, as stated by Cawley [4], despite the recently increasing volume of research on SHM, this enormous effort has yielded only a small number of routine industrial applications. Indeed, still at the present day, the most common manner of assessing an infrastructure’s ability to carry on with its designed duties is through the intervention of engineers trained in structural visual inspections [1]. A non-disregardable degree of inaccuracy is therefore automatically incorporated in these studies due to the differences in the inspectors’ safety condition assessment backgrounds. Wherever monitoring tools are additionally employed, these usually fall under the category of “traditional monitoring techniques” such as inclinometers, accelerometers, extensometers, total station surveys, load cells and GNS-based sensors. By themselves, these can be considered sufficiently reliable since their correct deployment has been extensively investigated and they are nowadays widely acknowledged, thus ensuring reliable monitoring and structural assessments. However, as stated in Baker [5], conventional forms of inspection and monitoring are only as good as their ability to uncover potential issues in an accurate and timely manner. Indeed, regarding the ability of damage detection, traditional tools present several drawbacks which are, amongst others, insufficient data management ability, the need for the infrastructure’s service interruption during their deployment, non-automated real-time measurements and interference risk. The above deficiencies, stacked up with the lack of reliable, scalable and affordable monitoring solutions and the absence of uniform execution methodologies [6] and some degree of standardization, are the reason why SHM is not yet implemented as a standard practice in most Civil Engineering structures.

Modern monitoring technologies are aimed at tackling the above-mentioned limitations thus boosting the sensors’ precision, automation and data management speed. These include amongst others Optical Fiber Sensors (OFS), Global Positioning Systems (GPS), radars, Micro Electro Mechanical Systems (MEMS) and Image Processing Techniques such as Digital Image Correlation (DIC). As a matter of fact, as properly put by Seo [7], Civil Engineering has recently seen a convergence with various other fields, thus slowly moving towards technology convergence. The latter is a theory asserting the absence of a clear distinctive line between the products/applications of different disciplines but rather a co-existence, intersection and reciprocal enhancement between the latter which, in return, accelerates the overall technological advancement [8]. SMART monitoring is one of these developments and the use of OFS in Civil Engineering can be considered a quintessential example as it joins Civil Engineering, Photonics, Materials Engineering and Computer Engineering.

**Figure 1:**
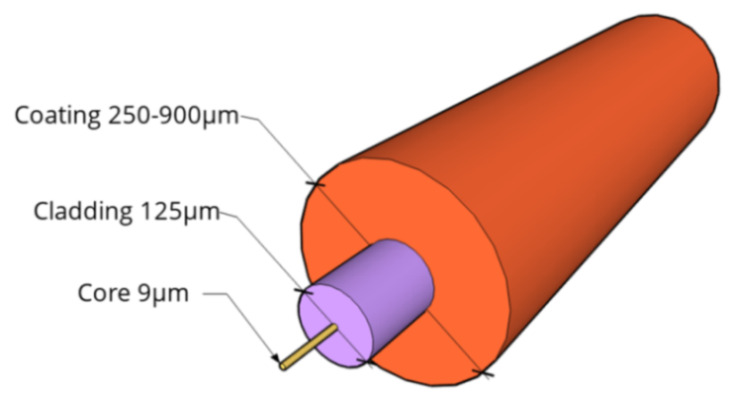
3D illustration of an optical fiber cross-section.

Focusing in particular on OFS, these are dielectric devices used to confine and guide light. They consist of several layers: fiber core, cladding and, occasionally, an external jacket aimed at providing mechanical resistance to the fiber (an example is provided in Figure 1).

The majority of OFS used in sensing applications have silica (SiO_2_) glass cores and claddings. The refractive index of the latter is lower than that of the core in order to satisfy the condition of Snell’s law for total internal reflection and thus confine the propagation of the light along the fiber core only [9]. The jacket is usually made of polymeric material or nylon and can vary in diametrical size, shape and manufacturing process. Finally, as will be evident in this article, the external coating can be constituted by numerous kinds of materials, layering, thicknesses and external textures.

A significant ability of OFS is the ability to measure mechanical and temperature-variation-induced strains along the fiber length by means of light scattering and back-scattering occurring whenever the photons of the emitted light interact with the physical medium through which it travels (the fiber’s core itself). When no strain or temperature is imposed on the system, light propagates and gets reflected throughout the imperfections with a given signature. Instead, when strain or temperature values vary, the frequency of the back scattered light is shifted. The measured strain values are then related to the frequency shift.

As a matter of fact, three different types of light scattering and backscattering may occur in an OFS, namely Raman, Brillouin and Rayleigh. All hold particular optical features that make one more suitable than the others relative to the research objectives. Brillouin scattering, thanks to its extended measurement range capability (up to several kilometers), is the most studied and used OFS system in Civil and Geotechnical Engineering. The Raman back-scattering, instead, is characterized by a high dependence on temperature but can also be used to extract physical and chemical information of a material [10] such as food quality [11] or presence of explosive materials [12]. The third back-scattering phenomenon is Rayleigh. Despite its 70 m sensing range limit, it provides the highest spatial resolution of the three (even down to 0.63 mm), which is ideal for strain and damage monitoring in the context of environmentally controlled experimental laboratory investigation.

Whilst multiple sections of the present article could be dedicated to the explanation of the different photonic working principles of all the commercially available kinds of OFS, this topic has been discussed innumerable times in almost every OFS application article both new and old. Therefore, the authors believe its insertion would result in redundancy. In its stead, Table 1 not only summarizes this article’s most commonly used acronyms but also refers the reader to exhaustive literature on each different kind of OFS photonic working principle (marked with the symbol * in Table 1).

Table 2, instead, reports a concise summary of the performance of the various distributed sensing techniques thus providing the reader with an idea of the strengths and weaknesses of each.

Finally, for additional reading on the subject, the authors refer the readers to the comprehensive state-of-the-art reviews on FOS, namely Udd [33], Kersey [34] and Grattan and Sun [35].

**Figure 2:**
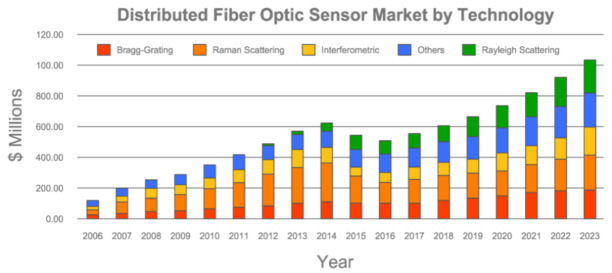
Growth of Optical Fiber Sensors (OFS) technological market [36].

OFS sensors have recently improved their performance, drastically increasing their potential pool. The latter was immediately picked up and tapped into by the worldwide scientific community as evident from the increasingly larger number of relative published research articles. Additionally, according to a market survey report conducted by the Photonic Sensor Consortium and published by Information Gatekeepers [36], the increased OFS popularity goes hand in hand with the monitored and predicted growth of the whole OFS fiber sensors technological market (see Figure 2).

It is worthy of note that, according to Figure 2, the combined distributed and single point fiber optic sensor markets are projected to be over USD 1.3 billion in 2023. In this respect, the present work represents an effort aimed at facilitating the use of OFS for both the present and prospective users of this novel technology.

Now, if to focus strictly on OFS’s application to the Civil and Structural Engineering field, it can easily be asserted that it has slowly but steadily increased in the past years [1,37]. As clearly identified by Li et al. [9], its increased popularity for SHM applications can be attributed to the following OFS features:

- They allow for completely distributed monitoring (in the case of DOFS) with monitoring points spaced less than 1 mm;
- Their small diameter and minimal stiffness allow for a very high degree of deployment configuration complexity, no matter if these imply circumferential surfaces, sharp corners, surface irregularities and more. It is even possible to embed them inside structural elements with a minimal level of intrusiveness;
- Their ease of deployment as can be achieved by simply applying an adhesive over it.
- Their monitoring length is very flexible and can vary from halves of millimeter to tens of kilometers;
- Intrinsic immunity to electro-magnetic interference;
- They are designed with a long life cycle in mind. Indeed, its main component, silica, is highly resistant to corrosion and can withstand high tensile loading;
- Silica core OFS are highly resistant to temperature and can measure temperatures from −200 °C to 800 °C.

The great majority of photonic sensing technology applied to SHM is constituted by discrete sensors such as Fiber Bragg Gratings (FBG) [9] as these were introduced to the market much earlier than the undoubtedly superior DOFS. FBG are quasi-distributed optical fiber sensors in which a characteristic wavelength is used to simultaneously provide its address in the sensor network, and the measurement (temperature and strains) [31]. Their applications can be dated back as early as 1993, when Intelligent Sensing for Innovative Structures performed fiber optic deployments on six bridges in Canada [38] or in 2001 when four FBG sensors were installed across, above and under the primary arch of Como’s Cathedral in order to identify any significant structural deterioration [39]. It can be easily argued that the discrete sensors’ (such as FBG) most crucial limitation lies in their non-distributed measurement nature. This shortcoming is quite critical in the context of SHM as it prevents the possibility of precisely pointing out the location where a potential damage first occurs and prevents the linking of local damage mechanisms to the global condition of the structure.

**Figure 3:**
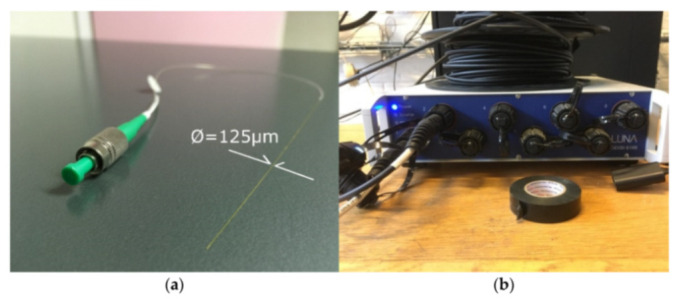
(**a**) Distributed OFS (DOFS) fiber and (**b**) ODiSI-6000 model Optical Backscatter Reflectometer (OBR) interrogator manufactured by LUNA Technologies.

The lack of sufficient monitoring points along a fiber deployment (spatial resolution) is an issue entirely surpassed by DOFS (Figure 3 left). As a matter of fact, the latest model of OFDR Optical Backscatter Reflectometer (OBR) interrogator machines (such as the one on the right side of Figure 3) is able to monitor strains with a spatial resolution of 0.63 mm.

The distributed nature of DOFS enables the mapping of temperature, strain and vibration distributions in two or even three dimensions (achievable with a DOFS mesh deployment) and their identification at any point along a fiber. Combined, these aspects allow for the painting of a clear picture of the global behavior of a structure rather than reporting the tensile state of a limited number of points. Figure 3 also displays the hair-like thinness of the fiber which makes it ideal for un-intrusive deployments. Still, as suggested by Barrias [1], this is still a recent and developing technology as can be perceived by the relatively scarce number of DOFS applications in SHM projects published in research articles.

Despite such, a discrete variety of applications have been performed including bridges, dams, tunnels, pipelines and slopes. The goal of the present article is to shine a spotlight on the most recent of such DOFS SHM applications and help the collective growth towards an efficient SHM by collecting in a single place these recent efforts, advancements and findings. In particular, as mentioned in Section 1, the reported DOFS SHM applications include laboratory experimentation, the built environment (bridges, buildings, roads, etc.), geotechnical constructions, tunnels, pipelines and wind turbines, all performed in the time frame 2017–2020.

## 3. DOFS Coatings and Bonding Adhesives: The Strain Transfer Process and the Optimal Bonding Technique

**Figure 4:**
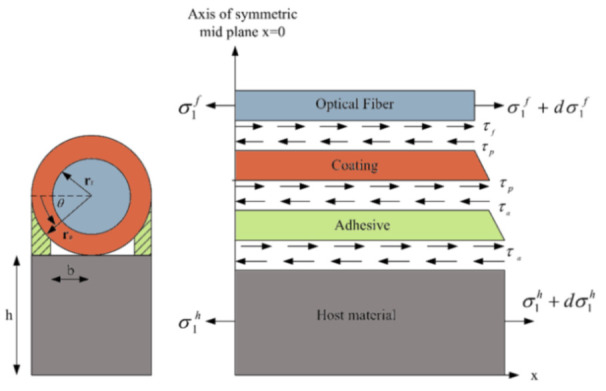
Free body diagram for the symmetrical section of the optical fiber and the substrates together with their relative shear transfer (adapted from [40]).

Ideally, the strains measured by a DOFS silica core should be equivalent to the actual strains present in the host structure on which the former is bonded. If this requirement was fully met then both DOFS structural monitoring and laboratory structural investigation would simply boil down to analyzing the OBR’s output data, no matter how minute the object of investigation is or how accurate the measurement requirements are. Unfortunately, this is not always the case. Indeed, the packaging and the installation of the sensors involves intermediate layers (i.e., protective coatings and adhesive layers) whose presence leads to the failure of the complete strain transfer from the host material to the sensing fiber [19]. Instead, some of the energy is converted to the shear and normal deformations of the intermediate layers (as well illustrated by Her and Huang [40] in Figure 4) thus leading to strain transfer errors.

The reader should consider that Figure 4, for the sake of simplicity, only represented a single coating layer between the fiber core and the adhesive but in numerous applications, the intermediate layers are many more, i.e., cladding, reinforcement layers, diverse coatings, jackets, etc. The whole issue is exacerbated in the presence of strain singularities zones where a high-stress concentration is accumulated over a short length of DOFS, such as cracks in RC elements or structural discontinuities. Because the optical fiber bridges the cracks irrespective of whether they are embedded or surface adhered, in order to eliminate this error and guarantee effective measurements, investigation on the strain transfer mechanism from the substrate material (steel, concrete, etc.) to the fiber core is required [19,41]. Alj et al. [42], for example, indicated that the strain transfer process depended on mechanical and geometrical properties of the different intermediate layers, i.e., their elastic modulus, their height and the initial crack opening.

Alj et al. continued reporting that several analytical models had been proposed in the literature among which the one of Feng et al. [41] who developed a theoretical approach to describe the strain transfer process in zones containing strain singularities (cracks) and the one of Henault et al. [43] who defined a Mechanical Transfer Function (MTF) that relates the strain profile along the DOFS core to the strain profile of the host cracked concrete. The above analyses become especially relevant when considering the need of adding additional protective layers around a DOFS core. Indeed, whilst the ideal DOFS would be one with the least and thinnest cladding/coating layer possible, in practice, considering the challenging environments where the fiber could be deployed, most commercial DOFS cables include additional coating layers that protect the core against mechanical and chemical damages. Furthermore, the high-stress concentration transmitted to a DOFS by a crack opening may lead to its premature failure leading to the loss of data from that instance on.

Whenever an experimental investigation requires the maximum precision possible, the shear lag induced by any host-surface/DOFS-core intermediate layers should be minimal, thus the preferable fiber is a simply cladded non-coated DOFS. In this case, though, the protective function against external damages falls entirely on the adhesive layers. Numerous experimental campaigns have been performed in the latest year to assess which is the optimal adhesive for any specific function, i.e., bonded to a structure’s surface or embedded inside the structure itself bonded to rebar, for instance.

As can be evinced, the optimal DOFS claddings/coatings and bonding techniques are still elusive concepts and, therefore, still under heavy investigation. The authors believe in the importance of reporting the recent discoveries and discussions on the present topic first as it inherently influences every DOFS monitoring campaign henceforth.

Wang et al. [19] recently developed a state-of-the-art review on the strain transfer theory of optical fiber-based sensors developed for Civil structures. In particular, the article addresses the issues of strain not entirely transferred from the host material to the sensing fiber, resulting in a portion of strain loss in the transferring path and the Strain Transfer Theory produced to tackle this issue. Interestingly the strain transfer theory was reported for both embedded and surface-attached sensors. The authors concluded by mentioning that the Strain Transfer Theory could be adopted to conduct accurate interpretations of the collected data, to analyze the influence of the installed sensors on the structural integrity and strength and to improve the design and calibration codes of the sensors for the long-term, effective and accurate monitoring.

**Figure 5:**
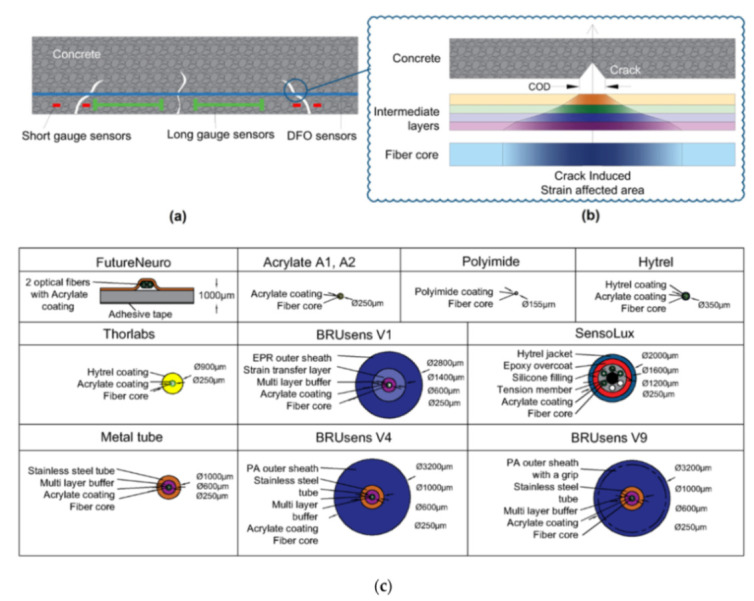
Crack detection using DOFS techniques: (**a**) Comparison of the traditional sensors, (**b**) strain transferring between layers and (**c**) the tested cables (adapted from [44]).

Bassil et al. [44] studied the above-mentioned strain transfer mechanism and proposed a new analytical model which assumes the absence of a perfect bond between the components of a multilayer system. Particularly, a general expression of the crack-induced strain transfer from fractured concrete material to an optical fiber (see Figure 5a,b) is established and put to the test through wedge splitting tests on concrete specimens instrumented with multiple embedded and surface-mounted fiber optic cables.

Furthermore, the authors summarized the modern-day expertise on DOFS cables:

- Different types of optical cables are available on the market;
- Some cables are conceived to be embedded inside the structure during construction, whilst others are more suitable for surface installation;
- The cables, constituted of different materials and shapes, lead to a different strain transfer response due to the shear lag effect in the intermediate layers;
- Little information is available on the mechanical properties of the cable and its constituent elements;
- Little information is also available on the long-term behavior and performance of these cables.

In response to some of the above lackings, the authors used in their test multiple kinds of OFDR DOFS cables (see Figure 5c). The experimental results reportedly estimated correctly the crack openings and led to successful monitoring of the optical cable responses through the strain lag parameter. A final key observation worth reporting here was the Polyimide-cladded cables’ strain lag parameters λ varying around 260 m^−1^ whilst the others between 20 m^−1^ and 60 m^−1^. This meant that the Polyimide fiber reported crack-induced strain distributions 5 to 15 times higher than the other tested cables, which allowed for the accurate detection of tiny crack propagations.

Zhang et al. [45] proposed a mechanical model (further validating with experimentation) to study the effects of multiple closely spaced cracks on the DOFS measurements and the effects of the cable properties (i.e., shear stiffness between cable and fiber, cable radius, elastic modulus, interface cohesion) on the shape of the DOFS strain distributions across cracks. The reported spring model was constituted by two layers, namely an inner one (representing the fiber core and cladding) and an outer one (representing the outer coating, primary layers and a jacket) with assigned relative stiffness. A noteworthy result was that, by independently varying the stiffnesses in the spring model, the axial stiffness of the coating layer was reported to have a negligible influence on the shape of strain distribution. This was the opposite for the coatings shear stiffness and shear parameter.

Alj [46] developed his doctoral thesis in the framework of the ODOBA (Observatory of the Durability Of reinforced concrete structures) project launched by IRSN in 2016 in France which was aimed at studying the concrete pathologies and their consequences for nuclear power plant structures. In particular, the thesis was redacted with the objective of studying the effectiveness of DOFS technology to detect and monitor concrete pathologies potentially met in nuclear power plants (Delayed Ettringite Formation, Alkali-Silica Reaction or a combination of the two). As such, the work also undertook the interpretation of long-term strain measurements provided by OF sensors in degrading environments.

The above project spawned a research article on the topic of the durability of DOFS [47]. Here, the authors studied the durability of OFDR DOFS cables for the SHM of Reinforced Concrete (RC) structures through an experimental campaign based on the accelerated aging of small concrete specimens instrumented with two embedded optical fiber cables. The first included an external coating made of a soft elastomeric material (olefin-type elastomer) containing two single-mode OFs and two metal rods for mechanical protection. The second one included an outer layer, made of polyethylene with six optical fibers (single-mode and multimode) which are wound around a central rod (reinforcement part of the cable) and embedded in an elastomeric layer. In order to simulate the effect of alkaline pore solution of the concrete medium, the accelerated aging was achieved immerging the specimens in an alkaline solution: c(KOH) = 0.5 mol/L + c(NaOH) = 0.1 mol/L, at three different temperatures (20 °C, 40 °C and 60 °C) for three months. Pull-out tests were consequently carried out in order to evaluate the effect of the accelerated aging on the mechanical response of the cable/concrete interfaces. The results showed that the interface between the first cable and the concrete had not been degraded, contrariwise, the second cable’s interface was damaged and the degradation was emphasized by temperature.

The results of the previous test are a demonstration of the large experimental effort still required by the scientific community to fully dominate the DOFS-powered SHM monitoring science.

**Figure 6:**
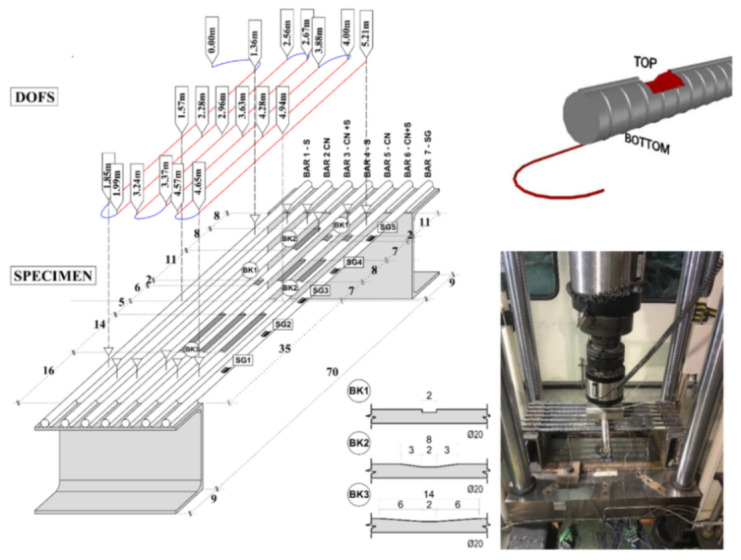
Test setup: three-dimensional specimen detailing and photography [48].

Moving on to the second topic of the present section, specifically the research of the optimal adhesive and bonding technique for the RC structures instrumentation with non-coated OFDR DOFS. Bado et al. [48] asserted that despite DOF’s potential for the detection of deformations and cracking in RC structures, in numerous published DOFS-monitored experimental campaigns, researchers reported the appearance of unrealistic strain readings, defined as Strain Reading Anomalies (SRAs), which prevented the extraction of reliable strain data. Later, the article outlined the results obtained through an experimental test aimed at inducing such SRAs in an isolated and controlled environment in order to isolate and identify the physical cause of their origin. The test consisted of gradually bending seven steel reinforcement bars with DOFS bonded to their bottom side with different adhesives, namely cyanoacrylate, silicone, and a combination of the two. Additionally, one of the rebars was instrumented with Strain Gauges (SG) for comparison purposes. Three of the DOFS instrumented rebars were transversally incised (as visible in Figure 6) in order to simulate the typical post-cracking strain transmission between concrete and steel.

These incisions further had varying steepness degrees in order to simulate different transmission lengths. Finally, different loading speeds were applied (rapid/impact and slow monotonic one). The results showed the bonding adhesives having an influence on the DOFS performance but not on the rise of anomalies. Expectedly, considering the difference between the modulus of elasticity of silicone and cyanoacrylate, the former seemed to delay the appearance of SRAs to the expense of the strain measurement precision in addition to redistributing the strains along the fiber. Oppositely, the rigid but fragile nature of the cyanoacrylate reportedly provided the results with superior precision but at the expense of the adhesive resistance and endurance. The combination of the two provided a hybrid behavior. Finally, the reasons triggering the SRAs were narrowed down to a strain threshold and to the friction with concrete present whenever the DOFS is embedded inside an RC structure.

This last conclusion was later demonstrated to be the main reason for the rise in SRAs in Bado et al. [49]. The article was aimed at providing a contribution to the effort of establishing the best thin non-coated OFDR DOFS/rebar bonding technique whenever the instrumented bar is to be embedded inside of RC structures. The investigated bonding techniques were:

- DOFS positioned inside a groove and bonded with cyanoacrylate;
- DOFS glued to the rebar’s surface with cyanoacrylate with a protective layer of silicone on top;
- DOFS positioned inside a groove, bonded with cyanoacrylate and further covered with silicone.

**Figure 7:**
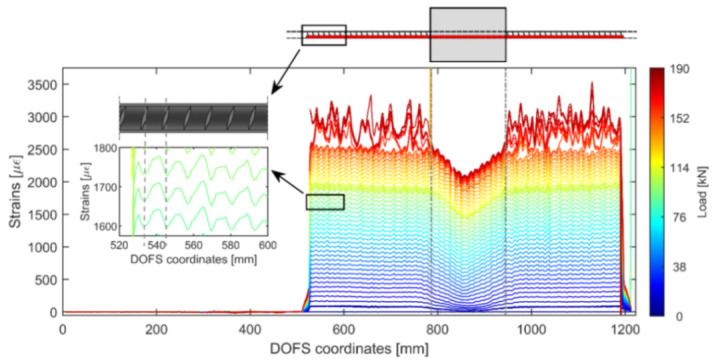
Strain profiles of a 15 × 15 × 210 mm Ø20 Reinforced Concrete (RC) tie test with the DOFS bonded with a combination of groove, cyanoacrylate and silicone [49].

The authors also indicated as a crucial requirement the absence of any influence of the bonding technique on the DOFS sampled strains, thus, the guarantee that what is actually reported by the fiber are the actual strains occurring in the monitored surface rather than a strain present solely in the fiber. The first phase of the test (performed on a bare rebar) assessed that the presence of a layer of silicone and/or the one of a groove had no influence on the extrapolated DOFS when compared to readings performed with a DOFS simply bonded to the rebar with cyanoacrylate. This was proved to be valid both in tension and in bending. The second phase of the test saw the embedment of the instrumented rebars inside a prism of concrete to form multiple RC tensile elements (also named RC ties). These RC specimens were of two kinds, cracking and non-cracking ones designed expressly to study these two different scenarios. Interestingly, during this second test phase, the authors were able to monitor the steel rebars’ strains well beyond the yield limit as testified by the peaks of Figure 7 which are the physical representation of the rebar’s different sections slowly plasticizing one after the other.

**Figure 8:**
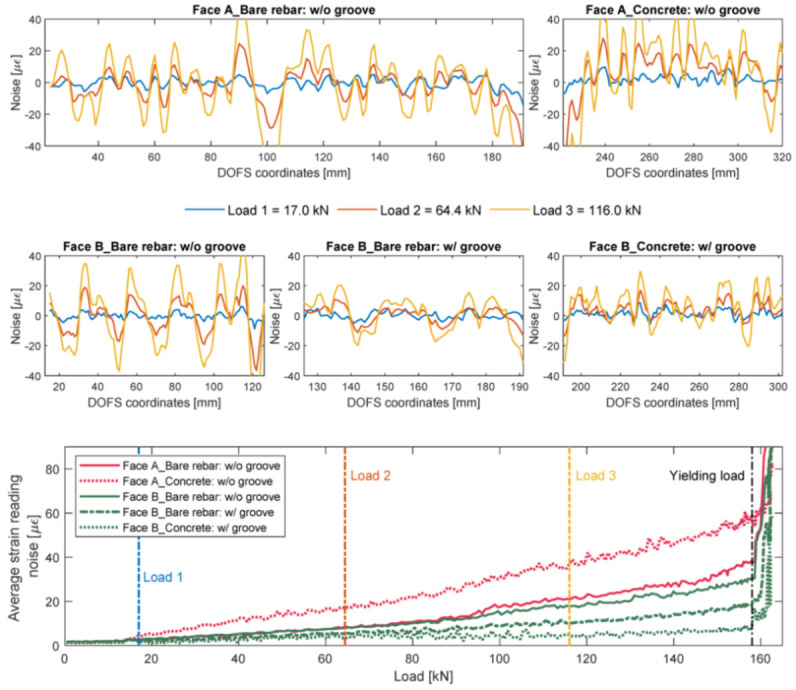
Influence of the rib pattern on the steel strain profiles during the double pull-out test of the RC tie 15 × 15 × 240 mm Ø20 (top five figures). Relative average reading noise (bottom) [49].

Additionally, as also visible in Figure 7, the oscillations of the profiles, easily confusable with signal noise, were stated to be the influence of the transversal rib on the tensile performance of the rebar. Additionally, it was observed on all the specimens that the magnitude of the defined rib-induced noise became increasingly larger (see Figure 8) with the load thus increasingly disturbing the sought-after signal, that is the strain profile.

**Figure 9:**
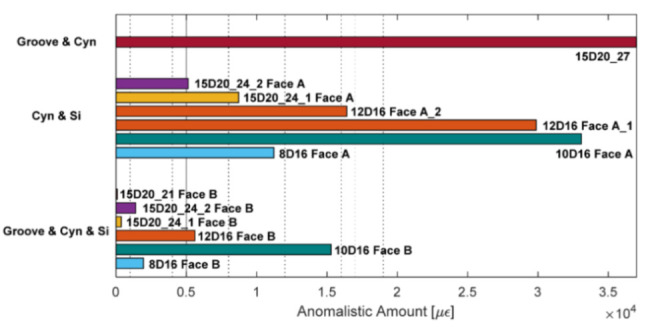
Total amount of anomalistic readings grouped per bonding technique [49].

The authors continued reporting an unexpected phenomenon, possibly connected to the under-performance of the bonding techniques, which caused an alteration of the DOFS strain readings whenever the fiber was in the presence of extra bending-induced tension (smoothing out strain profiles) and/or monitoring strains in cracked cross-sections (magnifying their amount). Additional work on the involved transfer mechanisms could be the key to such phenomena. Finally, it was asserted that, whilst none of the studied bonding techniques could completely guarantee the removal of all SRAs, the combination of a longitudinally incised groove, cyanoacrylate and silicone was the bonding technique that led to the smallest amount of SRA thus preserving the readability and reliability of the strain data (see Figure 9).

Barrias et al. [50] reported the results of a laboratory test campaign on two 150 × 150 × 600 mm RC beams whose bottom face was instrumented with four differently bonded longitudinal OFDR DOFS segments, namely with Silicone, Polyester, Epoxy and Cyanoacrylate. The beams were subjected to three-point load tests carried out under an elastic regime (uncracked condition) before being, in a second cycle, cracked and loaded to failure. The influence of the spatial resolution on the data acquisition was additionally analyzed. On the latter, the spatial resolution of 1 mm presented an undesired high spatial variability (reportedly due to the concrete’s heterogeneity of the aggregates) which did not occur with a spatial resolution of 1 cm. On the adhesive performance, in uncracked conditions, all adhesives compared fairly well with the data measured by parallelly installed SG with a slight edge from the silicone’s part whose plots were smoother and more uniform. When cracks arose, whilst all the segments were able to detect and locate the crack formation, the measurements at this location became erratic in all segments except for the silicone. Nevertheless, for the latter, the length influenced by the crack formation is relatively and suspiciously wider.

The last observation was very probably due to the aforesaid [48] silicone-induced stress redistribution.

**Figure 10:**
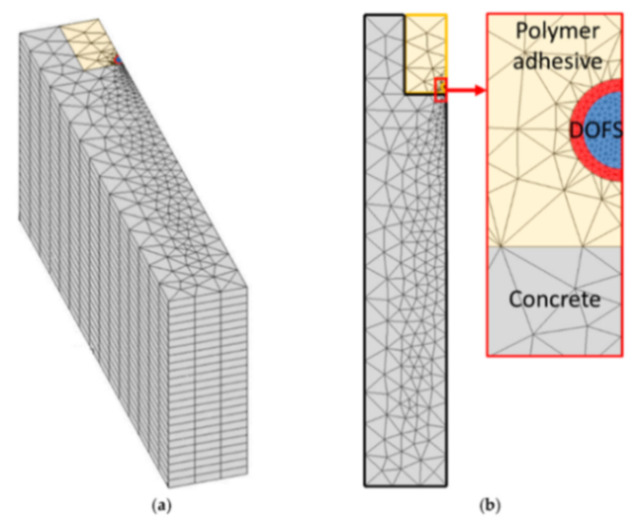
Finite element mesh of (**a**) the 3D geometry and (**b**) the cross-sectional view of the system [42].

Alj et al. [42] investigated the strain response of thick-coated DOFS sealed in a groove on the surface of a concrete structure using a polymer adhesive and aimed to identify optimal conditions for crack monitoring. Initially, a Finite Element Model was developed (see Figure 10) to understand the strain transfer process between the DOFS core and the host structure which showed that the softer the adhesive is, the lower the strain transferred to the DOFS glass core due to redistribution over a broader length of the sensor (similarly to what was observed in [48,49,50]).

**Figure 11:**
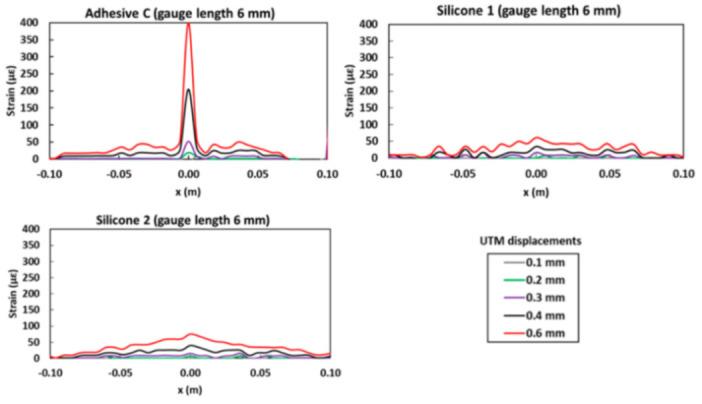
Strain profiles recorded by DOFS bonded to the concrete specimen with three soft adhesives (Bi-component epoxy, Mastic silicone and Silicone rubber) [42].

Later, mechanical tests on cylindrical concrete specimens instrumented with a polyimide coated (external diameter 160 μm) OFDR DOFS bonded/sealed using several adhesives proved that strain measurements provided by DOFS were consistent with those from conventional sensors. Furthermore, they confirmed that bonding DOFS to the concrete structure using soft adhesives allowed to mitigate the amplitude of local strain peaks induced by crack openings, which may prevent the sensor from early breakage. Unfortunately, this particular result, also corroborated by the previous articles, cuts both ways as the strain alleviation reduces the sensitivity of bonded DOFS and may alter specifically sought after strain readings. This was evident in the strain profiles provided by the DOFS (see Figure 11) which was bonded to the test specimen with three polymer adhesives used in this experiment (Bi-component epoxy, Mastic silicone and Silicone rubber) and subjected to an actuator displacement reaching 0.6 mm.

The DOFS bonded with Bi-component epoxy revealed the development of a strain peak in the central part of the profile from the actuator displacement of 0.2 mm. DOFS bonded with the two silicone adhesives, instead, did not show clear peaks development but rather small triangular shaped profiles visible only from the actuator displacement of 0.3 mm. Indeed, as already observed in [48,50], the very low stiffness of these silicone adhesives (about 5 times lower than that of the Bi-component epoxy) favored the diffusion of the originally concentrated stress over the entire length of the bonded DOFS. Consequently, the authors suggested that the adhesive stiffness should be optimized to ensure a compromise between protective function and crack monitoring performance. Alj et al. finally generalized the above FEM to describe the strain response of bonded DOFS in the presence of crack and an analytical expression relating a DOFS peak strain to the crack opening was proposed.

In Barrias et al. [51], the authors aimed at assessing OFDR DOFS’ ability and performance to monitor bridges on a long-term basis thus subjected to a large number of load cycles. The study also studied the magnitude of DOFS debonding and fatigue failure risk. To this end, two similar specimens were submitted to a fatigue test up to 2 million load cycles, the amplitude of which was representative of what is expected on a standard highway bridge under vehicular traffic. According to the authors, the first beam was fatigue loaded in an un-cracked state and its results showed a reasonably good agreement between the DOFS measurements, the theoretical results and the ones measured by the SG as well and for all adhesives tested. The second beam, instead, was intentionally pre-cracked and its results showed how DOFS continued to provide strain measurements coherent with the applied load despite some differences related to the used bonding agents. For example, the best performance was obtained for the epoxy and cyanoacrylate, whose measurements matched perfectly with those from the SG. Larger differences were, instead, observed against the other two bonding agents. Overall, though, neither fatigue failures nor fiber/adhesive debonding were detected.

Conclusively, the present section introduced several recently published results and observations on the matters of strain transfer in the multi-layered DOFS bonding systems and of the ideal bonding technology for laboratory-targeted coating-less DOFS. The main takeaways on the former were:

- Feng et al. [41] and Henault et al. [43] developed theoretical approaches to describe the strain transfer mechanism (also defined Mechanical Transfer Function) that relates the strain profile of the host cracked concrete with the DOFS core;
- Bassil et al. [44] proposed a new analytical model formulating a general expression of the crack-induced strain transfer from fractured concrete material to optical fibers;
- Alj et al. [42] indicated that the strain transfer process depended on mechanical and geometrical properties of the different intermediate layers, i.e., their modulus of elasticity, their height and the initial crack opening;
- Articles [44,52] presented particularly varied selections of DOFS cables with equally deep studies of their performances;
- Whenever an experimental investigation requires the maximum precision possible, the strain shear lag induced by any host–surface/DOFS-core intermediate layers should be minimal, thus the preferable fibers are simply cladded non-coated DOFS;
- It was experimentally assessed that simple Polyimide-cladded cables had a strain lag parameter inferior to other coating-including ones, thus allowing for accurate detection of tiny crack propagations;
- On the durability of DOFS cables embedded inside RC structures, an experimental campaign with accelerated aging of the RC saw a larger DOFS/concrete interface degradation for cables with an external coating made of a polyethylene than the ones made out of soft elastomeric material (olefin-type elastomer).

On the best bonding/protection techniques of thin coating-less fibers on RC structures:

- Numerous researchers reported the appearance of unrealistic strain readings, defined as Strain Reading Anomalies (SRAs), which prevented the extraction of reliable strain data;
- Whenever DOFS are embedded inside an RC structure, SRAs are mainly caused by the friction with concrete or (in the case of externally bonded fibers too) by steep strain gradients like the ones occurring in the proximity of surface non-linearities, such as cracks;
- Whilst it could not completely guarantee the absence of SRAs, the combination of a longitudinally incised groove, CYN and SI was reportedly the bonding technique that led to the smallest amount of SRAs;
- It was observed on DOFS instrumented RC ties that load increments increased the magnitude of the influence of the ribs on the strain profile of steel rebars;
- Several DOFS-powered experimental campaigns detected a behavioral feature of silicone whenever it was used for the protection of DOFS. That is a certain redistribution of the strains present in a specific point/section of the DOFS along a larger segment, consequently sampling lower strains than the ones originally present;
- This is in line with the results of a performed FEM analysis stating that the softer the adhesive is (in this case silicone can be characterized as a “soft” adhesive), the lower the strain transferred to the DOFS glass core due to redistribution over a broader length of the sensor;
- Following a test on DOFS-instrumented RC beams subjected to fatigue testing (2 million load cycles) the most resilient bonding adhesives were reportedly epoxy and cyanoacrylate.

## 4. Laboratory Experimental Applications

The application of DOFS for structural monitoring is still rather young, especially when compared to classic tools such as SG, LVDT and inclinometers. It can be stated that an “instrumental maturity” is still lacking when it comes to optical fiber sensing, a shortcoming that numerous research groups and projects are attempting to overcome through laboratory experimentation. Indeed, most research articles published in the latest year still motivate their experimental efforts with the study of the applicability, suitability and performance of DOFS deployments on any particular structure, under any particular testing conditions on the lookout for any particular mechanical response. Furthermore, it is common to see a comparison between DOFS’ outputs and parallelly sampled ones by means of common and well-established monitoring tools. The most common variables under investigation are the following:

- DOFS cladding, coating and reinforcements
- Adhesives or technologies used to achieve the DOFS/structure bonding and protection
- The layout of the deployment
- Positioning of the DOFS: bonded to a structure’s surface versus embedded inside it

Considering all the above variables, the solution for the best DOFS deployment is branching and permuting in nature. Consequently, over the years, multiple techniques have been proposed which will eventually “converge” in a single one in the close future, in accordance with the objective at hand. Only under such conditions, it would be possible to develop standards and guidelines for DOFS monitoring.

The present section is aimed at presenting and putting side to side the laboratory experimental efforts performed in the latest years in an attempt to help to hasten the above-mentioned “instrumental converge”.

It is worth mentioning, as will be noticeable further on, the large number of laboratory experimental campaigns that integrate DOFS monitoring with a novel technology under the name of Digital Image Correlation (DIC). Briefly, DIC is an innovative, cheap, non-contact optical technique for measuring strains and displacements on any primed surface. It works by comparing digital photographs of a component or test piece at different deformation stages and, by tracking blocks of pixels of a complex stochastic pattern painted on the monitored surface, it measures its surface displacement, building 2D and 3D deformation vector fields and strain maps [53]. As it is easily discernible, the integration of this technique inside a DOFS monitoring campaign not only does not lead to any interference but also allows to integrate, verify and validate the DOFS internally measured strains with externally measured ones. Two recent studies, Mata-Falcón et al. [54] and Bado et al. [55], explicitly analyzed this DOFS-DIC integration.

**Figure 12:**
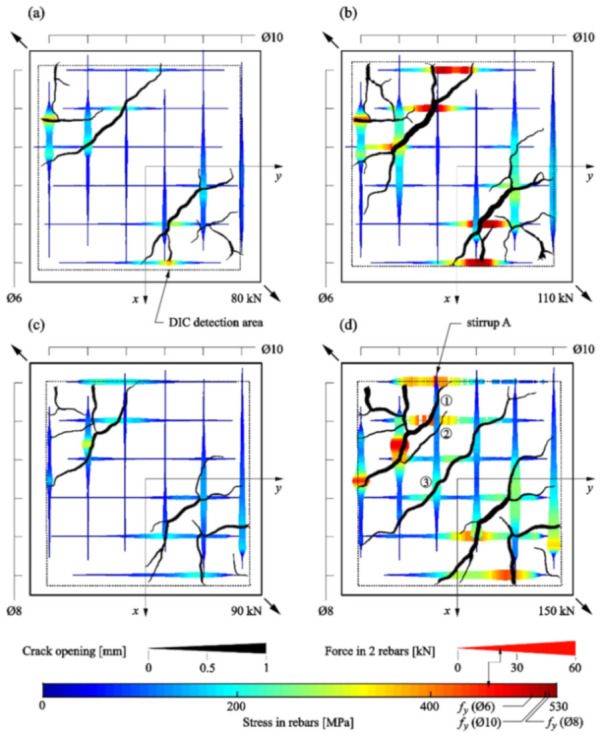
Crack locations and openings, steel stresses and forces in front layers of stirrups for Panel 1 at (**a**) 80 kN and (**b**) 110 kN as well as for Panel 2 at (**c**) 90 kN and (**d**) 150 kN [54].

The former explored the combined use of DIC and OFDR DOFS for the strain measurements of two 600 × 600 × 125 cm reinforced panels subjected to diagonal tension. The OFDR DOFS were glued with epoxy inside 1 × 1 mm grooves carved along both legs of every stirrup, summing up to 24 measuring lines across the whole specimen. As visible in Figure 12, the DOFS-sampled reinforcement strains correlated well with the position and opening of the cracks (monitored by the DIC) proving the compatibility and potential of DIC and DOFS to measure phenomena like bond, crack behavior and shear transfer.

The same conclusions were also reached in Bado et al. [55] who monitored with OFDR DOFS the strains present inside three steel rebars embedded in differently shaped and differently behaving (cracking and non-cracking) RC ties during testing. As already evident, this dual monitoring methodology is often employed for experimental works on cracking RC structures.

Therefore, with this frame of mind, the following sub-sections will explore recent research articles on laboratory experimental campaigns deploying DOFS to RC structures and more. They will be grouped in different sections exploring DOFS deployments on RC beams, RC ties, RC slabs, corrosion tests and finally on miscellaneous experimental campaigns such as fire resistance, concrete–textile hybrids and more.

Finally, additional reading on laboratory applications of DOFS can be found in Huo et al. [56], Bassil [57], and Zhang [58].

### 4.1. Reinforced Concrete Beams: Cracking and Other Serviceability Analyses

The present section collects the large research effort performed throughout the last years on DOFS distributed strain sensing for RC beams experimental campaigns.

**Figure 13:**
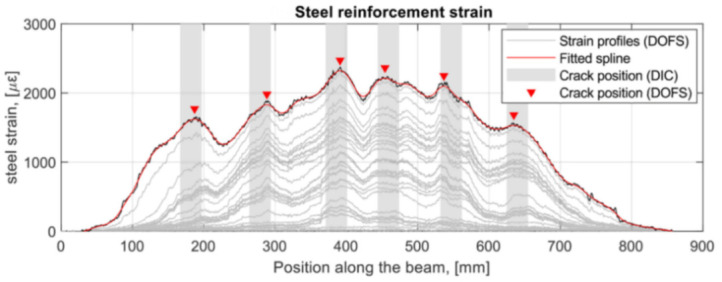
Multiple strain profiles from the DOFS where the red triangular makers indicate the determined crack position based on the strain profile at maximum load and the grey shaded area correspond to position determined from the Digital Image Correlation (DIC) [59].

Berrocal et al. [59], for example, aimed at assessing the suitability and accuracy of thin polyamide -ladded OFDR DOFS for the crack monitoring of RC beams subjected to three-point bending tests (monotonic and cyclic loading) in addition to determining the crack location and width evolution over time. The latter was achieved by means of comparison between DOFS reinforcement strain profiles and externally sampled DIC measurements. Low-bend loss polyamide-coated DOFS (155 μm diameter) were bonded directly to the rebar’s surface, along one of the longitudinal ridges, with cyanoacrylate adhesive before being protected by a silicone layer. The comparison of the crack location obtained from DOFS and DIC revealed that most of them were located within an acceptable range of ±3 cm from each other (as visible in Figure 13).

**Figure 14:**
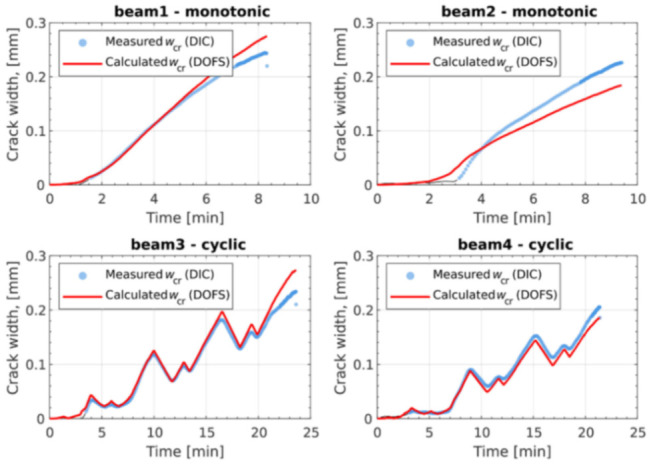
Comparison between the crack width measured by the DIC and calculated from the DOFS measurements [59].

This range is understandable considering the different depths at which the transversal cracks were measured and their probable lack of perpendicularity compared to the beams’ axis. An additional takeaway was the need for a certain amount of strain noise-removing data post-processing in order to determine the crack position. The crack width and evolution were further calculated by integration of the strain profile over a certain length adjacent to the crack and through the removal of the contribution of the tension stiffening between cracks. A good agreement (±20 μm) was generally observed between DOFS and DIC crack widths monitoring, as can be seen in Figure 14.

**Figure 15:**
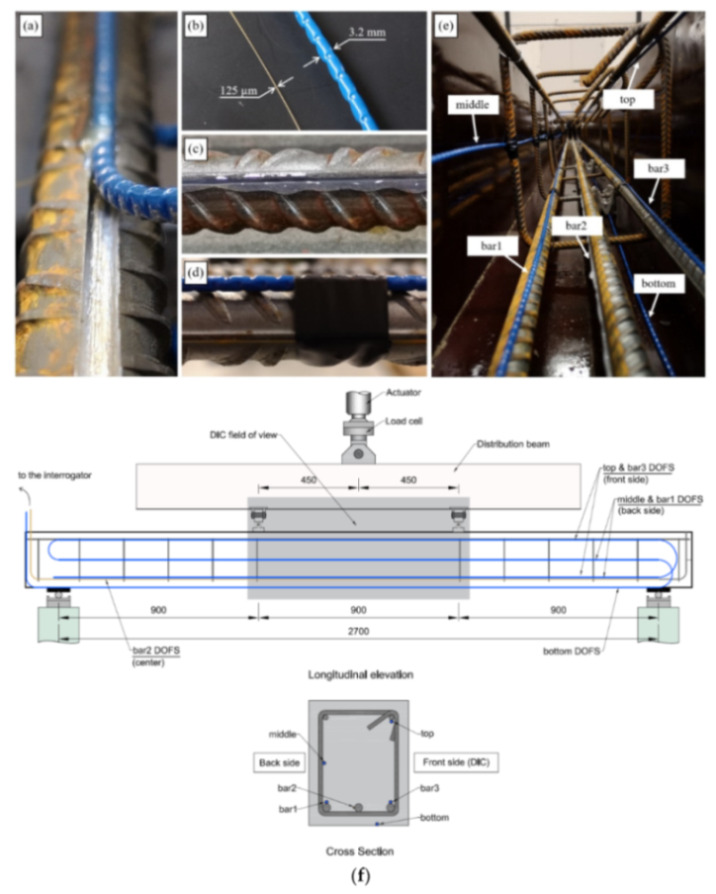
Installation of the DOFS: (**a**) installation of robust DOFS cable in a reinforcement bar by inserting it into a previously milled groove; (**b**) comparison of thin and robust DOFS; (**c**) installation of thin DOFS on the surface of a reinforcement bar by bonding it with cyanoacrylate adhesive and protecting it with silicone; (**d**) installation of robust DOFS on the surface of a reinforcement bar by mechanically anchoring the cable to the reinforcement with electric tape; (**e**) multi-layer configuration of embedded DOFS in the beam specimens and (**f**) loading setup and DOFS installation configuration for the RC beam specimens (adapted from [60]).

Berrocal et al. [60] also investigated the suitability of embedded robust OFDR DOFS (featuring a protective external rugged polyamide coating) to accurately assess the performance requirements, in terms of vertical deflection and crack width, of three 20 × 25 × 300 cm RC beams subjected to four-point bending. The strain measurements performed by means of the thicker fiber were additionally integrated with the ones extracted from thin polyamide-cladded ones and from an external DIC system. The thin DOFS were bonded directly onto the steel bars, at the concavity created by the longitudinal ridge, by means of cyanoacrylate adhesive (after removing the mill-scale and degreasing the area with acetone) with the addition of a protective layer of a one-component water-proof oxygen-free silicone rubber (Figure 15c). The robust DOFS were installed longitudinally along the rebars (Figure 15f) either supported along a longitudinal rebar, bridging the stirrups or resting on the formwork using electric tape to fix them in place (Figure 15d,e).

The results revealed that a certain strain attenuation occurred in the embedded robust DOFS compared to the commonly used thin polyamide coated DOFS. However, the presence of the protective sheath prevented the appearance of SRAs. Performance wise, the robust DOFS were able to provide a good estimate of the beam deflections with errors of between 12.3 and 6.5%. Similarly, crack widths computed on the base of the DOFS strain measurements differed by as little as ±20 µm with the DIC results.

Brault and Hoult [61] monitored simultaneously the deflections and crack widths of thirteen different RC beams tested under three-point bending. The goal was to evaluate the performance of nylon-coated OFDR DOFS cables for serviceability performance assessments and compare the latter with other monitoring technologies’, namely DIC and linear potentiometers. The DIC cameras were aimed such that their field of view encompassed the middle section of each beam’s span. The results showed that, if to use a FOS layout that avoided cracks larger than 0.15 mm and accounting for flexural deformations only, the distributed beam deflections could be accurately measured up until load levels approaching failure. Furthermore, comparing the DOFS-based crack width calculation outputs with DIC’s measurements, it was found that the FOS could measure crack widths with an average measurement difference of 0.007 mm for crack widths up to 0.15 mm, 0.016 mm for crack widths between 0.15 and 0.18 mm, and 0.031 mm for crack widths between 0.18 and 0.30 mm. The authors concluded suggesting that DOFS could be used for the monitoring of an RC structure’s compliance with the crack width limits prescribed by the codes.

Similar RC beams were also used in Brault and Hoult [62] with the objective of reporting the development of a practical technique to measure distributed reinforcement strains in RC members beyond cracking. For validation purposes, OFDR DOFS outputs were further compared against those of conventional SG. Some key takeaways were the good agreement between the DOFS and SG sampled reinforcement strains at crack locations and between the latter. The authors observed that exaggeratedly high reinforcement strains and/or noisy strain results were measured at crack locations (SRAs), likely due to concrete shearing or pinching of the DOFS. Furthermore, it was noticed that mainly unreliable strains were measured once reinforcement yielding commenced. Finally, when comparing the progression of external crack widths with the corresponding reinforcement strains at crack locations, it was detected that certain cracks initiated gradually whilst others more abruptly.

An additional DOFS-DIC integration was reported in Poldon et al. [63]. Here, a large RC beam was designed to fail in shear under three-point bending conditions with its flexural and shear reinforcement strains monitored with OFDR DOFS and its superficial strains/displacements with DIC. DOFS was bonded to the longitudinal reinforcement ribs and, notably, on 16 stirrups with a cyanoacrylate adhesive before being covered with consumer-grade silicone. According to the authors, the presented measurement technique using FOS and DIC allowed for a detailed study of shear failures and the quantification of the contribution of steel and concrete to the beam’s stiffness at every stage.

**Figure 16:**
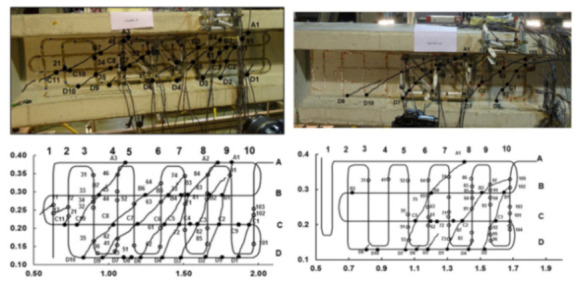
Real cracking pattern and obtained with DOFS in two different beams (adapted from [64]).

Shear failure was also analyzed in Rodriguez et al. [64,65] who investigated an experimental methodology to obtain the shear cracking pattern of RC beams. In particular, the latter suggested the formation of a 2D grid composed of one or two OFDR DOFS bonded to the shear span of a beam. The methodology was tested on three partially pre-stressed 8 m long I-shaped RC beams subjected to shear failure with a DOFS mesh bonded with epoxy adhesive. During such test the peaks appearing in the sampled DOFS strain profiles were associated with the presence of cracks (detectable even before their actual visual appearance). As the latter evolved the DOFS grid’s horizontal and vertical segments allowed to successfully define the crack pattern despite the different inclinations of the cracks along the web height (see Figure 16).

Apart from the definition of the crack pattern, an algorithm was also proposed to calculate the crack widths based on the strain profiles in both horizontal and vertical directions. On the extraction of the above shear crack widths, the proposed DOFS method was compared with the more traditional one based on the deployment of displacement rosettes in [65]. The results showed a relative similarity between the two analyses’ results despite some mismatches attributed to the high level of measurement error involved in the rosette method. Additionally, the authors observed the presence of high-strain DOFS points unrelated to the cracked visible pattern. These could be potentially attributed to small premature cracks, damages in the bonding of the fiber, presence of small cavities in the concrete surface or simple failures of the fibers’ backscattering interpretation performance.

**Figure 17:**
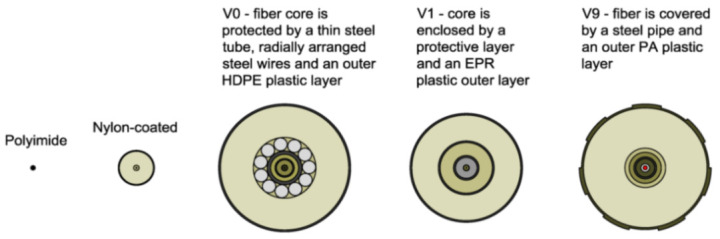
DOFS cables used in [66].

Shear failure was also studied in Fischer et al. [66] who performed a complex load test on a prestressed concrete beam subjected to four-point bending in order to investigate the various configurations of the deployed measuring device, OFDR DOFS/cables (see Figure 17), and application methods.

The authors suggested that in structural concrete construction, the robustness and durability of the monitoring DOFS (in addition to their basic suitability and sensitivity) should reflect their application (glued to the concrete surface, applied to reinforcement bars or embedded in concrete). Thus, the authors investigated both conventional polyimide and nylon coated DOFS but also more robust measuring cables (referred to as V0, V1, and V9 and N3) which include various plastic and metal layers (thus comprising comparatively thicker cross-sections). The formers were bonded to the surface of the test specimen with a cyanoacrylate-based adhesive whilst the latter were embedded inside it. During the test, all the observed flexural cracks formed peaks in the strain curve of the measuring cable. Similarly so, the formation and opening of shear cracks close to the supports could be identified by the upwardly guided sensors V9, V1, and N3 in those specific points where the DOFS and shear cracks cross. The robust measuring cables V9 and V1 were reportedly more adapt to reporting the steel strains beyond the cracking stage.

Mehdi Mirzazadeh and Green [67] studied an application of OFDR DOFS and DIC for the measurement of the crack widths, deflections, and strains in eight large-scale RC beams tested under static and fatigue loading at room temperature (+15 °C) and at low temperature (−20 to −25 °C). Half of the 200 × 400 × 4200 mm beams had no shear reinforcements whilst the remaining ones did. Single-mode nylon coated DOFS were bonded both at the top and the bottom of the beams’ side surfaces which had previously been ground and cleaned with alcohol. Additionally, in both positions two parallel rows of DOFS were deployed, one fully bonded and the other partially bonded. DOFS reportedly measured strains with an accuracy close to the one of parallelly installed SG under both temperature conditions. A noteworthy observation was that the sum of the strain-optic error (thermal stress-induced error when a coating is present) and the thermo-optic error (error caused by the alteration of the fiber refractive index due to temperature variations) was close for the two different tested fibers. The first of these was a single-mode fiber (900 μm coating diameter) whilst the second a polarization-maintaining fiber (230–260 μm coating diameter). Finally, the article introduced a methodology to determine the thermal expansion coefficient of the optical fibers with thin coatings.

**Figure 18:**
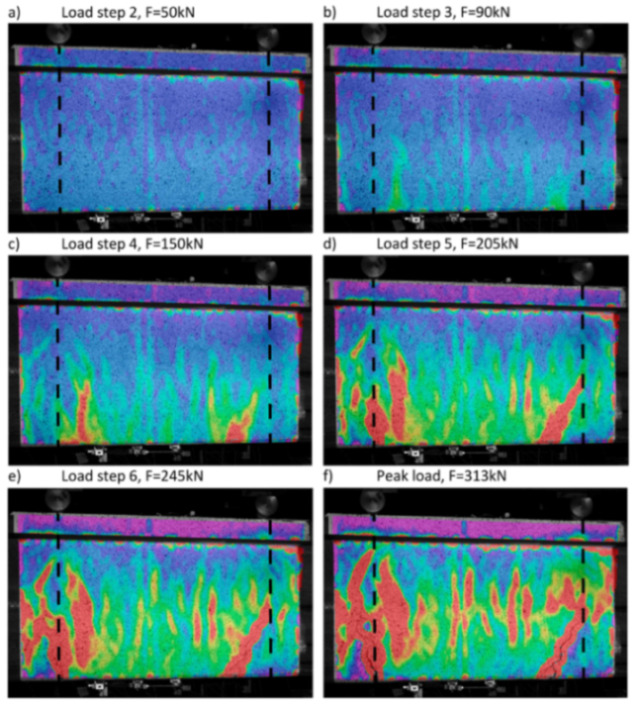
DOFS-integrating DIC strain distributions at different load steps [68].

Sawicki et al. [68] proceeded to validate the performance of DOFS (integrated with DIC and extensometers) for the SHM of Ultra-High-Performance Fiber-Reinforced Cementitious composites (UHPFRC). In particular, a full-scale UHPFRC beam was instrumented with three lines of OFDR DOFS positioned inside 2 × 2 mm grooves present on each face of the beam (the fibers were glued in place with a bi-component epoxy adhesive). The displacement-controlled four-point bending test was characterized by different loading–unloading steps. Overall, the experimental campaign yielded a successful detection of the UHPFRC beam structural discontinuities from the DOFS’ part. Yet, during the elastic stage of the beam’s structural response, large measurement noises caused the failed observation of the DIC strain variations leading the authors to state that the latter still remained a highly complex and sensitive monitoring technique. However, in the cracking stage, DIC allowed for the tracking and characterizing of the localized fictitious cracks, i.e., localized discontinuities bridged by multiple fibers (see Figure 18).

The authors further observed that polyimide-coated fibers allowed for the early and accurate detection and localization of micro-cracks. Conclusively, Sawicki et al. highlighted the importance of a deep understanding of the sensor properties (like crack and temperature sensitivity), durability and aging (tackled by [46,47]), long-term fatigue (tackled by [51]) for holistic global and local strain measurements.

**Figure 19:**
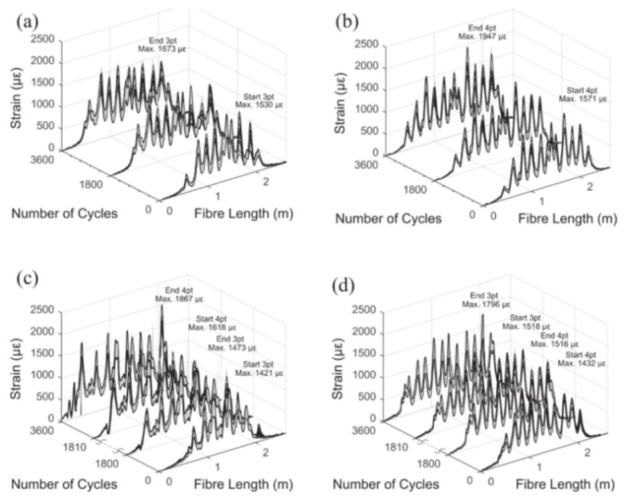
Strain along the bottom reinforcement during cycling for four deep beam specimens [69].

Broth and Hoult [69] investigated the potential of using OFDR DOFS for the monitoring of a series of slender and deep RC beams subjected to three and four-point bending cyclic loading. In particular, the embedded distributed sensors were used to assess the deflection growth induced by the cyclic loading (3600 loading cycles at 1 Hz). DOFS were bonded to sanded and degreased rebars by means of a cyanoacrylate adhesive and later protected with an additional layer of silicone. The second of the three testing phases (initial loading, cycling loading and loading to failure) saw a deflection growth (see Figure 19) attributable to the combined result of crack formation, crack growth, creep in the concrete and the degradation of the bond between the steel reinforcement and concrete.

Finally, the authors highlighted the fact that DOFS was able to withstand cyclic loadings all the way to failure.

However, at the present day, still few tests are available regarding the issues of DOFS-monitored fatigue and deterioration.

Barrias et al. [70] carried out an experimental campaign on two 100 × 180 × 800 mm beams with polyimide-coated OFDR DOFS installed both on the embedded rebars and on the specimen surfaces. The beams were tested under three-point bending starting with an initial monotonic loading cycle, followed by an unloading stage (triggered well beyond the cracking stage) and concluded by another loading cycle. In order to verify the reliability and accuracy of the DOFS measurements, additional SG were installed along the rebar in three locations. Overall, the optic fiber bonded to the rebar delivered good results despite disturbances in the surroundings of the crack. The latter was also reportedly detected and located by the superficial DOFS albeit only until a certain stiffness variation in the beam, beyond which large disruptive SRAs appeared in the proximity of the damage location.

**Figure 20:**
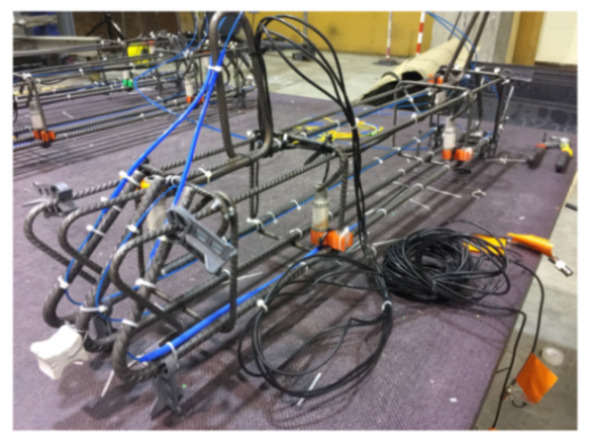
Ultra Sonics (US) and DOFS sensors attached to the rebars before casting of concrete [71].

Bassil et al. [71] proceeded to the performance assessment of Coda Wave Interferometry and OFDR DOFS (deployment visible in Figure 20) for the damage detection of a 20 × 20 × 100 cm RC beam tested under three-point bending.

The beam was also instrumented with SG and Linear Variable Displacement Transformers (LVDT). The DOFS was glued to the beam’s front and back surfaces, to the front bottom rebar, mid top rebar, mid bottom rebar and back bottom rebar. The results showed that the implementation of Coda Wave Interferometry and DOFS allowed for the detection of early subtle changes in RC structures until crack formation. On DOFS, the authors concluded that, beyond the latter stage, their ability to quantify the crack openings permitted effective monitoring of RC structures.

Wu et al. [72] tested an RC beam under four-point loading with OFDR DOFS both glued to its surface and embedded inside it on three separate layers. Experimental results indicated that cracks could be detected as sharp peaks of the measured strain distribution profiles, despite also noting that strains pertaining to neighboring cracks could interfere with each other. Indeed, the authors pointed out that only neighboring cracks with a separation of more than 6 cm could be discerned with a 1 cm spatial resolution. Finally, both the widening and the propagation of cracks were successfully detected and described to have a slow initial growth followed by a linear growth stage.

Chen et al. [73] brought up the condition under which an RC structure might be subjected to bi-directional loads. They then proceeded to use a (Pulse-Pre Pump) PPP-BOTDA DOFS (with a 10 cm spatial resolution) to assess the superficial structural strains of a 10 × 15 × 150 cm RC beam subjected to bi-directional loading. The strain–deflection relationship was established using the explicit function analysis method and the implicit function mapping method based on the DOFS readings.

Malek et al. [74] utilized OFDR DOFS for the study of the steel/concrete bond degradation due to reinforcement yielding in RC beams subjected to lateral loading. The performed experimental program, representing a drastic departure from the common bond/slip determination tests (i.e., the single and double pull-out tensile tests), was based on a pseudo-static pushover test of four 25 × 35 × 285 cm cantilever RC beams tested vertically. Instrumentation-wise, two different types of optical fibers were used, a PVC-coated tight buffer OFS cable (for both internal and external sensing) and a polyimide-coated fiber (bonded on the longitudinal rebars). Thus, during the test, the strains were captured continuously at a spacing resolution of 2.6 mm and with an accuracy of 25 με both on the outside and on the inside of the specimen. This allowed for the determination of the concrete/steel slip, steel stress, bond stress, bond deterioration length and the locations of cracks. Finally, the authors introduced a novel model to predict pre- and post-yield bond behavior.

Sounthararajah et al. [75] presented an experimental campaign aimed at evaluating the OFDR DOFS reliability and accuracy when measuring the flexural strains of a Cemented Pavement Material (CPM) beam. In particular, both static and dynamic four-point bending tests were performed on DOFS and LVDT-instrumented 50 × 50 × 200 mm CPM beams. The latter were manufactured with Holcim Road Base pavement material from the Gosnells Quarry, Perth, Western Australia combined with a General Purpose cement as a binder substance. An acrylate-coated single-mode fiber (SMF-28e) was bonded to the bottom and side surfaces of the beam specimens (with a two-part epoxy adhesive) after having been properly cleaned with acetone. The experimental output showed a fair agreement between DOFS measured and calculated flexural strains suggesting their performance for this scope despite its taxing surface preparation. According to the authors, the test substantiated DOFS’ capability of detecting the crack initiation and propagation in CPM beams.

### 4.2. Reinforced Concrete Tensile Elements: Steel/Concrete Bond Stresses, Cracking and Concrete Shrinkage

RC ties are often used to illustrate cracking, deformation and bond behavior of RC structures thanks to their simplicity and a reasonably good representation of the distribution of internal forces and strains in the tensile zone of RC structures [76]. As such, a certain number of research teams have attempted to upgrade the common RC tie test with the embedment of DOFS (bonded to the reinforcement bars).

**Figure 21:**
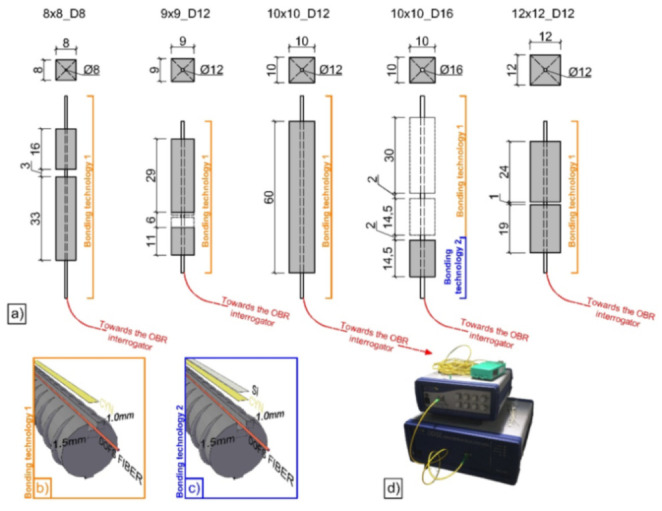
(**a**) Tested RC ties’ with their respective bonding technologies, with (**c**) and without (**b**) the addition of a protective silicone layer. The DOFS-instrumented rebars were monitored by means of an (**d**) ODiSI-A OBR interrogator [77].

Bado et al. [77], for example, performed a double pullout test on five differently sized RC ties (with OFDR DOFS bonded to their rebars in different manners as visible in Figure 21) with the goal of studying the steel/concrete interaction, i.e., bond and slip.

According to the authors, before the advent of DOFS, due to the lack of tools able to assess the rebar strains in an accurate, completely-distributed and un-intrusive fashion, most structural analyses relying on such knowledge (such as tension stiffening) resorted to theoretical, empirical or numerical solutions. They continued suggesting that the introduction of DOFS allowed to finally acquire that insight. In the reported experiment, the RC ties’ crack positions detected by DOFS were compared against the ones detected by a simultaneous DIC monitoring. Briefly, thanks to DOFS, the authors detected some specimen’s hybrid bond/slip behavior falling between a crack formation stage’s and stabilized cracking stage’s whenever they were characterized by a length dangerously close to surpassing the maximum theoretical crack spacing length. The latter was translated into different bond-slip profiles. The authors concluded by noting that for future DOFS applications a combination of gluing adhesive (cyanoacrylate for example) with the addition of a protective layer (silicone) was the preferable DOFS/rebar bonding technique as it removed undesired SRA-related uncertainties.

**Figure 22:**
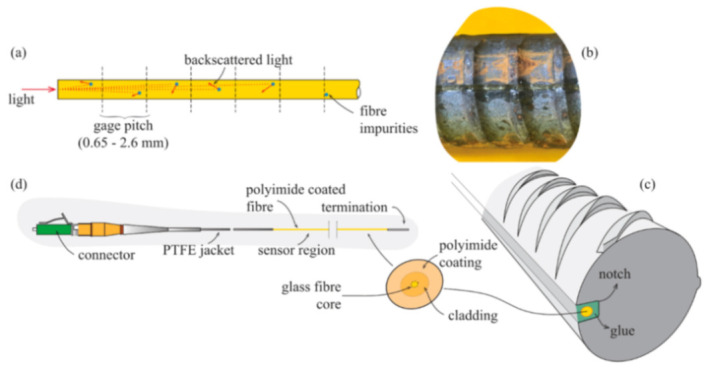
(**a**) Back-scattering mechanism due to fiber core impurities, (**b**) view of the fiber-optic sensor glued to a steel rebar, (**c**) sketch of the fiber optical cable placed in the groove and adhering to the steel due to two-component glue and (**d**) description of the fiber optic cable structure with angle polished connector on one side and termination (which avoids light reflection) on the opposite [78].

Cantone et al. [78] also provided a comprehensive work on the determination of the rebar–concrete interaction by means of OFDR DOFS and DIC monitored RC structures tested under pullout, double pullout and bending conditions. Similar to the previous article, the authors also positioned the DOFS inside 1 mm-depth grooves performed along two or four rebar sides (see Figure 22).

**Figure 23:**
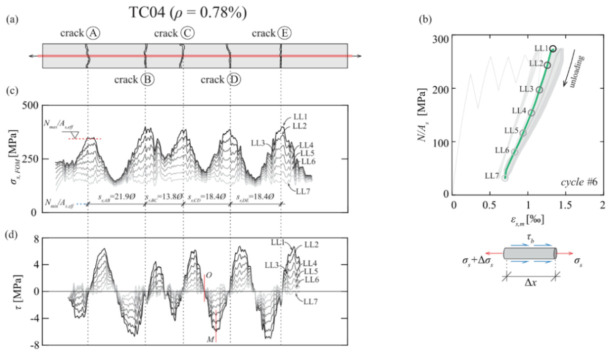
Tensile tests—specimen TC04: (**a**) cracking pattern in the cyclic loading phase, (**b**) stress-strain relationship for all cycles (in grey) and cycle #6 (in green) indicating load levels, (**c**) profiles of stresses, calculated on the basis of the measured DOFS (here defined as FOM) strains and (**d**) bond stresses τ profiles along the steel rebar (adapted from [78]).

Amongst the experimental campaign conclusions, the followings are particularly relevant. The observed strain gradients and high strain values sampled during the pullout test confirmed that this test methodology could prove to be unsuitable to characterize the response of embedded reinforcement. During the multi-cycle double pullout tests, by means of DOFS, the authors were successfully able to track the increasing bond degradation (see Figure 23) providing results in sound agreement with the literature. Finally, during the testing of DOFS instrumented RC beams, lower bending-induced stress variations were detected in the longitudinal reinforcement with respect to the theoretical values calculated with elastic-cracked analyses. This was reportedly due to imperfect closure of cracks and negative tension-stiffening effects.

**Figure 24:**
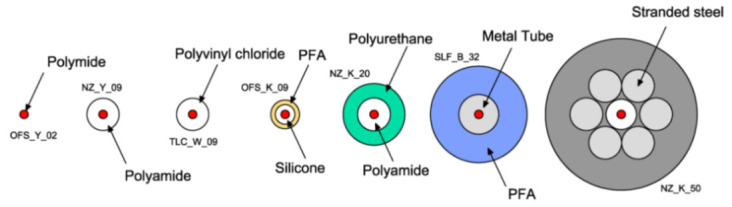
DOFS cables used in [79].

Zhang et al. [79] investigated the ability of DOFS to detect concrete cracking and large strain steel deformation of six 120 × 120 × 1000 mm RC ties with different OFDR DOFS cable structures, coating materials and diameters (see Figure 24) which were both embedded in the concrete and bonded to the rebar. The article reported how OFDR successfully detected continuous rebar deformations and the evolution of the concrete–steel bond stress up until and beyond the yielding range as well as identifying the crack-indicative strains.

Similarly to [77], the authors highlighted the potential of DOFS combined with double pullout tests for the evaluation of existing RC structure behavioral predictive models through the bond stress analysis. Worth noting are the conclusions on the performance of each DOFS cable, whose different composition was shown to have a substantial influence on the measured strain response especially beyond the onset of nonlinear behavior and into large deformation regimes. Generally speaking, the measurement sensitivity parameter decreased with an increase in the cable coating thickness due to both elastic and inelastic coating deformations. The sensitivity also tended to decrease with increased loading likely due to debonding between the cable and concrete (with the exception of cable NZ_Y_09). Finally, a certain influence, particularly on the bond with concrete, was reported to be exercised by the coating stiffness and the cables’ surface properties. Another key requirement was reportedly the fiber survivability, both in terms of measurement survivability (i.e., the deformation limit before which data can still be obtained) and the cable survivability (i.e., limit at which the fiber is permanently damaged). On the latter, the authors pointed out that a high sensitivity did not necessarily correlate with low survivability. For example, cable NZ_Y_09 reportedly boasted twice the sensitivity compared with NZ_K_20 with 50% data loss occurring at comparable crack widths.

Sienko et al. [80] presented the outcome of a laboratory test designed to verify the suitability of a standard “tight jacket” OFDR DOFS for the strain analysis of RC members. In particular, the authors deployed the optical fiber cable inside a 77 × 110 × 1050 mm RC tie along several longitudinally drilled holes before bonding it with epoxy resin. The same adhesive was used to bond the fiber to the degreased reinforcement bar parallelly to its longitudinal rib. According to the authors, the results showed good measurement accuracy for micro-cracks and narrow crack widths (range up to 0.05 mm) and only moderate accuracy in the case of wider cracks (up to 0.30 mm). This was attributed to the slip between the DOFS core and its external jacket leading to fiber strains not corresponding fully with the actual deformation state of the concrete member. However, it was also reported that the measurement methodology allowed for precise crack widths determination as to the sum of fiber elongations within the sections between the cracks.

Davis et al. [81] instrumented multiple reinforcement bars with OFDR DOFS before embedding them inside RC prisms, thus forming RC ties. Their goal, measuring the steel rebars’ distributed strain profiles during the concrete shrinkage period of time (3, 7, 21 and 28 days) and during subsequent axial tension tests. Davis et al. instrumented the bars with a polyimide-cladded and a nylon-cladded DOFS bonded to the bar with a cyanoacrylate along the longitudinal ridge. It was observed that the shrinkage at both specimens’ free edges was greater than at its center with only small strain variations (± 20 με) between these two. Furthermore, with the passing of the days, the DOFS bonded to the rebar and embedded in the concrete displayed progressively increasing compressive strains (approximately 300 με at 28 days).

A proper shrinkage strain measurement is imperative for a proper assessment of the tension stiffening as it may lead to an apparent underestimation [82]. To remove such effect, the authors used some guidelines provided in Fields and Bischoff [83] and found a good agreement with the DOFS experimental results for the rebar 15 M specimens and not so good agreement with the rebar 10 M specimens. In conclusion, two additional observations are worthy of emphasis. First, the authors’ suggestion of bonding DOFS on both sides of the reinforcement bars in order to mitigate the localized bending effects due to non-straight bars and non-symmetric crack formations. Second, the observation of a certain amount of slip between nylon claddings and the sensing silica cores could result in incorrect measurements.

### 4.3. Reinforced Concrete Slabs

The present sub-section starts acknowledging 2013’s pioneering work by Villalba and Casas [84] which represented one of the first structural applications of DOFS monitoring. In particular, the publication was based on the OFDR DOFS instrumenting of an RC slab with successful detection of both crack locations and widths as per a subsequent publication by Rodríguez et al. [85].

**Figure 25:**
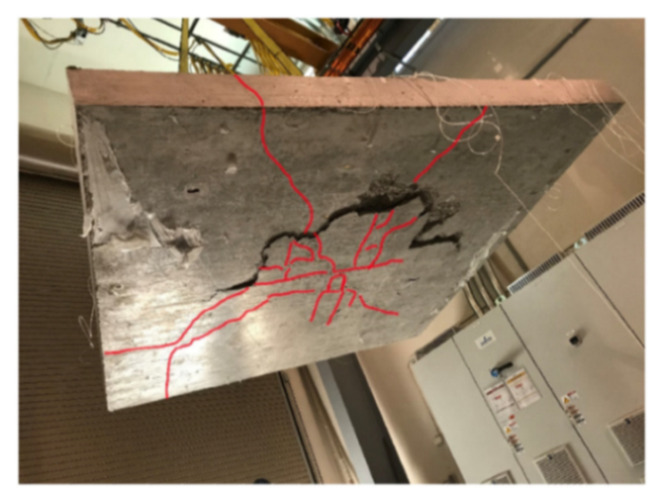
Specimen failure due to punching shear and its crack pattern [86].

More recently, in Nurmi et al. [86], a proof of concept study was undertaken that involved the measurement through OFDR DOFS distributed strain profiles in two-way RC slabs with varying reinforcement ratios and levels of axial restraint tested under a central point load. The goal, shedding light on the impact of partial restraint and reinforcement ratio on two-way slab behavior and comparing the load capacity and strain assumptions to a yield-line analysis. Two slabs were subjected to axial restraint using a welded HSS restraint frame whilst two others were not restrained. The slabs with less reinforcement demonstrated a ductile flexural failure while the more heavily reinforced slabs failed due to punching shear (see Figure 25).

**Figure 26:**
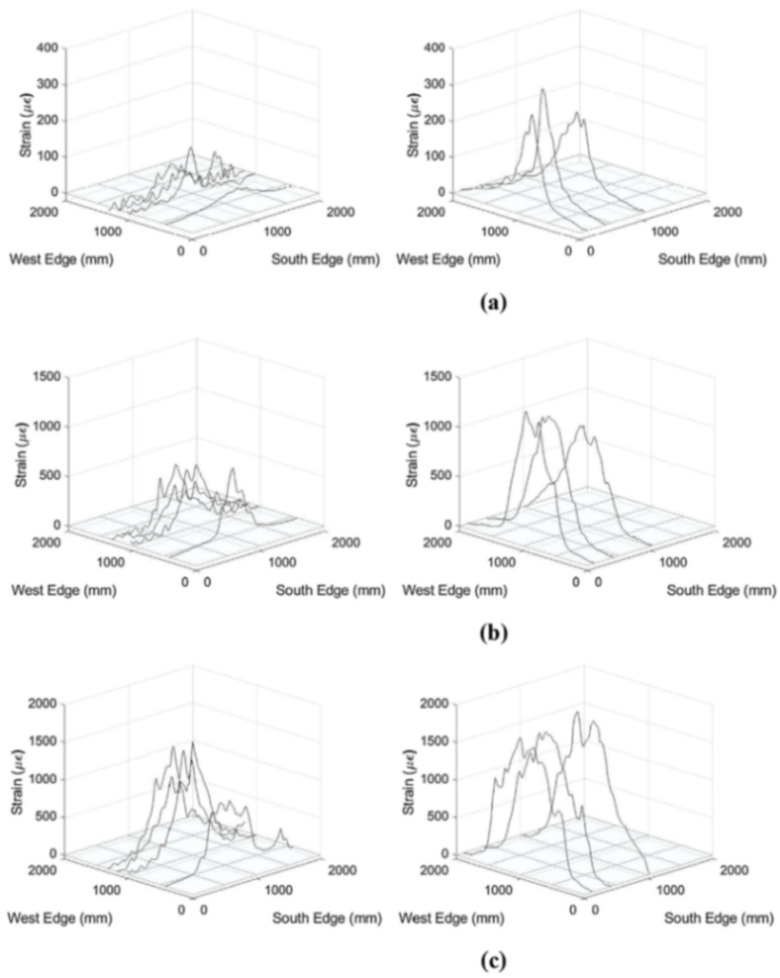
Slab 3-C-45 DOFS strains: (**a**) 50 kN, (**b**) 100 kN, (**c**) 150 kN (adapted from [86]).

The DOFS sampled strain data (Figure 26) enabled the successful detection of performance differences for the two support conditions (vertical and axial) and the capturing of the onset of reinforcement yielding. The authors additionally observed an enhanced slab capacity and stiffness in the presence of axial restraint.

Sienko et al. [87] glued OFDR DOFS cables on the composite rods of a truck scale platform slab in order to analyze the internal concrete strains and local nonlinearities (cracks). The monitoring was ongoing throughout the concrete hydration process (thermal-shrinkage strains), the prestressing tendons activation (strains regarding the transfer of compression forces from the tendons to the concrete) and the laboratory tests, when slabs were mechanically loaded until failure. The reported early-age hydration-induced concrete strains reached the value of ±220 με. Later, during the first test steps, the platform worked linearly and elastically (deflection of 10 mm) whilst later on DOFS reported the presence of discontinuities related to the crack occurrence which continued developing during subsequent load steps.

**Figure 27:**
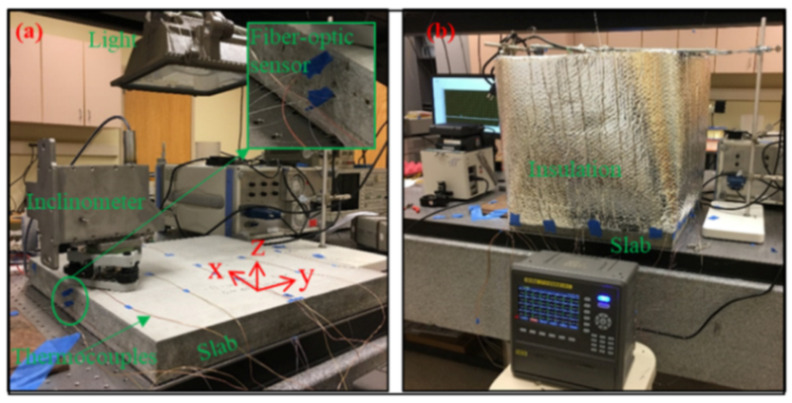
Test setup: (**a**) instrumented test slab, (**b**) testing with surrounding insulation [88].

Liao et al. [88] instrumented an RC slab with OFDR DOFS in an attempt to monitor its thermal curling process when subjected to external temperature fluctuations. Explicitly, a 50 × 50 × 5 cm mortar slab was instrumented with the combined monitoring system consisting of inclinometer, DOFS, and thermocouples. During the testing, the heat source was a light heater with a 500 W halogen bulb positioned above the slab except that the heating was not direct but buffered by double polymer insulation (see Figure 27) positioned in order to create a relatively even temperature field above the slab.

According to the authors, DOFS demonstrated that the top surface of the slab had a fast reaction rate to the heating/cooling process whilst the bottom one suffered a time-lag resulting in a top-vs-bottom temperature difference. In conclusion, the authors stated that DOFS can provide a comprehensive picture of the longitudinal temperature distributions, the heat flux transfer process and the consequent strain distributions thus returning an immediate idea of the deformed shapes of the pavement slab.

**Figure 28:**
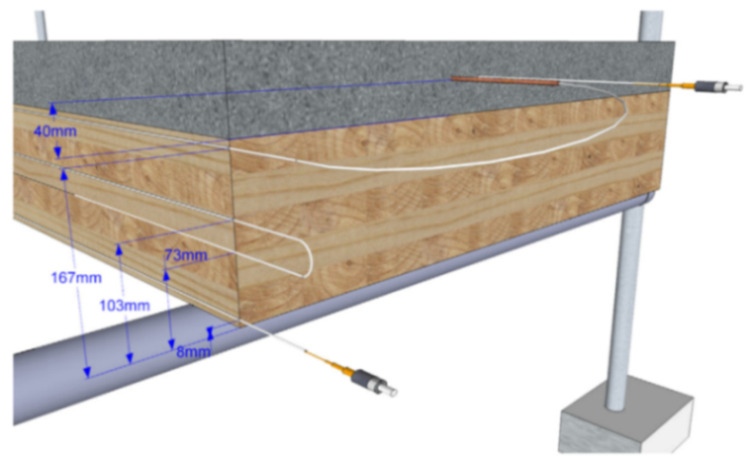
Fiber arrangement for the Timber–Concrete Composite (TCC) slab monitoring [89].

Teguedy et al. [89] sustained that the study of the early-age behavior of Timber–Concrete Composite (TCC) structures is of great interest as it provides valuable information for manufacturing specification development, quality control, and optimization of the formwork design. As such, the authors decided to deploy BOTDA DOFS inside two 900 × 75 × 8500 mm TCC slabs in order to continuously monitor (during 30 days) their short-term behavior, i.e., the early-age temperature/strain variation in the fresh concrete and in the CLT slab. Additionally, the multiplexing capabilities of the DOFS system allowed for the strain measurement at several heights of the timber section with a single sensor (see Figure 28).

The above illustrated distributed sensing system allowed for the detection of the changes of the composite action between timber and concrete (using indicators such as the neutral axis position and the curvature evolution within the CLT slab) in addition to highlighting the considerable structural changes induced by mechanisms such as concrete creep and environmental thermos-hygrometric variations.

### 4.4. Corrosion Tests of Reinforced Concrete Structures

The corrosion of steel reinforcement bars decreases the load-carrying capacity of RC structures by reducing the reinforcement area and decreasing the latter’s bond with the surrounding concrete. With regards to this deterioration phenomenon, DOFS has the potential of becoming a crucial SHM tool thanks to their immunity to corrosion and the potential of providing an insight into the internal corrosion levels, areas of pitting corrosion, crack locations and bond deterioration [90].

**Figure 29:**
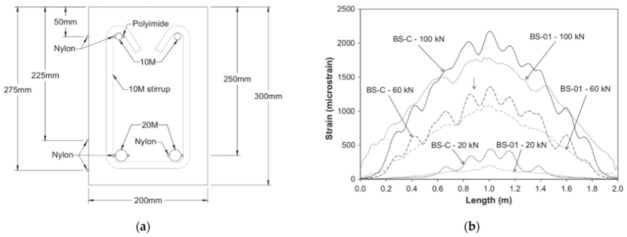
(**a**) Specimen’s cross-sectional layout and (**b**) comparison of the nylon bottom DOFS measured strains between a non-corroded control beam (BS-C) versus a corroded one (BS-01) for three applied load steps, 20 kN, 60 kN and 100 kN (adapted from [90]).

Davis et al. [90], building upon their previous work [91] on corroded RC ties, investigated the use of OFDR DOFS for the detection of corrosion in RC beams. In particular, six RC beams were instrumented with nylon and polyimide coated fibers installed on both the bottom 20 M bars and on the top 10 M bars (see Figure 29) with a cyanoacrylate adhesive.

The beams were subjected to varying levels of corrosion by placing them in a bath of water with 3.5% of the weight NaCl and imposing a current to accelerate the oxidation process of the reinforcement. The beams were finally tested under three-point bending. The authors’ conclusions were preceded by a warning on the possible exacerbation of the corrosion effects induced by the test setup when compared to in-situ corrosion. Overall, the FOS monitoring system was reportedly able to detect the strain distribution changes along the longitudinal reinforcement due to the corrosion-induced loss of bond between the concrete and reinforcement. Furthermore, it was found that 1.1% and 4.0% corrosion levels of the bottom longitudinal reinforcement with short watertight protective sleeves at its ends could cause a decrease in ultimate load of 13% and 39%, respectively. For the beams with long protective sleeves, instead, 1.4% and 3.8% corrosion lead to a decrease in ultimate capacity by 38% and 37%, respectively.

Lv et al. [92] attempted to evaluate the corrosion-induced damage degree on RC structures by using BOTDA DOFS to monitor the expansion strains of the former. The authors deployed the DOFS on the rebar in a particular way, baptized in its complexity as “corrosion sensor”. Its production steps were as follows. First of all, a 5 mm thick mortar layer was poured directly on the rebar and left curing for one day before demolding it. Three days later a 3 m long DOFS was tightly winded for several turns against the surface of the mortar with both its ends fixed with acrylate adhesive. Finally, after having bilaterally led out the fiber, an additional layer of mortar was cast on top of the latter, embedding it. Two RC 140 × 140 × 180 mm specimens (one with stirrups and one without) were subjected to accelerated corrosion and a subsequent finite element analysis was performed. The latter’s results were consistent with the fiber monitored strains proving the “rationality” of the analysis and confirming that the so-called “DOFS corrosion sensor” could be used to monitor the expansion strain of the steel corrosion in real-time.

**Figure 30:**
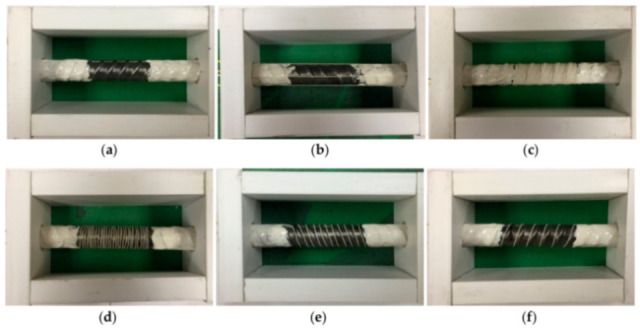
Photograph of the DOFS deployment on the steel bars: (**a**) Reference bar (**b**) L4 longitudinal deployment, (**c**) S0 spacing 0 mm, (**d**) S2 spacing 2 mm, (**e**) S5 spacing 5 mm and (**f**) S10 spacing 10 mm [93].

Fan et al. [93] investigated PPP-BOTDA DOFS’ ability to monitor the evolution of the corrosion of steel bars embedded inside concrete with the sensors deployed both along the rebar and winding around it (with spacings: 0 mm, 2 mm, 5 mm and 10 mm) as visible in Figure 30.

In the first place, the authors studied the potential influence of the sensing cable on the steel–concrete bond strength through several pull-out tests. It was noticed that whilst no bond alterations were observed between a fiber-less reference specimen and longitudinally bonded fiber specimen, the spirally bonded one led to a certain bond reduction (for example, as the spacing is reduced from 10 mm to 5 mm, the bond strength was reduced by 18%). Following such, electrochemical testing was carried out to assess the influence of the fiber installation method on the corrosion resistance of the RC specimen. In particular, the measured strain distributions were used to quantify the corrosion layer thickness and to estimate the corroded volume.

The same team published another research article [94] on the PPP-BOTDA DOFS monitoring of the corrosion evolution of a rebar embedded inside concrete with different percentages of steel fiber reinforcement and different depths of a superficially incised notch. The rebars were instrumented as specimen S10 in the previously illustrated campaign. The resulting DOFS measurements allowed the authors to conclude that under identical surface conditions (notch depth) the steel fibers reduced the corrosion of the steel bar embedded in the concrete and delayed any corrosion-induced concrete cracking (provoked by the pressure of the corrosion products against the concrete matrix). Indeed, when the steel fiber content increased from 0% to 1%, the strain increase rate before concrete cracking was reduced by 49%, 50%, and 44% for the specimens with notch depths of 20 mm, 10 mm and 0 mm, respectively.

**Figure 31:**
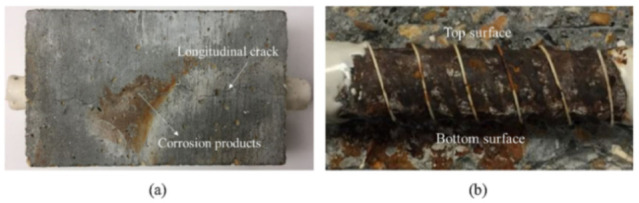
(**a**) Photography of the tested specimen and (**b**) cut section along the steel corroded bar [23].

Additional similar work from Fan et al. [23] was aimed at evaluating through PPP-BOTDA DOFS the corrosion products volume and mass loss of steel bars in addition to predicting the cracking of the concrete cover for RC specimens. The latter are similar to the ones in the previous articles (see Figure 31) with the exception of being characterized by the different cover thickness (28 mm, 35 mm, and 43 mm) and water-cement ratio (0.4, 0.5, and 0.6).

According to the authors, the DOFS results showed that the steel bar’s corrosion process occurred in three stages at each of which the strains increased approximately linearly but at increasing rates. The thickness of the concrete cover reportedly influenced the duration of each of the three stages. Meanwhile, the increase of the water-to-cement ratio led to a shortening of the first stage and an increased volume of rust filling the pores near the interface between steel and the concrete.

### 4.5. Miscellaneous

The present subsection is intended to collect experimental campaigns that, despite carrying significant weight in each relative field, are characterized by more diverse traits when compared to the above reported DOFS-powered monitoring campaigns. Therefore, the following assortment presents a high level of heterogeneity.

**Figure 32:**
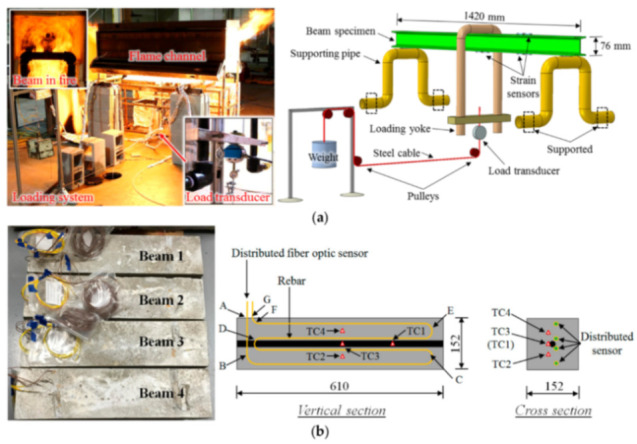
Applications of DOFS in structural fire testing of: (**a**) Steel beam and (**b**) RC beams [96].

Bao et al. [95] discussed the importance of research on the integrity and load-carrying capacity degradation when a structure is exposed to fire. In particular, as summarized in a comprehensive review article on the use of fiber optic sensors for Structural Fire Engineering [96], the team published in 2017 two experimental works featuring PPP-BOTDA monitoring campaigns on the residual performances of both steel and RC structures during a fire. In the first [97], DOFS were installed along the top and bottom flanges of a hot-rolled S-shaped A36 mild steel beam which was later exposed to fire. In particular, the fibers were loosely passed through steel tubes (which in turn were attached to the steel beam) for the measurement of the temperature distributions. A yoke was attached at the mid-span of the beam and was loaded through a pulley system (see Figure 32a).

The resulting DOFS measurements, combined with the measured temperatures and the building code recommended material parameters, yielded an enhanced thermomechanical analysis of simply supported steel beams subjected to the combined action of thermal and mechanical loading. The simulated strains and deflections were then validated using measurements from a second distributed DOFS strain sensor and two linear potentiometers resulting in accurate predictions of the temperature-dependent material properties. Of the four investigated strain predicting building codes, the European building code provided the best predictions.

In Bao et al. [95], instead, PPP-BOTDA DOFS were embedded inside RC beams (see Figure 32b) for condition assessment during the exposition to fire. Here, the distributed sensor measured temperature distributions with a 2 cm spatial resolution in the beams thus detecting non-uniform temperature distributions, concrete cracks and spalling until complete failure occurring after excessive concrete spalling.

**Figure 33:**
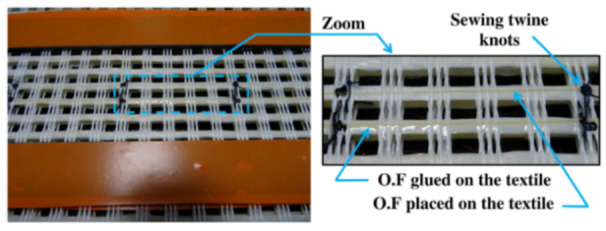
Positioning of the optical fiber on the textile reinforcement [98].

Saidi and Gabor [98] presented an experimental study on the mechanical behavior of Textile Reinforced Cementitious Matrix Composites subjected to direct tensile loading. The specimens were instrumented with OFDR DOFS positioned in their core (see in Figure 33) and for the study of the local and global behavior of the matrix, of the textile and of the textile/matrix interface.

Jaafari et al. [99] stated that early-age shrinkage, creep and thermal conditions or initial cracking could have a significant impact on the dynamic behavior of RC structures. In order to quantify the impact of early-age drying shrinkage on the latter’s dynamic behavior, the authors conducted an experimental campaign on two types of RC portal frames subjected to pseudo-dynamic testing. The first frame was kept in endogenous conditions (it was covered with plastic sheets at its early age to prevent any water exchange with the surrounding environment) thus limiting the drying effects. The second one, instead, was kept in non-endogenous conditions (thus allowing all water exchanges) similarly to any construction site conditions. The structures were both instrumented with Mono-mode optical OFDR DOFS characterized by a silica glass core covered by a Polyurethane cladding. The distributed sensors reported that, whilst the maximum strains were similar in both structures, the gradient of deformation was more important in the endogenous portal frame than in the non-endogenous portal frame. As a matter of fact, in the former one evident drying cracks appeared. After their early age period, both RC portal frames were subjected to an equivalent seismic loading and certain behavioral differences between the two were observable when subjected to a moderate intensity earthquake.

**Figure 34:**
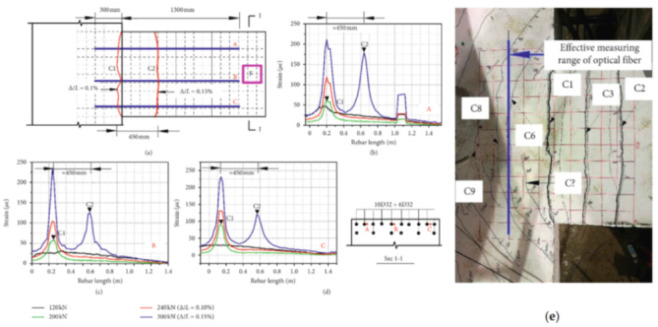
(**a**) Damage status at 0.15% drift, (**b**–**d**) are the strain profiles measured in the top fibers A, B and C at drift levels below 0.15% and (**e**) picture of the cracked specimen (adapted from [100]).

Liu et al. [100] performed an OFDR DOFS monitoring of an RC wall-beam-strut joint subjected to a monotonic lateral load. The optical fiber sensor used in this study is a polyurethane elastomer-coated fiber with a diameter of 0.9 mm without any special protective coating in the sensing cable. The sensors were glued to a groove incised on the steel bar and were positioned on different vertical and horizontal layers of the RC specimen. During the test, the authors reported an excellent agreement between the visually observable cracks against the measured tensile strain profiles. According to the authors, the initiation of cracking and its location can be identified by local spikes in the tensile strain profile (see Figure 34). This process, though, was mentioned to be much easier at lower load levels than at higher ones due to the overlapping of crack-induced spikes and the rise of SRAs occurring at higher loads. The extraction of data on crack orientation and depth was possible thanks to the multiplicity of layers on which the DOFS was bonded. The authors concluded evidencing the ability of the optical fibers inside RC members to capture its cracking pattern.

**Figure 35:**
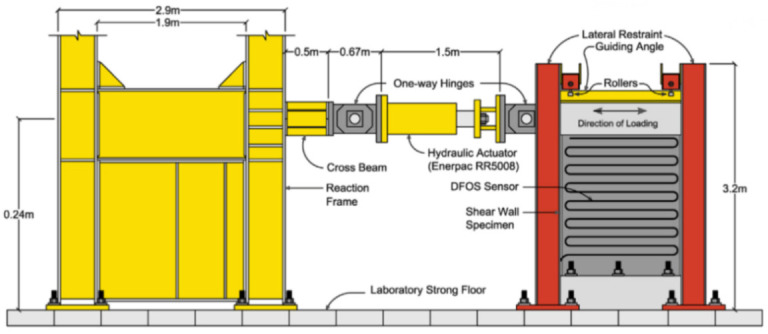
Experimental test setup (adapted from [101]).

Woods et al. [101] tested an RC shear wall with externally-bonded OFDR DOFS-instrumented Fiber Reinforced Polymer (FRP) sheets placed to improve its performance. In order to simulate the lateral drift demand along with the kind of damage suffered by an RC shear wall during a large earthquake event, the specimen was tested under a predetermined reverse cyclic lateral load sequence. This was achieved by fixing the tested wall to the laboratory strong floor and having a hydraulic actuator (fixed on a reaction frame as visible in Figure 35) apply lateral loads to its top.

**Figure 36:**
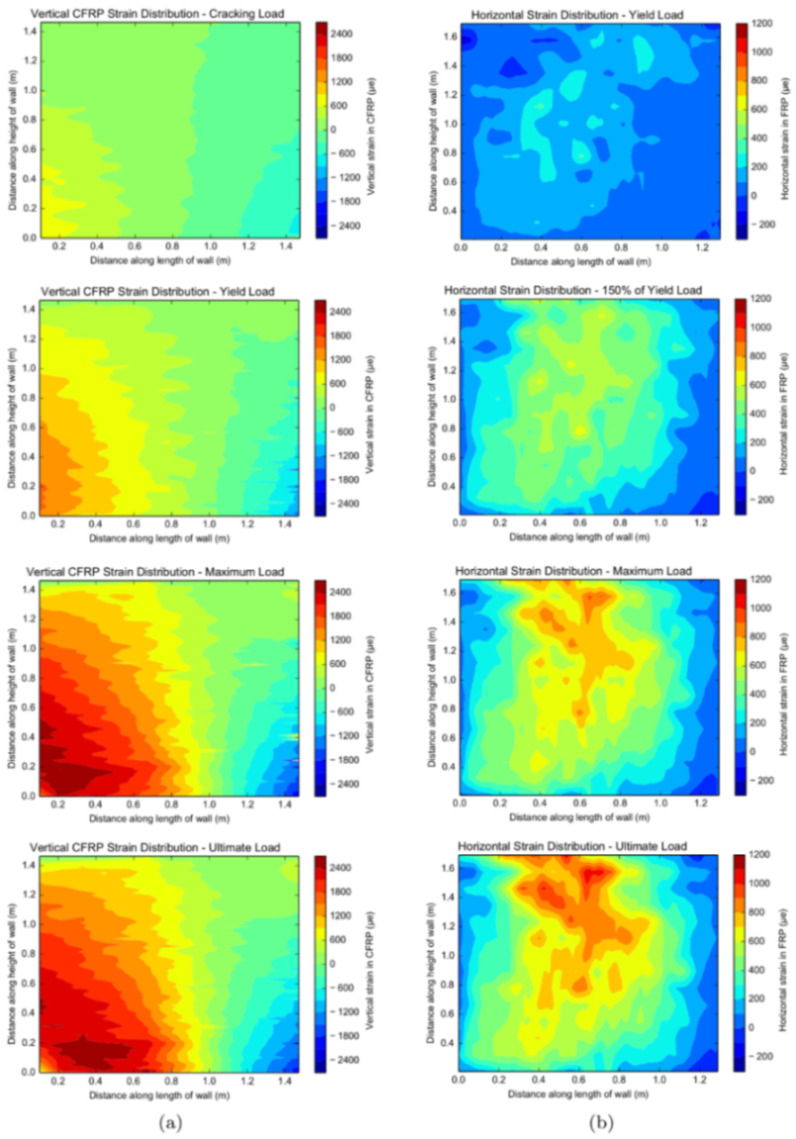
2D spatial strain distribution in the: (**a**) vertical and (**b**) horizontal directions [101].

The fibers were bonded horizontally (see Figure 35) to the surface of the outer-most CFRP layer (using an epoxy resin) and vertically on the back of the wall. This setup permitted an interesting and well-illustrated two-dimensional spatial strain measurement analysis over the entire area of the shear wall (see Figure 36), the study of the FRP contribution to the resistance of the shear wall and the capturing of the failure mechanisms. The authors concluded by stating that DOFS was durable, reliable and consistently providing strain measurements under repeated large strain reversals.

### 4.6. Sub-Section Conclusions

The present section was focused on the DOFS instrumentation of various structural elements subjected to laboratory experimentation. The reported efforts were categorized as RC beams, RC ties, RC slabs, corrosion testing and miscellaneous applications. Many of these, integrated DOFS monitoring with a novel technology named Digital Image Correlation (DIC) for the observation of superficial strains and for the cracking patterns of RC structures.

In the former category, the main observations were:

- The authors of most articles were successful in the identification of the flexural strains inside RC beams and a Cemented Pavement Material (CPM) beam, in the detection of the cracking patterns and in the monitoring of both the widening and the propagation of cracks;
- Both thin coating-less DOFS and high-resistance coated DOFS cables were successfully used to achieve the previous point;
- Polyimide-cladded fibers allowed for the early and accurate detection and localization of micro-cracks;
- For the bonding of thin coating-less DOFS on the surface of steel bars, the fibers were commonly positioned at the concavity created by the longitudinal ridge (after removing the mill-scale and degreasing the area with acetone) before gluing them with cyanoacrylate adhesive with the addition of a protective layer of a one-component water-proof oxygen-free silicone rubber;
- The strain of steel rebars embedded inside RC beams measured by means of DOFS and SG were found to be in good agreement both at crack locations and between the latter;
- In Berrocal et al. [59], the DOFS extracted pattern was compared against the one externally sampled by DIC with an agreement of ±3 cm between the two; a range which is understandable considering the different depth at which the transversal cracks were measured and their probable lack of perpendicularity compared to the beams’ axis. A good agreement (±20 μm) was generally observed between DOFS and DIC crack width;
- For the monitoring of shear failure in RC beams different but wholly valid solutions were proposed. The first saw the bonding of DOFS not only on the longitudinal rebars but also on 16 stirrups. The second saw the external deployment of an OFDR DOFS in a 2D grid on the shear span of a beam in order to obtain its shear cracking pattern. The third, deployed multiple fibers both internally and externally to the beam including an upward guided one running perpendicularly to the oblique shear cracks. All three deployment kinds successfully reported the steel strains, crack patterns and crack widths;
- OFDR DOFS bonded to longitudinal RC beams’ rebars were able to monitor their strains whilst withstanding cyclic loadings all the way to failure;
- OFDR DOFS were successfully employed for the study of the steel/concrete bond degradation due to reinforcement yielding inside RC beams subjected to lateral loading, thus demonstrating their monitoring potential even for high magnitude strains.

The DOFS monitoring of RC ties was mainly used to illustrate the cracking, deformation and bond behavior of RC structures thanks to the latter simplicity and reasonably good representation of the distribution of internal forces and strains in the tensile zone of RC structures. Indeed, most authors reported being able to extract plausible concrete–steel bond-stress values by means of RC ties double pull-out testing. Some key observations follow here:

- Zhang et al. [79] reported that the DOFS measurement sensitivity parameter decreased with an increase in the cable coating thickness due to both elastic and inelastic coating deformations. The authors further reported a certain influence on the DOFS-concrete bond exercised by the coating stiffness and the cables’ surface properties;
- Sienko et al. [80] raised the issue of the possible existence of an additional source of inaccuracies in thick-coated fibers. Here, a lower crack-width assessment accuracy was attributed to the slip between the DOFS core and its external jacket;

DOFS integration to RC and Timber–Concrete Composite (TCC) slabs was commended by several authors who successfully detected the internal strains up until the onset of reinforcement yielding in addition to obtaining a comprehensive picture of longitudinal temperature distributions, the heat flux transfer process and the consequent strain distributions.

On DOFS-integrated corrosion testing:

- DOFS has the potential of becoming a crucial SHM tool thanks to their immunity to corrosion;
- DOFS monitoring was reportedly able to detect the strain distribution changes along the longitudinal reinforcement due to the corrosion-induced loss of bond between the concrete and reinforcement;
- Lv et al. [92] introduced the idea of a so-called “corrosion sensor”. It consisted first of all, of a 5 mm thick mortar layer poured directly on the rebar around which a DOFS is tightly winded for several turns with a final mortar layer cast on top;
- The influence of a winded DOFS on the steel–concrete bond strength was studied by means of several pull-out tests. It was noticed that whilst no bond alterations were observed between a fiber-less reference specimen and longitudinally bonded fiber specimen, the spirally bonded one led to a certain bond reduction;
- The spiraling DOFS around a steel rebar deployment technique was used for the assessment of the influence of various parameters (concrete cover, amount of embedded reinforcement fibers) on the amount of corrosion suffered by the rebar.

Finally, particularly noteworthy miscellaneous experimental DOFS applications included steel and RC structures fire testing, the testing of Textile Reinforced Cementitious Matrix Composites and the shear testing of a Reinforced Polymer (FRP) RC wall.

## 5. Monitoring of the Built Environment: Buildings, Bridges and Roads

The present section focuses on the built environment branch of Civil Engineering of which its most conspicuous examples are buildings, bridges and roads but which also includes power plants, dams, railroads and airports amongst others. These structures cover key roles in the proper functioning, security and comfort of any society, thus their time-induced deterioration and serviceability-jeopardizing issues occurring throughout their service lifetime (e.g., corrosion, fatigue, creep and shrinkage-induced shortening and cracking) should be treated with the equivalent criticality. Revealing data, averagely representative of the situation of most countries worldwide, is provided by the American Society of Civil Engineers (ASCE)’s 2017 Infrastructure Report Card [102]. According to the latter, the U.S. has 614,387 bridges, four in 10 of which are 50 years old or more. Of the nation’s bridges, 9.1% were structurally deficient in 2016 and, on average, there were 188 million trips across a structurally deficient bridge each day. Furthermore, one out of every five miles of highway pavement is in poor condition. The most recent estimate puts the nation’s backlog of bridge rehabilitation needs at USD 123 billion and of highways rehabilitation needs at USD 420 billion. As observed by Regier and Hoult [103], it is not feasible to replace all the structures that have surpassed their intended service lives because of the budget, logistical and environmental concerns that such widespread demolition and reconstruction process would bring along. The only other possible approach consists in keeping the assets that are still fit for purpose in service. Hence the importance of determining their deterioration level, serviceability performance and public safety. Though visual inspection is the most common assessing methodology [104], it inherently includes a certain degree of subjectivity together with evident limitations when it comes to damages occurring on the inside of a structure or located in unreachable locations. As such, the SHM of Civil Engineering infrastructure proposes the definition of damage identification strategies performed through accurate quantitative data sampling tools and followed up by their continuous and real-time monitoring.

The applicability and performance of DOFS to the built environment SHM has been tested and proved possible in pioneering publications such as Regier and Hoult [103] Glišić et al. [105], Matta et al. [106] and more. Interestingly, Glišić et al. [105], reported the Brillouin scattering-based DOFS monitoring of a 1000 m long bridge (composed by a concrete slab poured on nine steel girders that are then supported by more than 50 columns) which is still currently ongoing, effectively making it, to the authors’ knowledge, the longest DOFS monitoring implementation on a real structure.

**Figure 37:**
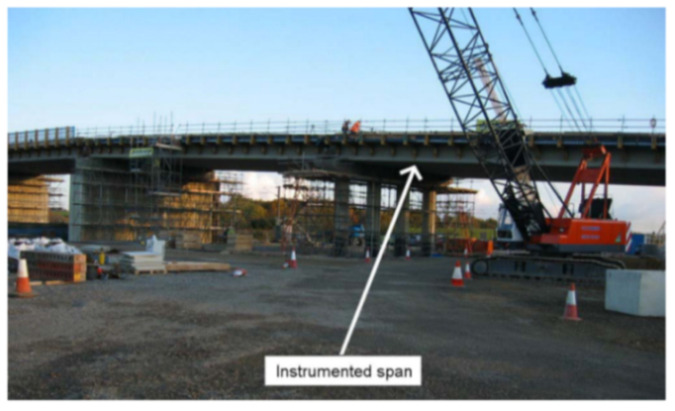
Nine Wells Bridge [107].

Webb et al. [107] picked up on the previous concepts to perform a BOTDR DOFS monitoring of the Nine Wells Bridge in Cambridge (UK) carrying a new road over the main rail line connecting Cambridge to London (represented in Figure 37).

**Figure 38:**
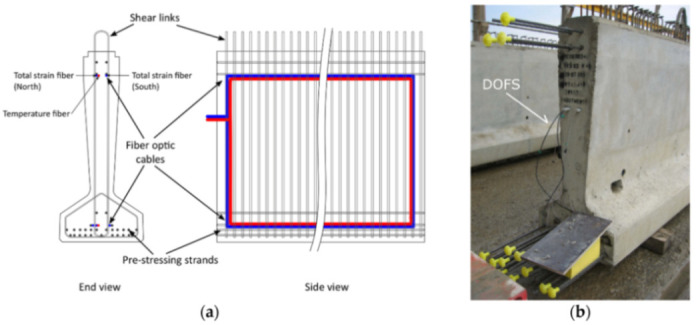
Beam cross section showing the fiber-optic cable locations: (**a**) illustration and (**b**) photograph (adapted from [107]).

The bridge, a three-span, pre-tensioned, prestressed concrete beam-and-slab bridge was monitored with the goal of observing the long-term behavior of the structure for damage detection purposes and in order to compare the in-situ measurements to the various empirical creep and shrinkage models used in the design phase. Six beams were instrumented with fiber-optic cables (as in Figure 38) after the prestressing strands had been pre-tensioned but before that steel reinforcing stirrups had been tied in place in the mold.

In particular, two types of fiber-optic cables were installed in the six beams; one for the sensing of mechanical strains (defined as total strain fibers) and one for the temperature strains (defined as temperature fibers). Interestingly, the latter cable was filled with the gel inside which the sensing fibers were suspended (this way, the shear transfer between the concrete and the sensing core was prevented). With such dual fiber deployment, the temperature effects on the Brillouin frequency shift could be removed, such that the true strain in the fiber (due to applied loading as well as time-dependent effects and the thermal response of the structure) could be calculated. The authors concluded by remarking that the measured creep and shrinkage-induced strains were found to be in reasonably good agreement with both Eurocode 2 [108] and Collins and Mitchell [109]’s models.

**Figure 39:**
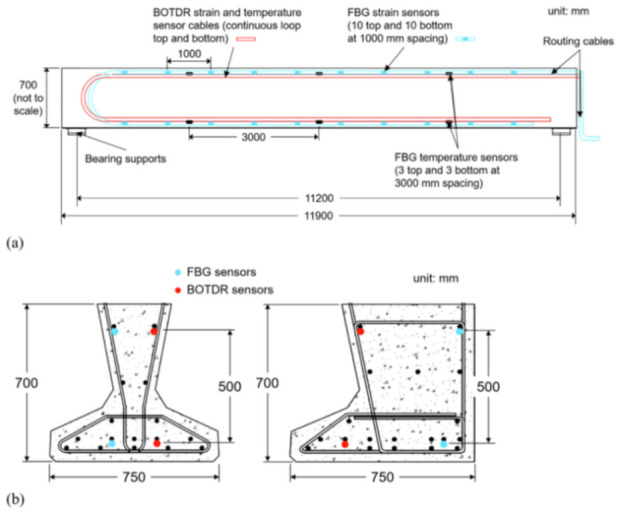
(**a**) DOFS deployment layout and (**b**) prestressed concrete beam cross-sections—edge TYE7 beams (left) and internal TY7 beams (right) [110].

Similar investigation procedures were undertaken by Cong et al. [110] whilst attempting to evaluate the prestress loss of four 11.9 m prestressed concrete beams of a newly-constructed railway bridge in Staffordshire (UK). Both immediate prestress losses (caused by the elastic shortening of concrete) and the time-dependent prestress losses (due to steel relaxation, concrete shrinkage and creep) were captured by means of distributed (BOTDR DOFS) and discrete (FBG) fiber optic sensors installed during the manufacturing process (as visible in Figure 39). The latter sensors measured both strains and temperature (for compensation purposes).

The measured prestress losses were compared with the predicted ones calculated according to both the European and American standards. The outcome was an apparent underestimation from the code’s part (possibly due to the inaccurate estimations of various input parameters) which was more pronounced in the early-age versus compared to the two to three years’ mark.

**Figure 40:**
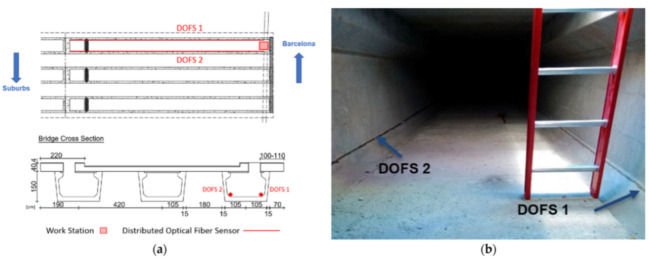
(**a**) General scheme of DOFS monitoring and (**b**) its photograph inside the box girder (adapted from [111]).

An additional bridge OFDR DOFS monitoring is reported in Barrias et al. [111]. Here, Sarajevo Bridge (Barcelona, Spain) was monitored during some deck-enlarging construction works in order to perform a follow-up of the stresses induced in its three prestressed concrete box girder beams. Thus, the shorter of the two bridge spans saw the longitudinal gluing of a single 50 m DOFS inside one of its beams (as visible in Figure 40).

The authors reported the strain evolution during the construction phase and calculated that, despite the strain variation (biggest one being −304 με), excessive stresses were not transmitted to the concrete during the construction works (maximum 11.42 MPa).

A critical scenario during Civil Engineering works might ensue after the pouring of mass concrete structures (such as dams, nuclear plants, massive foundations, bridge piers, thick slabs and skyscraper columns). Indeed, after the concrete is poured, the cement hydration reaction produces a large internal temperature upsurge. Due to the poor thermal conductivity of concrete, a huge gradient forms soon after between the extremely hot core of the pouring and its quickly cooling outer surface. Considering the poor tensile strength of early age concrete, the rising thermal tensile stresses at the surface might cause thermal cracking. This could potentially lead to a structure prematurely failing to reach its serviceability requirements or even suffering a structural failure. Some solutions for the prevention of this phenomenon are aggregates precooling, pipe cooling, superficial thermal insulation and alternative bay construction [112]. Nevertheless, the importance of monitoring the concrete’s temperature during the construction period (pertaining to the SHM field) cannot be understated.

**Figure 41:**
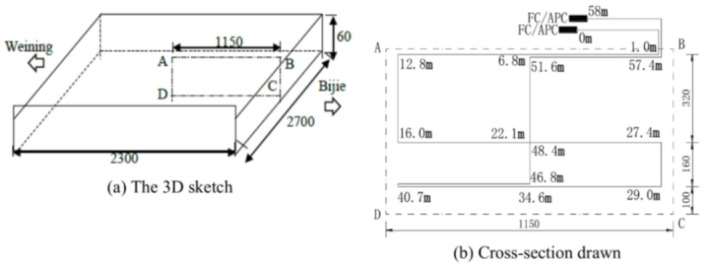
(**a**) A 3D sketch and (**b**) a cross-section of the DOFS deployment layout for temperature measurements in the study case pile cap [20].

**Figure 42:**
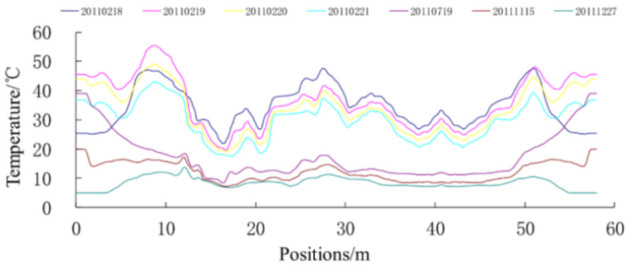
Temperature variation as a function of the DOFS coordinates specified in Figure 41 [20].

On such a topic, Li et al. [20] reported a BOTDA DOFS temperature monitoring of a bearing platform of a main pier pile cap pertaining to the 1073 m long Hezhang super-long-span bridge. The 58 m long optical fiber cable was deployed in a planar cross-section (as sketched in Figure 41) in areas resistant to damage and tied to the rebar using cable ties. The concrete temperature variations were measured for four consecutive days after pouring and three extra days in the long-term by means of both DOFS, point temperature sensors and precision thermometers for comparison purposes. The DOFS readings (characterized by accuracy of 0.3 °C) visible in Figure 42 were reported to be in satisfactory agreement with those extracted with conventional temperature sensors (the maximum difference being 0.23 °C).

Ouyang et al. [112] deployed a Raman-based DOFS to measure and monitor the concrete temperature during the sequential construction of an intake tower of the Qianping reservoir project, on the Huai River (China). In particular, the authors introduced a framework for the evaluation and control of the concrete cracking risk by integrating the DOFS measurements with an inverse analysis method based on temperature simulation and consequently thermal stress simulation. The DOFS was deployed on a horizontal double S layout (bonded on a steel frame) with the peculiarity that the single cable is passed through the designed path twice. As a result, the temperature distribution along the cable always ended up showing a mirrored profile which facilitated the mapping of the measuring points on the cable. As previously mentioned, in order to expand the significances of the local temperature measurements to the whole structure, numerical simulations of temperature and thermal stresses were conducted by FEM analysis. The effectiveness of the above methodology to the construction process, was verified when, upon the drilling out of several concrete cylinders (hydration around 90 days), their examination showed hardly any concrete cracking.

**Figure 43:**
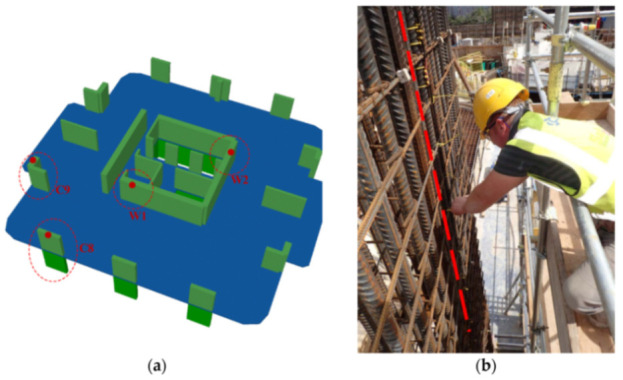
(**a**) Location of the instrumented columns and walls and (**b**) DOFS tied to the reinforcement prior to concreting [114].

Moving on from the bridge’s SHM, interesting results are reported by De Battista et al. [113,114] regarding the long-term BOTDA DOFS monitoring of two columns and two core walls (as in Figure 43a) of the 163 m, 50 storeys tall Principal Tower (London, UK).

The goal, assessing the combined structural shortening caused by the superimposed load, creep and shrinkage during the construction phase. 

**Figure 44:**
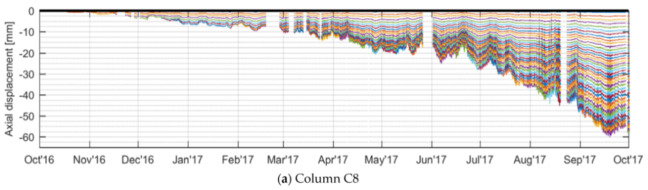

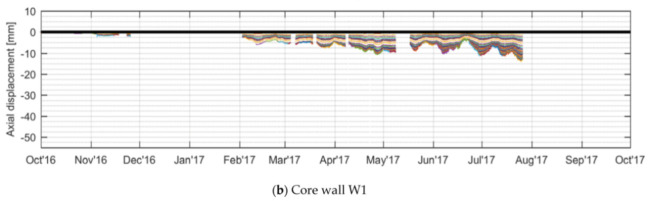
The total cumulative axial displacement (negative = shortening) of the instrumented columns C8 and wall W1 measured at the mid-height of every level during the first 12 months of construction (adapted from [114]).

Indeed, the authors highlighted the importance of establishing the floor-to-floor axial shortening of vertical load-bearing elements and their differential shortening (whenever they are characterized by different stiffness) for the installation of finishes and partitions on the lower floors. Thus the importance of integrating the designers’ empirical shortening calculations (adjusting them if necessary) with in-situ continuous distributed measurements. DOFS was then the ideal candidate for such goal, allowing for the calculation of the axial deformation at any time during the construction and along the whole height of the completed elements. Consequently, each monitored element was instrumented with two DOFS (measuring strains and temperature) fixed to the reinforcement (as in Figure 43b) before embedding them inside concrete. The DOFS monitoring started on 3rd September 2016 and since then measurements (with a spatial resolution of 1 m) were performed at least twice every hour. Expectedly, the authors detected that the columns shortened significantly more than the walls throughout the construction causing a non-negligible differential shortening between the two. For example, as visible in Figure 44 whilst column C8 suffered a shortening of 60.3 mm at level 38, simultaneously wall W2 shortened by only 10.2 mm.

This also occurred, with a smaller magnitude, between two columns with different cross-sections for example at the end of July 2017, the maximum shortening recorded at level 31 on column C8 was 36.6 mm whilst that on column C9 was 32.6 mm, approximately 11% less.

Qi et al. [115] also instrumented in a similar manner a structural wall pertaining to a deep foundation pit supporting system for the excavation of a subway station in Suzhou (China). Here, a PPP-BOTDA DOFS integrated more conventional monitoring methods, namely steel-bar meters, inclinometer, and water-level observation hole. According to the authors, at each excavation stage, the strains induced on the wall by the active and passive earth pressure were easily obtained by means of DOFS, thus leading to the assertion that the fiber could easily substitute the other classic tools.

Broth and Hoult [116] assessed the dynamic sensing capabilities of an OFDR DOFS system by performing in-situ monitoring of a 9.24 m cast-in-place RC T-beam located below a multi-purpose room located in the Queen’s University campus in Kingston, Ontario, Canada. The application of the dynamic loads was achieved by having 38 students align themselves across the full span of the classroom (distributed test) and tightly gather over the midspan of the beam (concentrated test) before repeatedly jumping in unison over the instrumented specimen. The DOFS output led the authors to confirm that, through the use of the OFDR DOFS system, both static and dynamic concrete strains could be successfully measured. Furthermore, the observed trends in the data from both the linear potentiometers and distributed sensors were in good agreement, suggesting the latter’s ability to accurately capture a beam’s true dynamic behavior. Finally, DOFS was reported to successfully provide the dynamic response factor of the instrumented structure, the distributed crack widths and the deflected shapes.

Brault and Hoult [117] investigated the use of OFDR DOFS to monitor three beams of a newly constructed RC building subjected to load testing. The investigated structures were three cast-in-place RC elements, namely a beam, a drop panel and a larger beam. The elements were first sanded along the fiber pathway, the latter was then cleaned with water and 99% isopropyl alcohol and, on it, the fiber was bonded with a two-part adhesive. The load test was performed as follows. For the beam and the drop, the measurements were performed prior to loading, during an initial application of the load (using two scissor lifts), during the positioning of six scissor lifts at once and finally once all scissor lifts had been removed. According to the authors, DOFS efficaciously captured inflection points, moment transfer at the supports, crack locations and crack openings. Finally, the authors reported the average crack spacings and the maximum measured crack openings.

Proceeding to the third and last topic of the present section, Wang et al. [118] used five BOTDA DOFS for the detection of transversal and longitudinal strains inside a multilayered asphalt pavement under heavy traffic and temperature loads. They pointed out that the deflection-induced tensile strains in the bottom pavement layers could lead to potentially permanent deformations under heavy traffic flow and at high temperatures. The DOFS extracted results revealed that the paving of the second asphalt concrete course could bring about large transversal and longitudinal strains on the previously built bottom layer due to the compressive rolling action. Later, when the road became operational, different tensile strain evolutions were observed in the transversal and longitudinal sections. For example, the asphalt pavement adjacent to the intersection exhibited high strain levels induced by vehicle turning and braking effects. It was therefore deemed prone to tensile strain-induced cracking. Large tensile strains were also detected along the vehicle-track direction, even though at a much lesser degree than the former. The daily temperature variations altered both transversal and longitudinal strains increasing (or decreasing) the measured strains of around 100 με.

**Figure 45:**
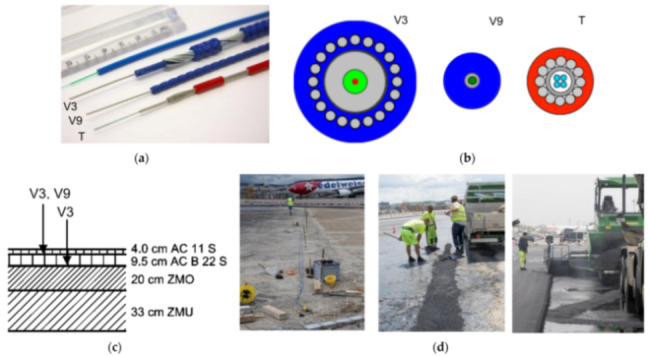
(**a**) Photograph of the DOFS cables, (**b**) an illustration of their cross-sections and (**c**) of their designed installation position and finally (**d**) photography of the deployment process (adapted from [119]).

Rabaiotti et al. [119] acknowledged the presence of similar risks for airfield pavements, especially when taking into consideration their considerably larger loading. The authors further elucidated that, for such a particular application, the main challenge lied in avoiding damage to DOFS during the asphalt compaction process which usually involves high temperatures (exceeding 140 °C). To tackle this issue, ad hoc strain (V3 and V9) and temperature (T) sensing cables were developed (illustrated in Figure 45a,b) using armored DOFS cables, metal tubes and PA outer sheaths.

**Figure 46:**
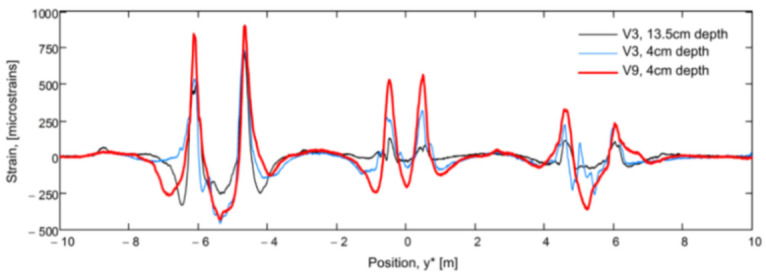
Strains measured along the sensors when the study case airplane rear landing gear was rolled on them [119].

In the present project, BOTDR and Rayleigh-based Swept Wavelength Interferometry DOFS were embedded in two pavement layers in 150 m long loops. The deployment steps were performed as follows. Initially, the fibers were laid out on the substrate layer, followed by their pre-straining and fixing in place (Figure 45c,d). After such, for better protection against the rolling compactor machine, the sensors were covered manually with finer asphalt, before the overlying layer was poured. The test started when a plane was pushed backward in a perpendicular direction to the fiber loop resulting in sampled strains illustrated in Figure 46.

The authors observed that the stiffer V3 cable seemed less capable of following the pavement’s high strain gradients versus its V9 counterpart. Additionally, the strains measured in the lowest layer (13.5 cm depth) were expectedly smaller. In conclusion, this DOFS-based testing methodology was commended for the detection of problematic zones and quality issues in the realized pavement in addition to validating the design assumptions.

**Figure 47:**
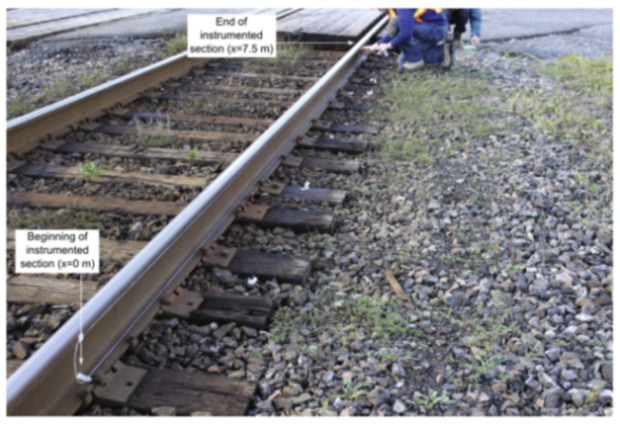
Installation of fiber on rail [120].

Finally, an additional transportation-focused SHM application is also reported in Wheeler et al. [120] where OFDR DOFS (OBR Luna ODiSI-B model) were employed for the assessment of distributed dynamic strains of a steel rail during the passage of rail traffic. A preliminary laboratory test was aimed at studying the difference in monitoring performance whenever the fiber was bonded to the rail with minimal and optimal (grinding and cleaning) surface preparation. After having observed that both yielded similar results, the authors commended the former as the best option for rapid field installations. Additionally, the authors defended the option of bonding the fibers to the rail foot and the railhead which, despite being prone to be affected by localized strains under heavy train wheels, allowed for more accurate rail curvature calculations thanks to the greater distance between the two DOFS. The following reported step was a field test performed near Kingston (Canada) where the DOFS was bonded according to the guidelines established earlier as visible in Figure 47.

**Figure 48:**
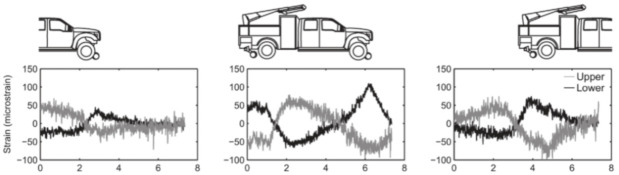
DOFS-sampled strains for three different hi-rail vehicle positions along the track (adapted from [120]).

Vehicular loading under low vibration conditions (hi-rail vehicle) and high vibration conditions (in-service passenger train) was applied on the rail. Figure 48 represents the strains measured by the upper and lower fibers for the hi-rail vehicle at three different positions along the instrumented section of the track.

The authors reported that it was feasible to study the variability in track support along a section of rail on the grounds of DOFS rail curvature measurements. Yet, they also acknowledged, that despite dynamic distributed rail strains producing low vibration could be measured, the same thing could not be said in the presence of high vibrations. Indeed, the quality factor of the strains resulting from the transit of the passenger train was below the DOFS manufacturer’s minimum recommended threshold.

Conclusively, the present sub-section started describing various DOFS applications for the SHM assessment of bridges. The following key observations were reported:

- DOFS can be embedded inside prestressed concrete beams, preferably combining a strain sensing fiber with a temperature sensing one in order to remove any temperature effect on the Brillouin frequency shift, such that the true strain in the DOFS can be calculated. Alternatively, for box girder beams, DOFS can be simply glued inside their hollow cross-section.
- A reported application described the temperature sensing cable to be filled with gel inside which the DOFS was suspended in order to prevent the shear transfer between the concrete and the sensing core;
- DOFS was successfully implemented inside bridge RC beams for the purpose of monitoring damages and both immediate and time-dependent effects (prestress losses for instance).

Furthermore, the temperature monitoring during the pouring of mass concrete structures (dams, nuclear plants, massive foundations, etc.) was successfully performed by means of both BOTDA DOFS and Raman backscattering-based DOFS with an accuracy of 0.3 °C.

On the monitoring of buildings by means of DOFS:

- The shrinkage and creep-induced shortenings (and differential shortenings) of the columns and structural walls of high-rise buildings can be monitored with a single BOTDA DOFS stretching across all the building’s floors. The sampled differential shortenings in such kind of structures can be very relevant such as was the case in the reported assessment of the shortenings of the Principal Tower C8 column (60.3 mm) and W2 wall (10.2 mm);
- Following both static and dynamic testing of multiple buildings’ RC beams, the authors of the relative publications state that, through the use of the OFDR DOFS system, both static and dynamic concrete strains could be successfully measured;
- The above mentioned DOFS-powered tests further reported an effective capturing of structural inflection points, moment transfer at the supports, crack locations and crack openings;
- In all cases, DOFS were bonded to the surface of RC elements after sanding the latter along the fiber pathway, cleaning it with water and 99% isopropyl alcohol.

Finally, on the monitoring of roads by means of DOFS:

- The strains transferred to a multilayered asphalt pavement can be monitored by means of embedded DOFS. This fiber deployment’s main criticality is during the asphalt compaction process which further involves high temperatures. To counteract any such adversity specific high-resistance DOFS cables were employed in addition to manually covering the DOFS with finer asphalt, before pouring the overlying layer;
- OFDR DOFS (OBR Luna ODiSI-B model) was employed to successfully assess the distributed dynamic strains of a steel rail during the passage of low-speed and low-vibration rail traffic but were unsuccessful for high-speed high-vibration ones;
- OFDR DOFS reported very similar reading qualities between a DOFS cable bonded to a steel rail with minimal and optimal surface preparation (grinding and cleaning);
- A suggested DOFS deployment methodology was bonding DOFS to the rail foot and to the railhead which, despite being prone to be affected by localized strains under heavy train wheels, allows for more accurate rail curvature calculations thanks to the greater distance between the two DOFS.

For the readers interested in further reading on DOFS-based SHM of rails, the authors refer to two recent comprehensive literature review articles, namely Du et al. [32] and Sasi et al. [121]. Instead, for further reading on DOFS for Civil and Infrastructure Engineering sensing the authors recommend Soga and Luo [15].

## 6. Geotechnical Applications

Geotechnical Engineering covers a wide range of applications amongst which pile foundations, soil movements (landslides and soil subsidence), stabilization anchors, mining and more. Each of these has the potential for a DOFS-powered SHM integration and their relative literature review will receive independent scrutiny in the present section.

### 6.1. Pile Foundations

**Figure 49:**
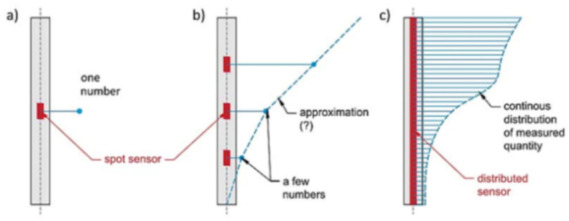
Measurement schemes for concrete piles: (**a**) spot, (**b**) quasi-continuous, (**c**) distributed [123].

Starting from pile foundations, these are commonly used whenever vertical loads are too high to be carried by shallow foundations (like in the case of high-rise buildings) and/or when the soil’s bearing capacity is not suitable to carry the designed loads. Now, considering the rapid urbanization process occurring nowadays all over the world, skyscrapers and mega infrastructure are becoming commonplace solutions to the novel requirements of our modern-day society. As such, pile foundations have seen increased use in the latest years, bringing along a stronger focus on the methodologies for testing and monitoring their performances. In particular, large research efforts have been dedicated to surpassing the limitations intrinsic to the traditional monitoring tools (such as SG) commonly employed for performing the required tests. Indeed, traditional sensors are easily destroyed in complex geotechnical environments and are prone to rust damage and electromagnetic interference [122]. Predictably though, their largest limitation is the punctual nature of their measurements. This is especially off-putting when compared against the much broader global structural behavior whose knowledge is required by an engineer to draw conclusions on the carrying capacity of the structure. Instead, as foreshadowed in Section 2, OFS are characterized by corrosion immunity, high durability, resistance to electromagnetic interference, small size, lightweight and, most crucially, they easily surpass the punctual measurement limitation. In more detail, as clearly illustrated in Sienko et al. [123] for the example of pile monitoring, traditional monitoring tools can carry out measurements only on a punctual scale (as visible in Figure 49a). Whilst the situation can be improved by installing several sensors along the monitored axis (quasi-distributed sensing illustrated in Figure 49b), the resulting profile still lacks sufficient accuracy to properly represent the actual pile strain profile. Distributed sensing, instead, provides complete access to this data (Figure 49c).

The two kinds of pile whose testing methodologies have received a significant performance boost from the integration of DOFS are the Continuous Flight Auger (CFA) or Auger Cast Piling and the Rotary Bored Piling. Research articles on their testing procedures are herein reported.

Starting from the CFA, when constructing piles under groundwater level or soft ground conditions, in order to prevent the collapse of the soil after drilling the borehole, continuous down–up concreting is performed as the auger drill is extracted (the concrete is pumped through a hollow shaft in the auger). The concrete hinders the penetration of groundwater and soil in the borehole. A reinforcement steel cage is then inserted in the filled borehole as soon as the concrete injection is terminated. Yet, as stated by Seo [7], the use of the CFA is not entirely failure-proof. Indeed, the force distribution along a column and its base’s load-bearing capacity might be affected by issues such as cross-sections reductions or bulge formations (soft soils), soil mining problems (loose gravel and sand can be mixed with concrete materials at the bottom of the pile), hole stability and auger rates control problems (ground with voids or water pockets) [123]. The assessment of the column force distribution and base load-bearing capacity is, therefore, a key aspect of SHM and was, therefore, the topic of numerous experimental campaigns.

**Figure 50:**
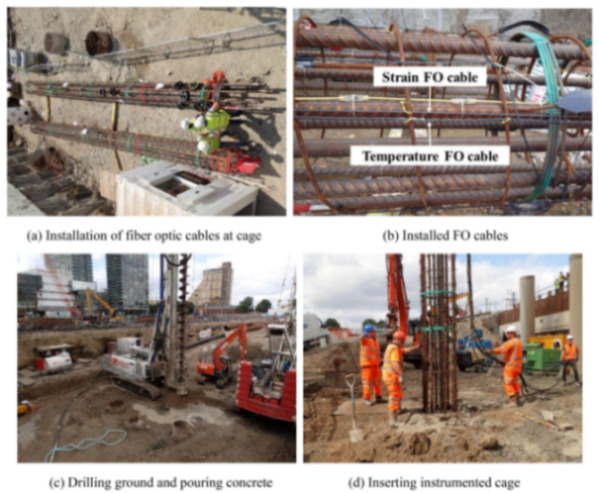
Installation of the DOFS and the pile [7].

Seo [7] instrumented the steel cages of a 25 m deep CFA pile with both strain and temperature BOTDR DOFS (as visible in Figure 50) in addition to standard SG and extensometers. Interestingly, a certain prestress was applied to the strain DOFS in order to facilitate the recognition of its frequency data location during the data analysis (its central frequency was, therefore, higher than the temperature’s cable). The compression load was initially increased to 8 MN, it was then unloaded and finally loaded again up to 20 MN. Seo assumed that if the soil had mixed with the concrete at the bottom of the CFA pile, large strains would be reported in that location. However, the maximum compressive strain was only 200 με, thus confirming the proper functioning of the pile. Finally, Seo studied the pile’s skin friction at its bottom reporting a significant increase with the applied load.

**Figure 51:**
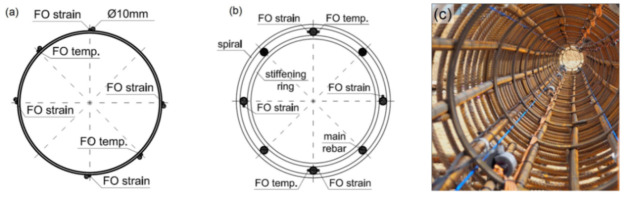
Typical cross-section layout of DOFS cables attached to (**a**) a steel pile and to (**b**) a steel cage; photography of the latter is represented in (**c**) (adapted from [124]).

A similar testing methodology was presented in Kania et al. [124] who monitored, by means of OFDR DOFS, the strains and temperature inside different kinds of piles subjected to static load testing. The authors provided a comprehensive look at the proper way to perform a DOFS structural instrumentation. Such work is commendable as it can provide an initial quality boost to similar future tests and, as such, some key aspects are reported in the following. First of all, if the pile may potentially be subjected to bending, Kania et al. suggested the mounting of a pair of strain sensors in opposite locations in order to measure both its compressive and tensile strains. Furthermore, they recommended to always install two fibers at each location for both redundancy purposes and, in case of damage or unexpected results, for having access to independent verification data. Then, similarly to what was done in [7], they introduced the idea of a pre-tension of the DOFS cables to an amount larger than the expected compressive strain in order to align its length scale. Finally, once the sensing cables are fixed to a structure, they proposed the marking of the points of interest (e.g., pile head, specific depths, and pile toe) by pressing or heating/cooling the cable in that specific point/s and recording the location of the signal. In [124], the piles in question were a steel pile, a CFA pile and a precast pile which were all instrumented with different fiber deployment methodologies. For the bonding to the steel pile, additional steel rebars were welded to its surface as a guide and protection for the DOFS (see Figure 51a). The fibers were bonded to the CFA pile’s reinforcement cage with epoxy glue (see Figure 51b,c) and, for the precast concrete pile, they were installed into previously incised grooves and then fixed with epoxy.

**Figure 52:**
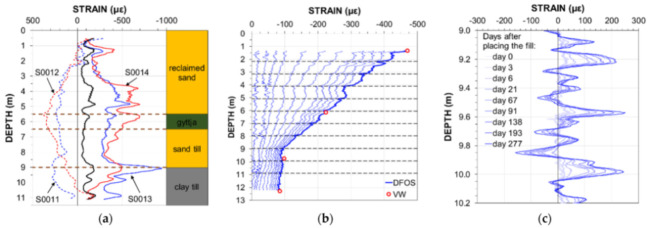
Distribution of strains along individual DOFS (**a**) after the driving of a steel pile (**b**) during a static load testing of the CFA pile and (**c**) the crack evolution inside a precast concrete pile (adapted from [124]).

The authors studied and reported the steel pile’s strains induced by its driving in the ground (Figure 52a), the CFA pile’s strain distribution during a test which included twelve incremental load steps (Figure 52b), the precast concrete piles’ operational strains (which were influenced by the evolution of several cracks as visible in Figure 52c), the short and long term influence of temperature on the sampled strains and finally the influence of lateral forces.

Sienko et al. [123] also performed a 12 m long strain and temperature OFDR DOFS monitoring on a CFA pile. The article reported the pile strains sampled during a multi-load steps compression test in addition to its accumulated shortening. The strain profiles clearly showed some local extremes which were attributed to a reduction of the column stiffness (reportedly due to the reduction of the pile’s cross-sectional area or the concrete’s modulus of elasticity).

Moving on to Rotary Bored Piling, differently than CFA, after drilling these require additional ground support such as casing or drilling fluids but can reach much deeper depths ~60 m (versus its CFA counterpart ~25 m) thus being able to accommodate higher loads, bypass underground impediments and infiltrate the ground which could otherwise be too hard for CFA drilling.

**Figure 53:**
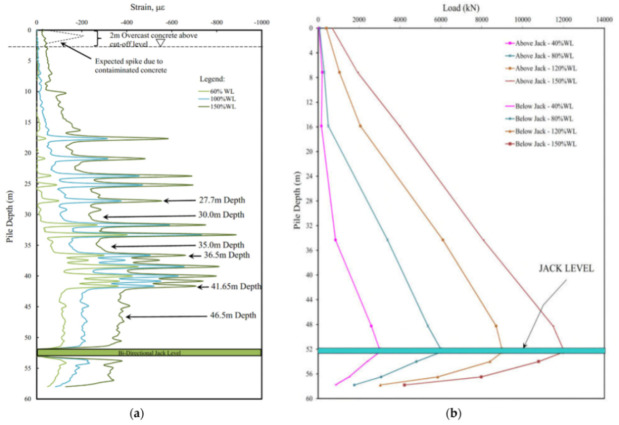
(**a**) Changes in strains along the pile during Bi-Directional Static Load Test (BDSLT) and (**b**) the calculated load distribution along it (adapted from [125]).

Cheng et al. [125] reported the outcome of a Bi-Directional Static Load Test (BDSLT) on a BOTDA DOFS instrumented bored pile with the aim of verifying the geotechnical design parameters for the highly fractured limestone formation on which the pile’s residential building project was located. BDSLT represents a deviation from the classic but reportedly inferior (time, cost and risk wise) Maintained Load pile Tests (MLT) as it avoids the latter’s massive reaction systems (i.e., dummy piles, platforms, reaction beams or concrete blocks) in favor of jacks incorporated inside the pile shaft itself. Whilst the conventional MLT systems apply the testing load from the top, the BDSLT’s bidirectional jacks expand separating the pile’s body into the upper pile section and lower pile section (shafts) pushing the former upwards (mobilizing the shaft friction) and the latter downwards (mobilizing the shaft resistance and end bearing). The study case pile was 57.3 m long with a 1.35 m diameter and the bidirectional jack embedded at 5.8 m from the pile toe. Its axial strains were sampled with four single-core DOFS, reinforced by several strands of steel wires and a polyethylene jacket, bonded to four sides of the reinforcement cage at 90-degrees from each other. Figure 53a displays the continuous axial strain profile at several loading stages of the working load (WL).

The authors reported witnessing a consistent DOFS strain increment as the load increased along with unexpected spikes (between 16 m to 43 m depth) on all four independent fibers likely due to the pile’s stiffness variation (similarly to [123]). Despite acknowledging the strong possibility of the bored pile’s shape non-uniformity and concrete stiffness inconsistency, due to the inaccessibility of the stiffness data, the authors assumed it constant for the calculation of its axial forces (Figure 53b). The sampled DOFS strains successfully diagnosed an unexpected and potentially disruptive low shaft friction, hypothized to be caused by unaccounted features of the karstic limestone and of the inclined bedrock.

**Figure 54:**
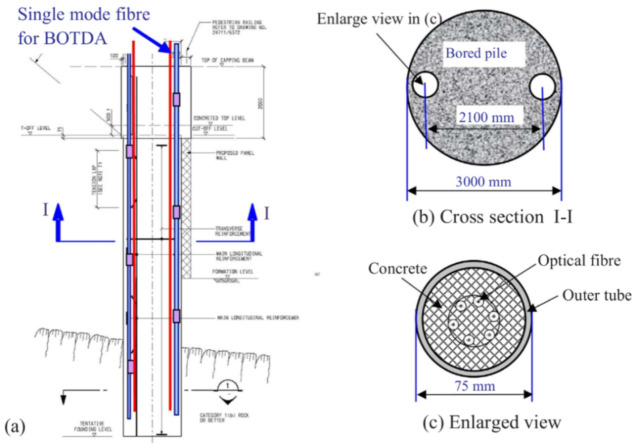
Fiber optic deployment layout in the study case bored pile [126]. (**a**) Total view;(**b**) Cross section I-I; (**c**) Enlarged view.

Xu et al. [126] and Pei et al. [21] presented a new bored piles BOTDA DOFS installation methodology and reported the outcome of its application to three bored piles (20.9 m embedded depth and 3.0 m diameter) pertaining to a retaining wall reinforcing a soil slope subjected to multi-stage excavations. Similar to [124], in order to obtain both the axial and bending strains along an instrumented pile, two sets of DOFS sensors were installed symmetrically with respect to their axis (as in Figure 54).

The maximum lateral wall deflection was assessed to be 0.05–0.1% and the measurements obtained from the BOTDA DOFS were found to be in good agreement with the one coming from parallelly installed inclinometers.

Guo and Zhao [127] performed a BOFDA DOFS monitoring of two 39 m long and 1.5 m diameter bored piles buried in a sloping terrain thus also subjected to both vertical and horizontal loading. The DOFS was bonded to the pile’s steel cage to form a U-loop monitoring its two sides and its bottom. Once again, strain profile peaks were present and were attributed to inhomogeneities of the pile’s material. The pile’s axial force calculated per DOFS was compared with the ones extracted by an embedded steel stress meter with good agreement between them.

Finally, Gao et al. [18] proceeded in a very similar way instrumenting a bored pile with U-looping single-mode GFRP-covered BOTDR DOFS fixed to its steel cage. Additionally, the results were filtered using three different noise removal methods.

### 6.2. Soil and Rock Deformations

Ground deformations are key study points for Geotechnical Engineering (land subsidence, ground fissure and surface collapse amongst others) as serious geological disasters may occur on their back. Their monitoring and the development of related early warning systems are crucial for the prevention and control of such disasters. Conventional technologies such as tiltmeters, inclinometers and multipoint extensometers have been adopted for this purpose for decades but the high costs and logistical issues associated with the creation of automated systems make them impractical for long term and massive deployments [128]. In response, the industry is slowly shifting to alternative and more performant ground deformation monitoring technologies such as DOFS.

The first scrutinized geotechnical issue will be land subsidence, explicitly the gentle down warping and/or sudden sinking of discrete segments of the ground surface [129]. One of its main causes is the intense extraction and exploitation of groundwater (but also of crude oil and natural gas) which leads to the compaction and collapse of underground aquifer systems. Soil subsidence has been the topic of an intense DOFS-powered investigation effort spanning multiple articles concisely presented in the following paragraphs.

**Figure 55:**
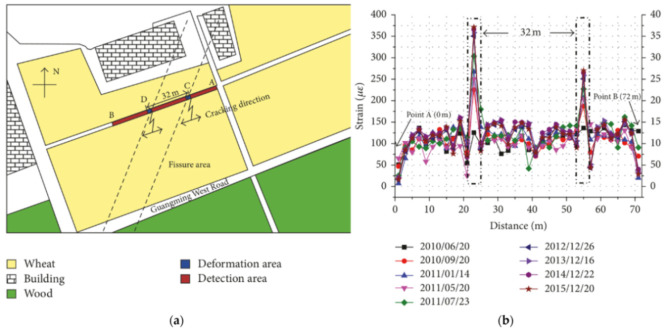
(**a**) Monitoring area plan and (**b**) resulting strains (adapted from [128]).

Liu et al. [128] performed a long-term BOTDA DOFS monitoring of subsidence-induced ground fissure deformations of a field in Wuxi City, China. A special high-strength optical fiber was used with a measurable strain limit of 15,000 με. The DOFS was placed in a 32 m long, 75 cm deep and 45 cm wide trench excavated at a 45 degree angle compared to the fissure direction. In order to compensate for the effect of seasonal temperature variation, a temperature-sensing optical fiber was also inserted in the DOFS-containing tube (placed for protection purposes). In the span of almost 5 years, nine measurements were carried out indicating a ground subsidence-induced total cable stretch of 11.3 mm. The latter was mainly concentrated in two areas (Figure 55a,b) where the largest strain evolutions were reported.

Wu et al. [130] applied DOFS technology to a laboratory experimental study on land subsidence caused by seasonal water level fluctuations and dewatering. In particular, the specimen was a small-scale sand–clay interbedded model box built for the purpose of carrying out consolidation and rebound tests during two drainage–recharge cycles. In order to analyze the deformative response of each of the specimen’s layers to the water level variations, the authors placed inside it two different sensing cables (a special tight-buffered PPP-BOTDA single-mode DOFS and a carbon fiber tube with FBGs) with small Plexiglas disks fixed perpendicularly to the cables in order to increase the soil/fiber bond. The PPP-BOTDA DOFS-extracted results indicated that the layers were compressed during drainage and rebounded during recharge, with larger deformations in the clay layer than in the sand. Furthermore, the deformation of the sand layer was synchronous with the water content variations, whilst the clay’s lagged behind due to its lower permeability coefficient.

**Figure 56:**
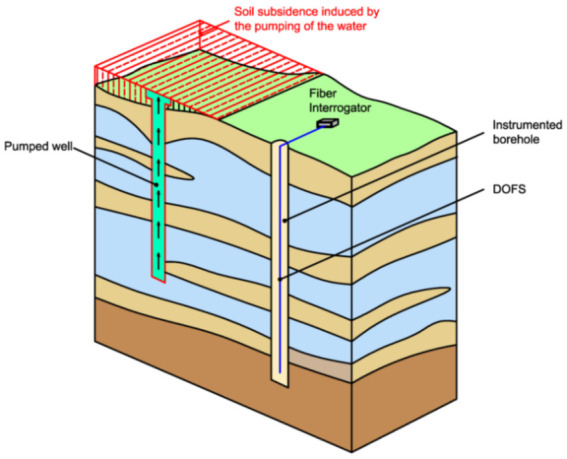
Illustration of soil subsidence and DOFS vertical deployment in a borehole as performed in [131].

The same authors published in Zhang et al. [131] the results of an in-situ experimental campaign (Suzhou, China) aimed at acquiring data on the subsidence and strata subsurface vertical deformations by means of a BOTDR DOFS embedded in a borehole (as in Figure 56).

After the 200 m deep borehole was excavated, the DOFS cable was attached to a cone guide and lowered into the former. The hole was then backfilled with a mixture of sand, clay and gravel. Then, by means of direct integration, the authors converted the DOFS strains sampled between December 2012 and November 2014 into a numerical estimation of the compaction or rebound that occurred in that time. The results highlighted the rise of positive strains between 60.3 m and 67.9 m depth indicative of an overall rebound of the pumped aquifer.

Liu et al. [22] performed a very similar monitoring campaign in a 130 m deep borehole in the Yangtze River Delta region (China) using BOFDA DOFS technology. The authors specify having backfilled the drilled borehole in accordance with the surrounding stratum type for consistency purposes. The monitoring, which lasted 2 years, successfully detected the occurrence of compression-rebound cycles simultaneous to the draining–recharging one. Additionally, being the soil rebound smaller than that of compression it was assessed that irreversible deformations had occurred. Similar to all the previous articles, the authors concluded suggesting that DOFS provides clear advantages for the analysis and early warning of land subsidence mechanisms.

According to Cheng et al. [17], an additional purpose that DOFS could cover is the monitoring of drying-induced shrinkage and desiccation cracking in clayey soils. In order to investigate such possibility, the authors parallelly placed three differently coated optical fibers (thermoplastic polyester elastomer jacket, nylon jacket and acrylate coating) in a mold before pouring the soil slurry. The authors reported BOTDR DOFS-sampled strains in the order of several micro strains when the soil was over-saturated, their gradual increase with the drop of water content (and the increase in the soil/fiber interfacial shear stresses) and finally their drop after the development of desiccation cracks (reportedly due to fiber/soil decoupling). The observations on the performance of each fiber and their strong impact on strain measurement are herein reported. The optical fiber with acrylate coating was deemed not suitable for the test due to its fragility and the poor interfacial coupling with the soil. Oppositely, the optical fibers covered with TPEE jackets and nylon were deemed suitable with the former being more sensitive to water content variations.

Soil shear deformations are a supplementary geotechnical parameter to closely monitor for stability assessments and for early warnings of potential geo-hazards [132]. The following articles present DOFS-powered SHM campaigns developed in this framework.

**Figure 57:**
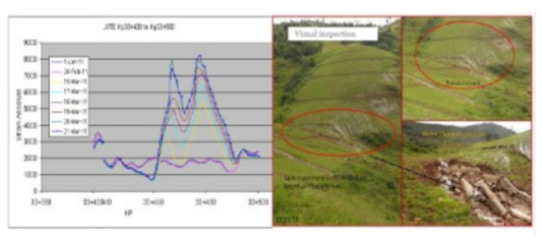
Monitoring chart and site map of pipeline trench collapse accident [133].

Wang et al. [133] reported the results of a BOTDA DOFS monitoring of the 408 km long Trans-Andean Pipeline (Peru). Given that soil shear movements could lead to movements, deformations and even fracture of the pipeline, an IEC 794-1 standard DOFS cable (capable of monitoring movements as small as 5 cm) was positioned in the former’s trench in order to detect the appearance of any such phenomena. Early warnings would trigger a patrol’s damage assessment and the prompt strengthening of the pipeline whether necessary. The system reportedly warned of the occurrence of rockfall and a water seepage-induced collapse of the pipeline trench (as visible in Figure 57).

Zheng et al. [134] attempted to determine the performance of a BOTDR DOFS monitoring system for the detection of deformations induced by shear sliding on rock slopes. An initial direct-shear laboratory experiment was performed in order to investigate DOFS’ monitoring effectiveness in this environment, finding a high correlation between predicted and tested results (average ratio between 0.974 to 1.081). Following such, the setup was applied in an in-situ shallow artificial slope monitoring test whose results were, once again, reportedly similar to the predicted ones. According to the authors, these results suggested OFSs applicability and accuracy for slope deformation.

**Figure 58:**
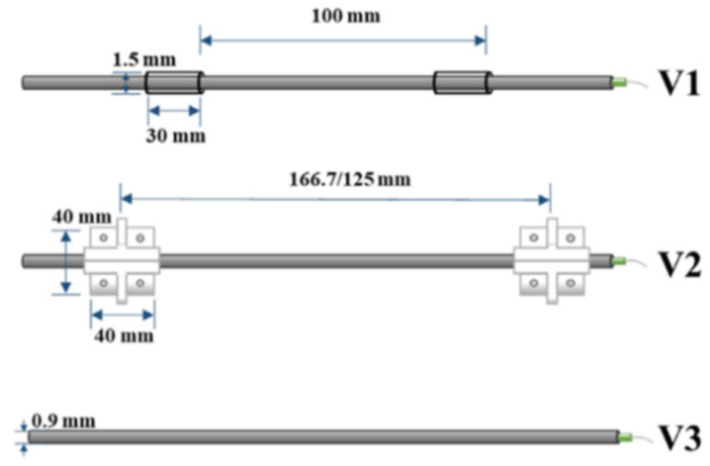
Different kinds of anchors used in the test from top to bottom: **V1** tube-anchored cable, **V2** aluminum block anchored cable, **V3** smooth cable [132].

On the same topic, Wu et al. [132] raised the question of whether the slip between OFDR DOFS cables and soil, that could potentially occur during soil shear deformation applications, would yield strain measurement errors. To answer the matter, the authors performed a laboratory direct shear test with different DOFS/soil anchorage methodologies. These included surface-mounted heat-shrinking tubes (V1 in Figure 58) whose post-shrinking local corrugations supposedly enhanced the cable-soil interfacial bonding, aluminum block anchors (V2) and no anchoring (V3) for reference purposes.

The test results showed that for small soil shear displacement (9–15 mm), good soil displacement estimations could be extracted even with no additional anchorage (V3). Beyond such displacements, though, the non-anchored cables slipped from the surrounding soil (leading to smaller detected strains) whilst the block and tube anchored ones still maintained a well-coupled state. The authors concluded by suggesting the use of block anchors for shallowly-embedded field applications and tube anchors for high overburden pressure scenarios.

Shear deformations were also studied in Schenato et al. [135] in the framework of a laboratory large-scale physical landslide model monitored with an OFDR DOFS deployed in the latter’s body. The shallow landslide was triggered by artificial rainfall which was sustained until complete slope failure. The authors’ most important takeaway was the possibility of observing through DOFS precursory failure signs well before the slope collapse thus being able to gain insights into its failure dynamic.

Zhang et al. [136], Chen et al. [137] and Xu et al. [138] remarked that adverse phenomena decreasing the carrying capacity of a railway subgrade soil (soil cave or karst sinkhole collapse) could severely impact the latter’s viability and train derailment risk. Thus, the importance of a train track subgrade SHM. The articles report a common BOTDR DOFS subgrade settlement monitoring test aimed at determining their viability for such kind of monitoring. In this test, a 4 m long DOFS was embedded into a soil subgrade crossing the roof of a 100 cm wide pre-made soil cave. The four stages collapse of the latter led to a 147 cm wide superficial collapse measured by means of both DOFS and micrometer. Their comparative maximum relative error was 5.28%. The common conclusion was that DOFS can effectively monitor a sinkhole collapse with relatively high precision.

### 6.3. Soil Stabilization Anchors

**Figure 59:**
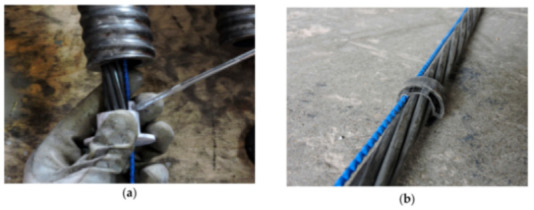
DOFS installation by means of plastic hooks for coupling the DOFS in an anchor’s (**a**) extremity bar and (**b**) internal bar [139].

After discussing soil deformations and landslide DOFS monitoring, the logical next step is the monitoring of stabilization anchors. Starting from Cola et al. [139], the authors set themselves to monitor the strains of an OFDR DOFS embedded with the tendons inside a composite anchor. These are special hollow steel bars coupled with tendons cemented in the inner hole to increase the global anchor tensile strength for the remediation of unstable slopes. The installation was reportedly performed in two steps. The first saw the installation of the composite anchors using a self-drilling technique whilst the second saw the insertion of one or more harmonic steel tendons instrumented with OFDR DOFS positioned in the bar’s inner cavity as in Figure 59.

Once the instrumented anchors were installed the system permitted a millimetric evaluation of the axial force distribution in the anchor, of the soil–anchor interface actions and of the stabilizing capability associated with the specific hydrogeological conditions of the site. According to the authors, the extracted strain profiles had similar profiles. All had their respective maximum strains sampled just behind the external plate and slowly decreasing strain values when moving inside the ground. The strains decreased to almost zero at about 16 to 17 m from the slope due to the activation of distributed shear stress along the soil–bar interface.

**Figure 60:**
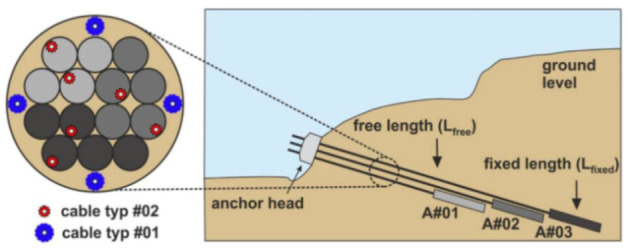
Schematic representation of single borehole with multiple anchors and with two DOFS deployed along the tendons of each individual anchor. Two fiber loops were also deployed in the grout material [140].

Lienhart et al. [140] proceeded in a similar way to monitor multiple Single Borehole Multiple strand Anchors instrumented with two OFDR DOFS and subjected to a pull-out test. The fibers were positioned along the tendons of each individual anchor along with two fiber loops in the grout material (see Figure 60).

**Figure 61:**
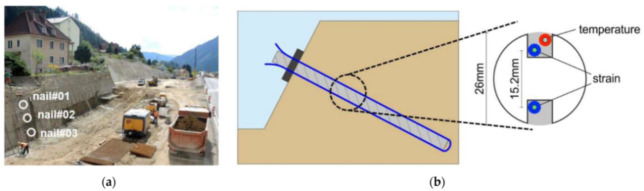
(**a**) Three fiber optic-instrumented soil nails at a construction site and (**b**) their cable layout [140].

The load tests included a stepwise load increase. Initially, the authors reported almost uniform strains along the free length of the anchor and decreasing strains in the tendons with increasing depth (due to the load transfer from the anchor to the soil). As the load increased, the load transfer area widened suggesting a progressive failure of the tendon/grout interface. The same article also reported a DOFS monitoring of the strain variation along the top and bottom of soil nails placed into a slope in a road construction site (as in Figure 61).

On the grounds of the distance between the top and bottom fibers, curvature changes were derived from their strain differences and a 7 mm vertical displacement at the front end of the nail was observed after roughly one month.

### 6.4. Mining

The last geotechnical sub-topic under scrutiny is DOFS monitoring for mining applications. In Zhang et al. [24] a similarity model was designed to simulate the rock overburden movement during the mining of a coal seam in Huainan, China. The original 800 m mining depth and its 2.5 m thick panel were simulated by means of a similarity model test (geometrically, kinematically and dynamically similar to the real-life case) carried out on a 4000 × 400 × 1607 mm plane stress model shelf instrumented with four DOFS cables connected to a PPP-BOTDA analyzer. DOFS successfully detected peak tensile strains in the soft strata and the interface of soft and hard strata.

A similar test setup was used in Chai et al. [25] in order to establish ground surface movement law for deep and extra-thick coal seam mining under the ultra-thick conglomerate. Similar to the previous article, the authors reported that the PPP-BOTDA DOFS technology could successfully and effectively capture the surface movements and deformations caused by mining.

Zhang and Sun [141] suggested that coal mining caused the movement of the overlying strata, thus leading to advance abutment pressure and consequently to the deformation and damage of the floor rock strata. As such, the authors developed a two-dimensional geological model of the advanced abutment pressure on the grounds of BOTDR DOFS strain readings performed in a borehole situated in Inner Mongolia. Such a model was then successfully validated by means of another borehole monitoring performed in the same mining area.

**Figure 62:**
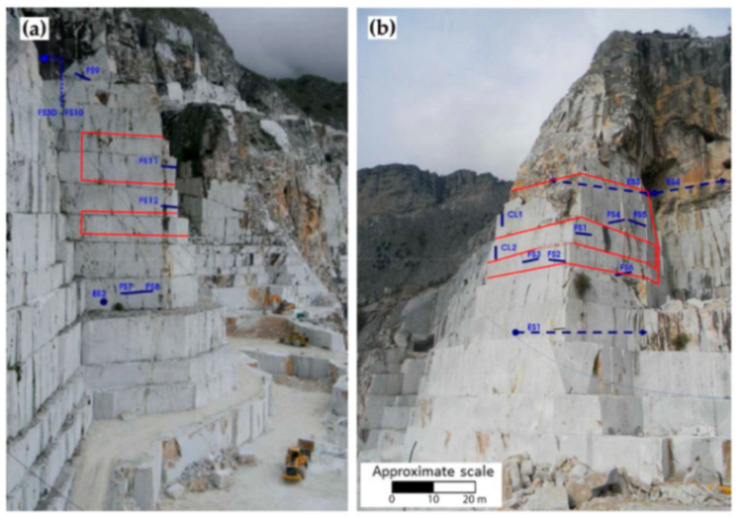
Geotechnical sensors (in blue) and fiber optics cable (in red) on the western (**a**) and southern-eastern (**b**) sides of the buttress [142].

Finally, Lanciano and Salvini [142] presented an innovative combination of DOFS (as visible in Figure 62), digital photogrammetry through Unmanned Aerial Vehicle, topographic and geotechnical monitoring systems for the monitoring of the rock mass stability conditions in marble quarries in order to eliminate the risks of rock falls. By means of a BOTDA DOFS monitoring system, some displacements were successfully detected despite their values fell within the tolerance threshold range. No other critical situations were detected.

### 6.5. Sub-Section Conclusions

The present sub-section presented various DOFS SHM applications for the assessment of the performance of foundation piles, for the monitoring of soil movements (i.e., subsidence, drying-induced shrinkage, soil shear deformation, landslides), for the monitoring of soil anchors and mining-induced deformations. On the assessment of the performance of foundation piles:

- The most commonly employed deployment technique was bonding DOFS to a pile’s steel cage before embedding the latter inside concrete. DOFS deployment techniques were also suggested for steel pipes (bonding the fiber on its surface with the addition of steel rebars welded to its surface as a guide and protection for the DOFS) and on pre-cast piles (DOFS were installed into previously incised grooves and then fixed with epoxy);
- Oppositely mounted strain sensors (or a single U-looping one) were used to measure both compressive and tensile strains in those situations where flexural strains are expected (piles pertaining to retaining walls for example);
- The installation of two fibers at each location was recommended for both redundancy purposes and, in case of damage or unexpected results, in order to have access to independent verification data;
- In more than one article, DOFS reportedly provided crucial data on the pile stiffness reductions possibly due to the decrease of its cross-sectional area and/or loose gravel and sand mixing with the concrete.

On the monitoring of soil movements:

- For the monitoring of soil subsidence, multiple publications positioned DOFS inside previously excavated boreholes before backfilling them (preferably in accordance with the surrounding stratum type) and assessing through the extracted profiles the occurrence of soil rebound and compaction;
- Superficial soil deformations, induced for example by soil subsidence, by a landslide or by a rockfall, were successfully monitored positioning a high-strength DOFS inside a superficially excavated trench.
- On the DOFS soil-embedment, low performance was detected for acrylate coated DOFS due to their fragility and the poor interfacial coupling with the soil.
- On the use of DOFS for the monitoring of large shear displacements, the suggested procedure was the bonding of DOFS to the soil/rock with heat-shrinking tubes or aluminum block anchors.

On the monitoring of soil stabilization anchors and nails, the most commonly used method was the coupling of one or more DOFS to their tendons. On one occasion, two fiber loops were additionally deployed in the grout material surrounding the anchors. Overall the reported articles testified to a good DOFS strain monitoring performance and the extraction of crucial data on the anchor load-carrying capacity.

Finally, PPP-BOTDA and BOTDR DOFS were successful in the monitoring of movements in the overlying strata and of the advanced abutment pressure during coal seam mining. DOFS was also successfully employed for the monitoring of the rock mass stability conditions in a marble quarry.

For the readers interested in further reading, the authors refer them to the following comprehensive literature review articles on geotechnical [143], geophysical [13] and geo-hydrological [14] DOFS-based SHM.

## 7. Tunnels

There is no doubt that Geotechnical Engineering and Tunnel Engineering go hand in hand. This statement can be even stretched as far as asserting that they are one and the same especially considering that proper geotechnical monitoring systems are crucial for safe underground construction and operations. Nevertheless, keeping in mind the difference in boundary conditions and vast application diversity, for simplicity’s sake the present article distinguishes their DOFS monitoring in two different sections, the previous and the present one.

The ever-growing urbanization throughout the world has been the catalyst for congestion, air pollution and surface scarceness issues. The accompanying awareness that this situation could be alleviated by means of an underground placement of some key facilities (rapid transit, parking, utilities, storage, etc.) has led to an increased tunneling activity. The amount of tunnels is not the only growth factor, though. Their design complexity is rising too, encompassing longer and wider tunnels, excavations through increasingly difficult ground conditions, under densely populated zones (thus requiring very high safety standards) and in highly seismic areas [144].

The need for a performant tunnel SHM is particularly self-explanatory, for example, in the aftermath of a seismic event. especially when attempting to assess its residual capacity to survive consecutive seismic aftershocks. This withstanding, SHM is equally crucial during a tunnel’s construction and service-life. As a matter of fact, both new and existing tunnels suffer an inherent structural risk connected to the surrounding geotechnical and hydrological conditions (new ground level constructions and excessive rain periods amongst others) as well as to unknowns related to design assumptions and construction materials [145]. These risks may translate into the appearance of abnormal horizontal and vertical tunnel deformations, convergence deformations (relative displacement of two diametrically-opposed points), cracks, joint movements, water leaks and, in the worse cases, collapse. The SHM of tunnels and underground structures is not only a key aspect for their correct functioning and safety of use but also for the security and integrity of its surroundings. Indeed, the latter can potentially experience geotechnical condition alterations due to tunneling (especially in the case of shallow tunneling) such as settlement troughs and holes at ground level, groundwater level disruption, urbanizations’ drainage system jeopardizing and more [146].

Whilst the common practice for the detection of the above-mentioned tunnel structural issues (or of their precursor signs) are regular visual inspections, these have very logical performance limitations and often require to stop the tunnel traffic, thus strongly limiting their frequency. Alternatively, geodetic target points can be mounted to a tunnel linings’ surface in order to measure their displacements by means of total stations. However, these measurements also miss out on the internal strain distribution data in addition to being taken averagely only once per day [147]. Finally, discrete and punctual sensors also pack a very limited performance being the location where the structural failures might appear unknown a priori thus impossible to accurately instrument. Distributed sensing, instead, surpasses all the above limitations and positions itself as a permanent and autonomous monitoring solution capable of measuring physical parameters along the whole length of the tunnel with a single transducer.

DOFS’ potential application to tunneling was proved feasible and satisfactory in a few pioneering publications such as 2010’s Shi et al. [148] (who monitored with a BOTDR DOFS the deformations of the extra-long Taiwan Strait Tunnel running over 150 km of seafloor geologic body) and 2015’s Gue et al. [16] (who monitored through BOTDR DOFS London’s Royal Mail tunnel during the construction of the large cross-rail platform tunnel located below it).

Starting the present discussion from the tunnel monitoring during their construction phase, a common building methodology is the New Austrian Tunneling Method (NATM) which is executed through excavation sequences that allow for the surrounding ground/rock to support itself [147]. The method’s first phase consists of the excavation of the top-heading area of the tunnel, later supported by bolts, lattice girders and a reinforced shotcrete (concrete spraying) lining. The second phase consists of the excavation of the bench/invert area which is later supported by more reinforced shotcrete to form a load-bearing ring system.

**Figure 63:**
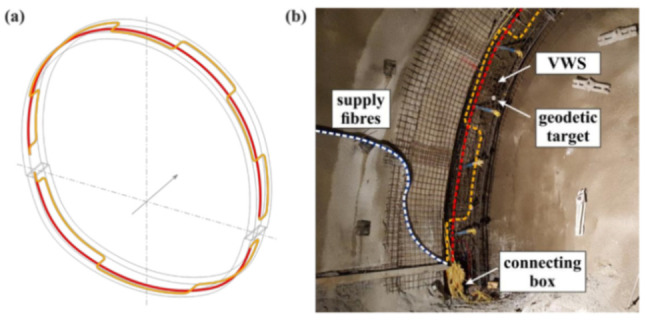
DOFS installation system inside the tunnel lining: (**a**) Schematic representation and (**b**) practical realization [147].

Monsberg et al. [147] reported a comprehensive work on the design and realization of an OFDR DOFS monitoring system where the fiber was installed in the above-mentioned shotcrete linings situated in a fault zone of the Semmering Base railway Tunnel (Austria), at the time under construction following the NATM. The described campaign saw the distributed monitoring of strains and temperature by means of a 230 m long DOFS located on two layers of a study case tunnel cross-section (rock-side and cavity-side as visible in Figure 63).

**Figure 64:**
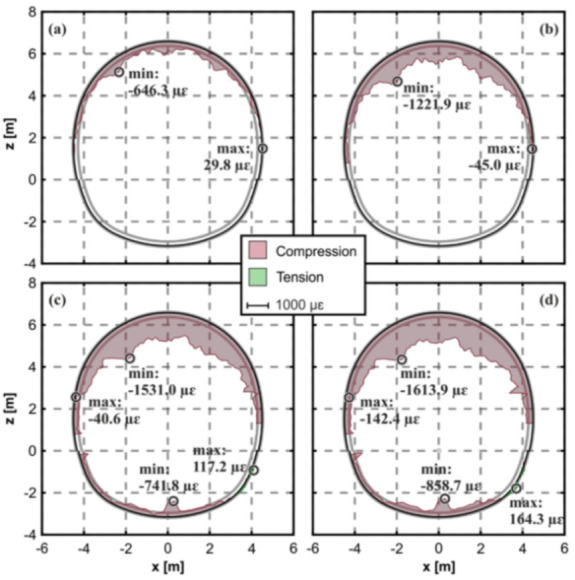
Measured strain distribution at the rock-side layer in peripheral direction: (**a**) 12 h after installation at top-heading, (**b**) 48 h after installation at top-heading, (**c**) 12 h after installation at invert and (**d**) 48 h after installation at invert [147].

The several weeks-long measurements began from the moment the shotcrete started curing and continued over the advancement of the tunnel drive construction. In order to prove the suitability of the DOFS-based monitoring system, the results were compared with the ones coming from Vibrating Wire Sensors (VWS in Figure 63) and geodetic measurements. Approximately 12 h after the construction of the top-heading, the authors reported observing compression zones at the crown and the shoulders (as reported in Figure 64).

Then, 48 h after the construction of the bench/invert, the deformations were reported to be almost constant along the entire supporting ring suggesting that it formed a load-bearing system capable of coping with the remaining loads. The authors concluded by remarking that DOFS allowed to precisely localize the maximum strain within the cross-section instantly (differently than conventional geodetic measurements) along with its deformation history. Yet, they also noted that the position of the sensor versus the direction of the tunnel drive significantly influenced the results.

Whilst the presence of temporary shorings ensure the stabilization of large cross-section tunnels excavated according to the NATM, their consequent dismantling during the secondary lining’s construction process is amongst the most dangerous steps of the whole construction development due to alteration of the tunnel lining’s original structural equilibrium. As such, Li et al. [149] performed a BOTDR DOFS monitoring in order to determine the Beijing Rail Transit airport line tunnel’s deformation behavior during such shoring dismantling stage. A DOFS was used in combination with a metal-reinforced strain-sensing optical cable (purposefully designed for harsh environments). Three DOFS were positioned in three 1 cm deep longitudinal grooves incised in the tunnel primary lining which were later sealed with quick setting high-strength mortar. The sampled measurements showed large settlement variations during all the dismantling steps, the largest of which was 5.7 mm corresponding to a tensile strain variation of 50 με in the crown. Furthermore, the authors were faced with the limitation of being unable to measure directly the tunnel’s horizontal deformation due to the axial DOFS deployment along the tunnel. The limitation was surpassed by extracting an inversion model of the vault settlement curve based on a numerical simulation and the strains obtained using DOFS. In conclusion, the authors estimated a 0.9 mm maximum deviation of the tunnel’s settlement.

**Figure 65:**
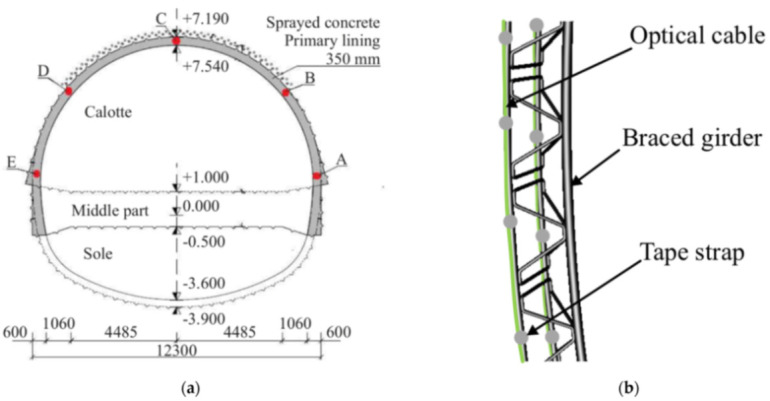
(**a**) Tunnel and distributed temperature and strain system layout and (**b**) optical fiber implementation on iron bars of the braced girder of the primary lining [146].

BOTDR was also employed by Fajkus et al. [146] in order to perform a security monitoring of the structural loads carried by an 867 m long highway tunnel under construction in Žilina (Slovakia) also following the NATM. In this case, too, the DOFS was placed on a tunnel’s braced girder which would later be incorporated in the first of two linings by means of concrete spraying (see Figure 65).

The authors reported the load changes (referred to in the article as changes of the Brillouin frequency) occurring over a period of time of 5 months and hypothesize their origin to be a combination of the temperature variation (of the concrete and the surrounding rocks) and concrete shrinkage.

**Figure 66:**
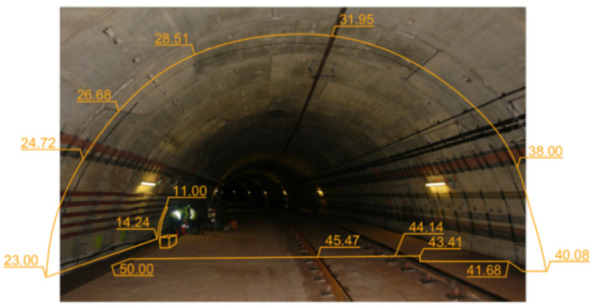
Monitored tunnel section [150].

As mentioned beforehand, the monitoring of tunnels under service is as important as the one during their construction phase. A recent example of the former is reported in Gómez et al. [150]. This article reported the implementation of an OFDR DOFS on the TMB L-9 metro tunnel in Barcelona in order to assess whether it was affected by the construction of a residential building 14 m above it (of which roughly 37 m^2^ could be found right above the tunnel). In particular, a 50 m long fiber was glued to the tunnel’s cross-section expected to suffer the largest deformations (thus defined as critical section), to a segment beneath the tracks and to a segment of the linen parallel to the tunnel (as represented in Figure 66). In this case, the asymmetrical construction-induced loading and unloading effects would not be directly translated in a uniform pressure around the ring due to the additional presence of a bending effect. This was observed during the in-situ monitoring which reportedly detected compression in the linen’s intrados (where the DOFS was actually bonded) during the excavation phase and tension during the building’s construction (when the external load was negative).

For proper analysis and visualization of the data, the authors mentioned having used complex data treatment algorithms [151] to get rid of a significant number of anomalous readings in the raw strain data. The authors concluded by highlighting the need for general, standardized guidelines which also account for the structure’s nature, for the temperature effects and for the adopted post-processing techniques.

Minardo et al. [152] employed distributed sensing for the long-term monitoring of a railway tunnel (originally built in 1880 and reconstructed in 1992) in the accumulation zone of an active earthflow in the Southern Italian Apennines. The 1 cm/year displacing landslide had caused the opening of the joints and the appearance of some thin cracks in the tunnel. The latter had been monitored in recent years by means of a 200 m long BOTDA DOFS hand-glued to its walls. The paper presented the results of such monitoring and in particular the increase in joint opening/misalignment.

Further examples are provided by Inaudi and Walder [145] who reported two distributed sensing-powered tunnel monitoring. The first was deployed in 1968’s San Salvatore Tunnel (Italy) where water accumulation behind a concrete lining had caused its collapse. To prevent this from reoccurring, two Brillouin scattering-based DOFS were bonded to the tunnel linings allowing for the detection and localization (with a 1 m spatial resolution) of any hydrostatic pressure-induced deformation and cracking. The effects of temperature variations (in the order of magnitude of 100–200 με) were accounted for and compensated by means of an extra temperature sensing cable. In the second study case, the average strain distributions along five longitudinal lines of a high-speed rail tunnel in Barcelona (Spain) were monitored. Here, two DOFS were deployed on the side walls and one on its floor, between the two rail lines (summing up to a total monitored length of 12 km).

As seen before in [149,150] the tunnel monitoring is often accompanied by a certain amount of computational and algorithmic work whether this is for validation, inverse-calculation or forecasting purposes.

In Zhao et al. [153] the authors performed a BOTDR DOFS monitoring system of 1984’s Dalian Baiyun Tunnel. Here, nine 6.6 m long optical-fiber stretches were bonded to the tunnel vault with polyimide adhesive and covered with fiber cloth. The authors used the DOFS-extracted strains to study the vault displacement which, in combination with the surrounding rock structure’s data, yielded a Differential Evolution Algorithm capable of carrying out inverse analyses on the major mechanical impact parameters of a tunnel’s lining structure.

Piccolo et al. [154], instead, proposed a DOFS-based methodology for convergence measurements over time of existing tunnels. In particular, starting from experimental orthoradial PPP-BOTDA and Tunable Wavelength Coherent Optical Time Domain Reflectometry (TW-COTDR) DOFS strain measurements, an inverse analysis associated with a finite element model would allow gaining insight into a tunnel’s deformed geometry. In order to understand the practicability of this procedure, the authors performed a laboratory test on a 762 mm diameter steel ring, horizontally fixed at different points to an external reaction frame. The orthoradial strain values were sampled by means of the two DOFS along with displacement sensors. The authors concluded by stating that the DOFS seemed to be sufficiently performant to obtain convergence measurement. They also noticed that, as the testing force grew, DOFS soldered to the steel ring’s surface provided better agreement with reference values compared to the simply glued ones.

The present sub-section saw the DOFS SHM monitoring of both new and older tunnels. In the first scenario most applications were aimed at establishing the deformations of a study case cross section by means of DOFS positioned inside the steel lattice girders during the various stages of NATM construction. The monitoring system allowed to instantly and precisely localize the maximum cross-sectional strains along with its deformation history (over multiple months-long in one of the reported cases). The orientation of the sensors compared to the direction of the tunnel drive was seen to have a large influence. For instance, in one of the reported campaigns, due to the DOFS longitudinal deployment, the use of an inversion model of the vault settlement curve based on a numerical simulation was necessary to establish the tunnel’s horizontal displacements occurring during the removal of temporary shoring. Furthermore, the use of expressly designed resistant DOFS was necessary for a successful sensor embedment inside concrete. This precaution was not necessary for externally bonded DOFS, as was the case for most monitoring of already existent tunnels where the fibers were simply glued to the linens’ intrados. For the latter, the main takeaway was the successful monitoring of minute but relevant deformations induced, for example, by the construction above the tunnel of a residential building or by water accumulating behind a tunnel’s concrete linings or even by the continuous effect of an earthflow occurring above the tunnel.

## 8. Pipelines

Pipelines, whether underground, above ground or subsea, are one of the most important ways to transport across extensive distances key resources such as water, oil, natural gases and telecommunication cables. Furthermore, pipeline transportation is substantially more efficient (financially, environmentally and labor-intensely wise) compared to other ground transportation alternatives such as trucks and trains. Yet, the economic importance of laying and maintaining pipelines is absolutely non-neglectable and should be pondered seriously. Maintenance, in particular, is crucial in those common situations where the pipelines are located in complex and hazardous environments (exposed, for instance, to biological and/or chemical hazards, landslides, soil subsidence and third party interference) which might lead to issues such as pitting and patch corrosion, cracking and to an overall degradation of a pipeline’s structural integrity. The above could even represent a great safety hazard and risk of environmental pollution in case of pipe leaks and bursts. Thus, the constant monitoring of a pipeline’s structural health is crucial to guaranteeing its safe functioning and to averting the substantial economic, environmental and social consequences of its failure.

Generally, the SHM for pipelines demands the observation of their deformation, curvature, eventual leaks and the movements of their surrounding ground. Yet, considering their kilometric lengths and beforehand-unknown failure location, accurate structural monitoring of their entire extension is challenging for discrete sensors. This is not the case for DOFS which completely bypasses the need to install thousands of discrete sensors (with their complex cabling) in order to cover all the potential failure locations. This by itself makes DOFS the ideal pick for a pipeline’s SHM.

This was clearly established by pioneering publications such as Glisic and Yao [155] (who developed a novel Brillouin scattering-based DOFS-based method for real-time, automatic or on-demand assessment of the health condition of buried pipelines after an earthquake) and Inaudi and Glisic [156] (who monitored a 35 years old gas pipeline near Rimini, Italy through a Brillouin scattering-based DOFS).

More recently, Wong et al. [157] attempted to experimentally demonstrate the ability of an OFDR DOFS to monitor the growth of fatigue cracks along a cast-iron pipeline. A study case pipe was subjected to internal cyclic water pressure loadings after its thickness was artificially reduced in a small elliptical patch (leaving a minimum final wall thickness of 3.5 mm) to simulate a severely corroded area. DOFS was deployed on the surface of such a patch from the center of which a longitudinal crack appeared and started to propagate. DOFS effectively detected the latter two phenomena but only when the distance between the sensor and the damage was less than 40 mm. The authors concluded by stressing the importance of the orientation of DOFS and suggesting that it hinges on the minimal critical patch and crack length that is intended to detect.

**Figure 67:**
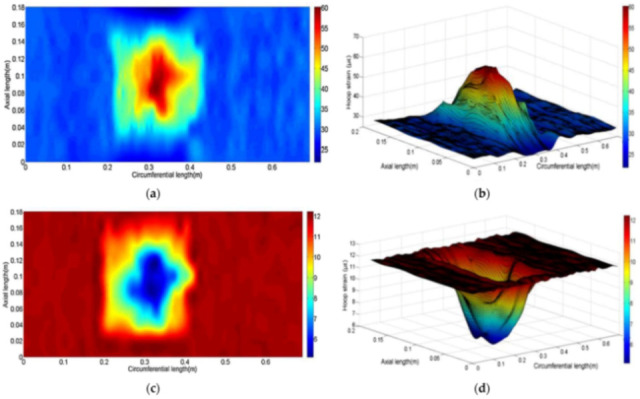
Hoop strain nephogram when the pipe was corroded at 200 h (**a**) 2D and (**b**) 3D and wall thickness nephogram when the pipe was corroded at 200 h. (**c**) 2D and (**d**) 3D. [158].

Ren et al. [158] also attempted to monitor both corrosion and leakage by means of OFDR. In the corrosion test, several optical fiber sensors were bonded to a pipe’s surface in order to form a sensor array capable of monitoring and reporting any corrosion-induced variation in the hoop strains, explicitly strains along the circumferential direction. In the leakage test, instead, the bonded DOFS were intended to detect any leakage-induced internal pressure drop along the pipe. According to the authors, the successful detection of both corrosion (see Figure 67) and leaks implies OFDR’s high efficiency and accuracy for pipeline SHM.

In recent years, pipeline leak detection has also been investigated by means of Distributed optical fiber base Acoustic Sensing (DAS). The latter, also referred to as Distributed Vibration Sensing (DVS), belongs to the latest developments of DOFS technologies and are part of the Rayleigh-based DOFS family. DAS is an ideal fit for pipeline SHM as their base principle, heterodyne coherent optical time-domain reflectometry (Φ-OTDR), grants them the ability to detect highly-dynamic vibration signals up to the kHz range. Here, the elastic waves phase change produced by perturbing objects in the fiber’s proximity is detected by an Acoustic Optic Modulator. Thus, the phase-sensitive OTDR can be used for real-time vibration monitoring of extended objects, e.g., bridges, dams, highways, railway tracks, pipelines, paved paths, and many others.

**Figure 68:**
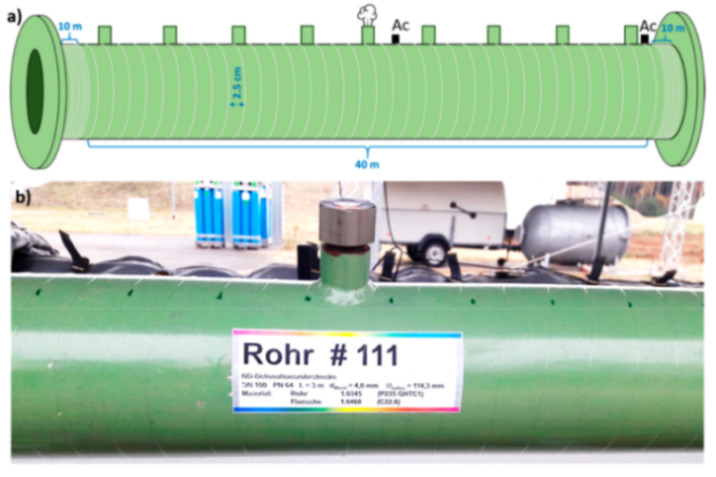
(**a**) Schematic illustration of the fiber helical wrapping around the pipe segments and (**b**) photo of one of the pipe’s side adapters [159].

In 2018, Stajanca et al. [159] performed a helical DAS wrapping around four segments of a 38m long DN100 pipe and simulated leaks using holey adapter caps (with circular holes of different diameters) inserted in the pipe’s side adapter (as in Figure 68).

The goal, studying DAS’ potential to detect a pipe’s weak pinhole leak-induced vibrations for various combinations of leak sizes and pipeline internal pressures. The results revealed that the DAS deployment was capable of detecting the pipeline’s vibration modes induced by the broadband leak-noise excitation. In particular, according to the authors, leaking through increasingly larger hole sizes led to excitation of higher frequencies vibrational modes.

A different DAS deployment methodology is proposed in Zuo et al. [29]. In particular, the authors freely suspended the fiber outside a 12.9 m long Ø0.3 m gas pipeline with the goal of studying the former’s leak detection performance. As a consequence of the deployment methodology, the experiment had to be carried out in a fully muffled chamber with a background noise of less than 40 dB. The authors reported a successful leak detection and localization by means of detailed algorithms based on frequency domain cumulative averaging.

As previously mentioned, along with a pipes’ leak monitoring, its deformation monitoring is also of crucial importance. As such, Feng et al. [160] reported a methodology for monitoring the upheaval buckling of subsea buried pipelines by means of BOTDA DOFS. The authors were faced with the challenge of separately detecting a pipe’s buckling-related bending-induced strains and axial force-induced strains instead of their compound strain. The proposed solution was the longitudinal deployment of three DOFS sensors along a pipe at a radial distance of 2π/3 from each other in addition to a proposed mathematical process. An experimental test was then designed in order to check the performance of such a monitoring scheme. A DOFS-instrumented 5.71 m polyvinyl chloride (PVC) pipe was buried inside standard compacted sand with one of its extremities axially loaded by means of a hydraulic jack. According to the authors, the preliminary results demonstrated the efficiency of the proposed monitoring method despite noting that further research should be dedicated to its performance analysis when including the complex thermomechanical circumstances of buried pipelines.

The deformation of pipelines was also studied by Zhang et al. [161] who instrumented a 4 m long PVC pipeline with two opposite longitudinal runs of BOTDA DOFS and multiple electrical resistance SG in order to verify the former’s monitoring performance superiority for long-distance pipelines. The pipe was gradually subjected to five different loads in its midspan and its displacements were calculated on the grounds of the DOFS-extracted and SG-extracted strains by means of the Conjugate Beam Method. The results led the authors to the conclusion that the measurement accuracy of a pipeline’s displacement is correlated to the number of monitored points thus proving the superiority of DOFS over SG-based for such applications.

Conclusively, multiple DOFS SHM studies were dedicated to the detection of pipeline leaks. This was performed both by means of standard DOFS and DAS. When a standard strain sensing DOFS was bonded locally, in the proximity of a corrosion-damaged patch, the results showed the need of the fiber to be close no more than 40 mm from the damage to actually be able to detect it. Alternatively, DOFS or DAS could be wrapped along the entire extension of the pipe to detect any potential peaking of hoop strains and vibration, respectively, indicative of corrosion and/or leaking. The authors reported successfully detecting and localizing leaks by means of this technique. Alternatively, DAS could be suspended outside of the pipe in order to detect any leak-induced vibrations (if the requirement of low environmental noise can be met). Finally, in order to monitor the occurrence of pipeline deformation, DOFS was deployed on the latter’s surface by means of a double and opposite fiber run or by means of a triple fiber run at a radial distance of 2π/3 from each other. Tests performed with these methodologies both yielded key deformation data.

For further reading, the author suggests the following recently published review article, Inaudi [162].

## 9. Wind Turbines

The popularity of wind turbines as an additional/alternative renewable source of power is steadily increasing over the years, contributing to the reduction of fossil fuel reliance. According to a report from the World Wind Energy Association [163] the wind power capacity worldwide has reached a total energetic value of 597 GW in 2018 with a growth of 9,1%, compared to the previous year and 10,8% versus the one before that. Worth noting is the potential brought forwards by offshore wind power plants. These have the advantages of high wind speed, low wind edge cutting, low turbulence, and high output [164] despite requiring considerable engineering efforts for their design. In addition to these, novel engineering challenges are arising in the past few years as a consequence of the increased size characterizing the newly designed/produced wind turbines (for the sake of increased productivity). Thus, in order to reduce the risk of failure induced by the consequent greater forces acting on the wind turbines’ structures, a comprehensive SHM has become of paramount importance.

Coscetta et al. [164], for example, reported the results of two BOTDA DOFS powered tests aimed at demonstrating the possibility of performing a static and dynamic strain and vibration monitoring of a 14 m long composite wind turbine blade. In the first test, the blade was stressed as a simple cantilever beam (the load was applied in the flap-wise direction) and it was monitored by a fiber attached along the middle of the blade’s upper and lower surfaces. The resulting non-linear strain profiles were explained to be the fruit of the combined effect of the cantilever configuration and the varying geometry of the blade itself. This aside, the authors reported a high level of linearity between the applied load and the measured strains. In the dynamic test, a 100 kg load was applied on the blade’s free end and the latter was put into vibration by means of an electrical motor positioned on it. Expectedly, the amplitude of the extracted strains was larger on the clamped end of the blade than on its free one.

In Raman et al. [165], the authors stated the importance of performing a real-time SHM of the adhesives present in a blade’s trailing edges (joining the upper and lower composite structures), a common failure location. The article’s research efforts were focused on establishing the optimal DOFS positioning technique for multi-direction strain monitoring within the carbon composite plies. In particular, the multi-linear placement was discouraged due to its limited strain sensing direction (only the lateral one) and, in its stead, a dual sinusoidal placement was commended. Indeed, the latter is reportedly able to detect strain signals both in the longitudinal and in the lateral directions allowing the simultaneous characterization of a blade’s bending and torsional loads.

**Figure 69:**
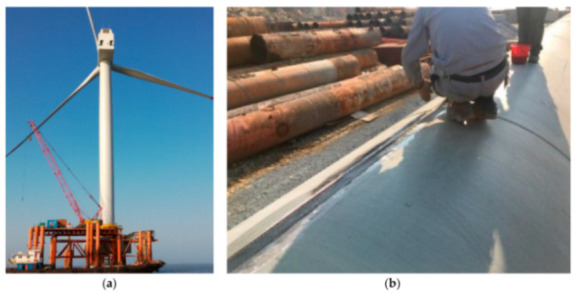
(**a**) Study case offshore turbine (**b**) DOFS bonded with epoxy to the sample pile (adapted from [26]).

The above-mentioned wind turbine blade’s size surge has the additional side effect of increasing the overturning moment withstood by a pile’s foundation. Zhang et al. [26] used a Differential Pulse Pair Brillouin Optical Time-Domain Analysis (DPP-BOTDA) to monitor a 69 m tall offshore wind turbine pile (belonging to the Xinghua Bay Wind Farm in Fujian) in order to obtain the bearing capacity of its foundation (see Figure 69).

**Figure 70:**
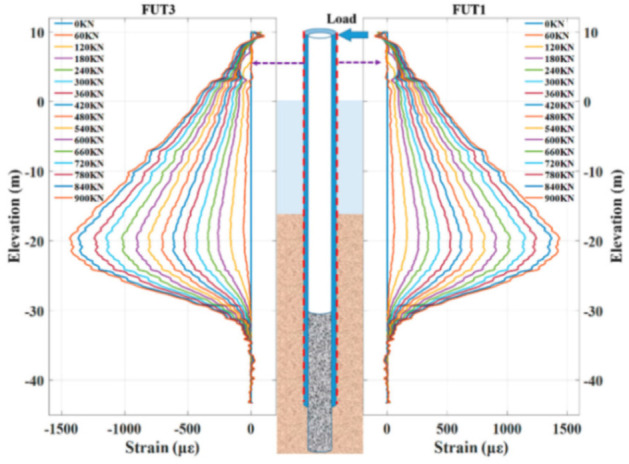
Strain values per corresponding elevation from 0 kN to 900 kN [26].

The monitored concrete-filled/steel composite pile was a test sample mounted on the same offshore platform as the actual study case one. The author’s aim was pin-pointing the pile foundation’s maximum stress location under horizontal loading (applied by means of a horizontal reaction frame). The test, subdivided into 15 consecutive load stages with load increments of 60 kN, yielded the maximum strain (1436 με) at the last load stage at y coordinate −19.63 m (as visible in Figure 70). Expectedly, the maximum displacement (0.79 m) occurred at the uppermost end of the fiber and the load/maximum strain and the load/maximum displacement profiles were both found to be linear in nature. The authors concluded commending the proposed DPP-BOTDA for any offshore wind turbine pile foundation SHM.

Conclusively, it can be stated that few DOFS wind turbines SHM were performed in the latest years. The reported research efforts saw three different DOFS bonding conditions, one on the surface of turbine blades, one inside them (through an innovative deployment technique named dual sinusoidal placement) and one more on the pile. Throughout the articles, both static and dynamic tests were performed, all successfully yielding the sought-after results. As such, all the authors commended DOFS for the SHM of wind turbines.

## 10. An Eye towards the Future

The present section intends to concisely report novel DOFS applications/studies that may very well pioneer new ways of performing SHM. Whilst not all the following applications can be tagged as strictly Civil Engineering-focused, the potential that they bring forth could definitely benefit the field in the near future.

**Figure 71:**
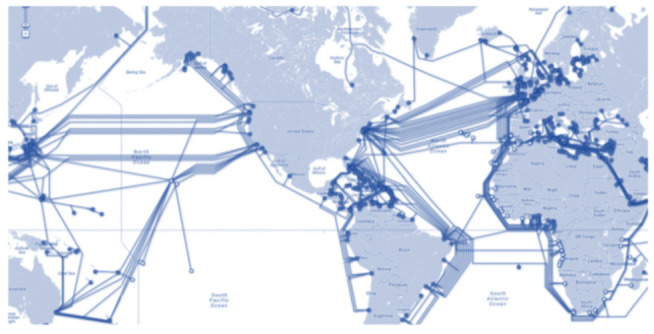
Submarine cable map in 2015 [166].

For example, Fernandez-Ruiz et al. [28] introduced the idea of using the already-existing world-wide telecommunication fiber optic network (Figure 71) for continuous, real-time monitoring of the global surface on the lookout for any seismic activity.

**Figure 72:**
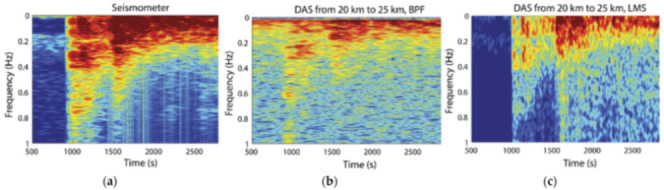
(**a**) Spectrogram of the seismic signal measured by a reference seismograph (**b**) Spectrogram of the DAS data denoised with a 2D linear bandpass filter and a (**c**) Spectrogram of the DAS data denoised with a 1D adaptive LMS filter (adapted from [28]).

By means of the optical fibers’ ubiquity and ability to perform distributed and highly sensitive DAS vibration monitoring, it would be possible to recognize patterns of potentially hazardous seismic activity. In particular, the authors tested the feasibility of such a plan by monitoring a simulated seismic wave with both a seismometer and DAS sensing fibers. The comparison of the extracted seismometer spectrogram and DAS’s output (combined with a linear 2D bandpass filtering technique and a 1D adaptive LMS filter) showed an encouraging time–frequency dynamics similarity (as visible in Figure 72).

The potential of cabled seafloor observatory networks was also discussed in Hartog et al. [167]. Here, the authors debated the potential role of DOFS technology in evolving the traditional single-point isolated oceanographic measurements (performed on the seafloor or on the surface with moorings, landers and arrays of surface floats) by finally filling in the extensive spatial and information gaps existing between the latter. This way, DOFS could help improve the modern understanding of the world’s oceans and of the role they play on issues such as climate regulation, global food supply and energy production.

Additionally, DOFS could permit a vaster-deep seafloor monitoring for the detection of hazards such as earthquakes, seafloor remobilizations, slope instability that may trigger tsunamis, turbidity currents and seafloor expulsion of fluids which all pose a threat to critical seafloor infrastructure including telecommunication networks, offshore infrastructure, oil and gas pipelines and umbilicals, wind farm interarray cables and coastal communities.

DAS was also the focus of Huang et al. [168] who proposed a DOFS-based acoustic environmental safety monitoring scheme for the security of a building’s exterior glass walls/windows. In order to improve the accuracy of the disturbing event recognition, the authors suggested the use of the Wigner bispectrum analysis combined with an extreme gradient boosting tree algorithm. The recognition of eight different 25.3 ms long vibration events was then tested (no disturbance, wind blowing, knocking, window opening, watering, beating, dog barking and aircraft sound) resulting in a recognition rate of 93.3%.

Amongst the many potential applications of DAS, Merlo et al. [30] proposed a unique Φ-OTDR monitoring system buried under different tracks and runways of an airport for the positioning and trajectory calculation of airplanes and other vehicular transportation. This system was intended to integrate the current radar surveillance systems operating in the so-called Precision Runway Monitoring. The gist was that the intensity and spatial extension of an externally-induced fiber scattering perturbation phenomenon could allow distinguishing the originating cause (plane, bus, car and human) and its trajectory. With such purpose, the authors reported the design, development, and characterization of a narrow-linewidth solid-state laser with extra-low-noise properties. The latter feature was aimed at preventing any confusion from the processing algorithm’s part between the laser phase noise and an event occurring along the fiber.

**Figure 73:**
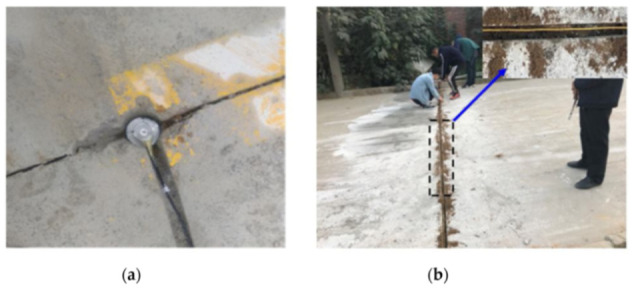
(**a**) Fiber Bragg Grating (FBG)-based acceleration sensor and (**b**) Distributed Optical Fiber Vibration Sensor [52].

Zhang et al. [52] and Liu et al. [169]’s scope was very similar in nature to the above article. This time, though, for vehicular traffic flow monitoring. In the former, multi-scale Distributed Optical Fiber Vibration Sensors, essentially DAS, and FBG were installed on a road pavement to form a load identification system (as in Figure 73).

The authors reported that the test, carried out for four vibration loads (rockfall impact, motorcycle, automobile and concrete tanker), showed that both the DAS and the FBG-based acceleration sensors could successfully identify the different loads.

In Liu et al. [169], instead, the authors suggested the use of DOFS for the detection of traffic vibration signals combined with novel wavelet threshold and dual-threshold algorithms. The efficiency of such a method was later tested with a DAS-powered vehicle-counting and vehicle-speed determination test in the Nanshan Iron Mine (China) where the optical fiber was located on the road shoulder closest to the traffic lane. The test results were reportedly encouraging with an accuracy error of less than 6%.

Moving on from macroscale DOFS applications, Li et al. [170] collected the base concepts behind several novel optical fibers for distributed sensing. Among these, worth mentioning is the idea (originally introduced by Bao et al. [171]) of using sensing cables with two embedded fibers with different Brillouin frequency shifts. This would permit the separation of the temperature and strain effects on the Brillouin frequency shift thus preventing their stacking in the output analysis phase.

You et al. [172] also brought forth a very interesting proposal for a novel OFDR-DOF sensing tape aimed at guaranteeing the survival of the DOFS in harsh construction and service environments. In particular, the authors suggested a packaging technique that traps DOFS between two fiberglass tapes glued with an adhesive. The strain transfer mechanism was analyzed in detail followed by a calibration test and a real-life test that saw its application to the surface of an RC beam subjected to three-point bending. The extracted results reportedly showed the correct functioning of the sensing tape on concrete and its ability to localize cracks.

**Figure 74:**
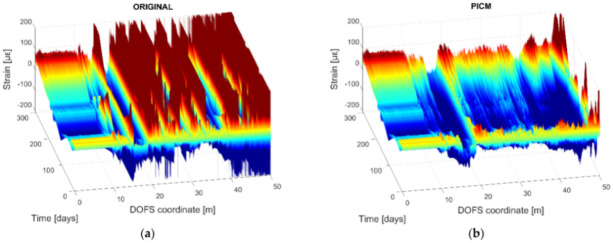
DOFS extracted strain evolution in time: (**a**) before post-processing and (**b**) after post-processing with PICM—Polynomial Interpolation Comparison Method.

When it comes to modern-day OBR interrogators and BOTDA, the use of post-processing algorithms is an important step for the proper study of the extracted strain data. Three examples of these are provided by Bado et al. [151], Heinze and Echtermeyer [27] and Song et al. [173]. In the former, the authors put into practice two previously established algorithms and a novel one for the elimination of SRAs risen in two different monitoring campaigns. The first of these was a laboratory experimental campaign performed on two RC ties whilst the second was in-situ monitoring of a tunnel deformation. The authors reported the presence of large performance discrepancies between the three analyzed algorithms. Indeed, the classic methods (Spectral Shift Quality and Geometrical Threshold Method) provided results with questionable quality despite their quick and small computational taxing analysis. Instead, the novel but more computationally taxing one (Polynomial Interpolation Comparison Method) provided much smoother and reliable output plots as evident in Figure 74.

In Heinze and Echtermeyer [27], a novel post-processing methodology was introduced as a solution to the issue of “meaningless results” (SRAs) being reported by OBR analyses whenever OFDR DOFS pinching or micro-bending occurred. The latter reportedly is the typical case for DOFS embedded inside hardening and shrinking materials. In particular, the new data analysis method baptized “running reference analysis method”, suggested that each measurement should be compared to their previous one and that their strain differences should be added up to the absolute strain value (the original 0 load reference measurement).

Finally, Song et al. [173] argued that whenever BOTDA DOFS are employed for the detection of large cracks inside any study material, these lead by definition to large Crack Opening Displacements which can be easily detected in the fiber’s output as strain peaks or singularities. Yet, when it comes to microcracks, their formation does not bring along any pronounced or even visible peak. On the contrary, a microcrack-induced peak could be easily lost in the midst of measurement noise due to its low signal-to-noise ratio. In order to prevent this from happening and in return improve the crack detection, the authors proposed a novel deep learning method trained and validated by means of an experimental campaign performed on a 15 m-long wide-flange steel beam with artificial defects. The authors concluded by reporting that Crack Opening Displacements as small as 23 microns were successfully detected with the proposed method.

## 11. Conclusions

The present work was intended to introduce, inform and advise the readers on various Distributed Optical Fiber Sensors (DOFS) deployment methodologies for Structural Health Monitoring (SHM) purposes, i.e., the assessment of the residual ability of a structure to continue serving its intended purpose. By collecting in a single article the most recent efforts, advancements and findings on such DOFS SHM integrations the authors intend to contribute to the goal of collective growth towards an efficient SHM.

The current work analyzed numerous DOFS applications such as laboratory experimentation, the built environment (bridges, buildings, roads, etc.), geotechnical constructions, tunnels, pipelines and wind turbines. Relevant specific conclusions are reported at the end of each application section. A common conclusion shared by the largest portion of the reported articles is the efficiency and performance of DOFS for the monitoring of the structures under both serviceability and ultimate conditions. To a certain extent, this shows the instrumental maturity already achieved in some Civil Engineering areas. For instance, in the case of concrete structures, it was shown how the technique is fully operative in the field of crack monitoring both in the controlled environment of a laboratory and in-situ. Crack detection, mapping and width quantification was achieved both in bending and shear. The technique’s efficiency was also fully demonstrated for the calculation of deflections by the integration of measured curvatures and for the distribution of temperatures inside the concrete. Furthermore, DOFS deployments were also shown to provide relevant results in the monitoring of corrosion in the reinforcing steel. Overall, DOFS were commendable SHM tools for the monitoring of geotechnical and underground structures too (foundation piles, soil anchors and tunnels amongst others).

Multiple deployments techniques were described for each kind of structure and for the varied sought-after results. For example, different deployment techniques were suggested for the monitoring of cast-in-situ RC piles versus prefabricated ones or for the cross-sectional versus longitudinal monitoring of a tunnel linen’s intrados or even for the detection of steel pipe leaks versus axial deformations. Furthermore, the increasingly numerous types of commercially available DOFS cables allowed for the surpassing of the problem of fiber fragility and the need of protecting it during the concrete pouring operations as well as increasing their resistance to the high temperatures that may appear during the concrete hardening process or in the case of their embedding into hot asphalt pavements.

The increasingly larger number of applications in each of the reported fields showed a growing interest and recognition of DOFS’ potential, yet, a large number of such publications called for a deeper understanding of the mechanical performance (short-term and long-term) of the multi-layered system with which the fibers are bonded to a structure. Indeed, the strain transfer from a host structure to a DOFS core was seen to depend on the mechanical and geometrical properties of the different intermediate layers, i.e., coating, jacket, reinforcements, adhesives, etc. Therefore, whilst extensive recommendations are now available on optimal bonding/protective materials and on embedding procedures, further work is required for the interpretation of the extracted strain profiles. Additionally, more research is still needed on the DOFS long-term applicability. Indeed, few studies have addressed their performance to fatigue and under cyclic loading, a condition that may affect the behavior of the bonding agents and of the fiber itself. Durability aspects are also under current investigation branching in two main directions: (1) the ability to monitor material degradation (2) the degradation of the fiber and bonding agents themselves when subject to harsh and aggressive environments (chloride and alkaline contamination, etc.).

Overall, though, with the already existent potential of DOFS, their number of routine industrial applications for SHM is certain to increase.

## Figures and Tables

**Table 1:** Acronyms used in the present article and literature references.

Acronym	Name	References
SHM	Structural Health Monitoring	
RC	Reinforced Concrete	
DIC	Digital Image Correlation	
SG	Strain Gauges	
LVDT	Linear Variable Displacement Transformers	
SRA	Strain Reading Anomalies	
DOFS-related		
OFS	Optical Fiber Sensors	[1,13,14,15]
DOFS	Distributed Optical Fiber Sensors	[1,13,14,15]
OTDR	Optical Time Domain Reflectometry	[1,16,17]
BOTDR *	Brillouin Optical Time Domain Reflectometry	[16,17,18]
BOTDA *	Brillouin Optical Time Domain Analysis	[19,20,21]
BOFDA *	Brillouin Optical Frequency Domain Analysis	[22]
PPP-BOTDA *	Pulse Pre Pump-BOTDA	[23,24,25]
DPP-BOTDA *	Differential Pulse Pair-BOTDA	[26]
OFDR *	Optical Frequency Domain Reflectometry	[1]
OBR	Optical Backscattered Reflectometer	[27]
DAS *	Distributed Acoustic Sensing	[28,29]
DVS *	Distributed Vibration Sensing	[28,29]
Φ-OTDR *	Phase-OTDR	[29,30]
FBG *	Fiber Bragg Grating	[9,31,32]

* different kinds of OFS.

**Table 2:** Performance differences between the various distributed sensing techniques [1].

Sensing Technology	Transducer Type	Sensing Range	Spatial Resolution	Main Object of Measurement
Raman OTDR	Distributed	1 km–37 km	1 c–17 m	Temperature
Brillouin BOTDR	Distributed	20–50 km	≈1 m	Temperature and Strains
Brillouin BOTDA	Distributed	150–200 km	2 cm (2 km)–2 m (150 km)	Temperature and Strains
Rayleigh OFDR/DAS	Distributed	50–70 m	≈1 mm	Temperature, Strains and Vibration
FBG	Quasi-distributed	≈100 channels	2 mm (Bragg length)	Temperature and Strains and displacement

## Data Availability

Not applicable.
